# Physics-driven self-supervised learning for fast high-resolution robust 3D reconstruction of light-field microscopy

**DOI:** 10.1038/s41592-025-02698-z

**Published:** 2025-05-12

**Authors:** Zhi Lu, Manchang Jin, Shuai Chen, Xiaoge Wang, Feihao Sun, Qi Zhang, Zhifeng Zhao, Jiamin Wu, Jingyu Yang, Qionghai Dai

**Affiliations:** 1 Department of Automation, Tsinghua University, Beijing, China; 2 Institute for Brain and Cognitive Sciences, Tsinghua University, Beijing, China; 3 Beijing Key Laboratory of Cognitive Intelligence, Tsinghua University, Beijing, China; 4 IDG/McGovern Institute for Brain Research, Tsinghua University, Beijing, China; 5 Zhejiang Hehu Technology, Hangzhou, China; 6 Hangzhou Zhuoxi Institute of Brain and Intelligence, Hangzhou, China; 7 School of Information Science and Technology, Fudan University, Shanghai, China; 8 School of Electrical and Information Engineering, Tianjin University, Tianjin, China; 9 Shanghai Innovation Institute, Shanghai, China; 10 Department of Gastroenterology and Hepatology, Tongji Hospital, School of Medicine, Tongji University, Shanghai, China; 11 Beijing Visual Science and Translational Eye Research Institute (BERI), Beijing, China; 12 Beijing National Research Center for Information Science and Technology, Tsinghua University, Beijing, China

**Keywords:** Microscopy, Fluorescence imaging

## Abstract

Light-field microscopy (LFM) and its variants have significantly advanced intravital high-speed 3D imaging. However, their practical applications remain limited due to trade-offs among processing speed, fidelity, and generalization in existing reconstruction methods. Here we propose a physics-driven self-supervised reconstruction network (SeReNet) for unscanned LFM and scanning LFM (sLFM) to achieve near-diffraction-limited resolution at millisecond-level processing speed. SeReNet leverages 4D information priors to not only achieve better generalization than existing deep-learning methods, especially under challenging conditions such as strong noise, optical aberration, and sample motion, but also improve processing speed by 700 times over iterative tomography. Axial performance can be further enhanced via fine-tuning as an optional add-on with compromised generalization. We demonstrate these advantages by imaging living cells, zebrafish embryos and larvae, *Caenorhabditis* *elegans*, and mice. Equipped with SeReNet, sLFM now enables continuous day-long high-speed 3D subcellular imaging with over 300,000 volumes of large-scale intercellular dynamics, such as immune responses and neural activities, leading to widespread practical biological applications.

## Abstract (web summary)

SeReNet is an accurate, generalizable, and efficient physics-driven self-supervised reconstruction network for light-field microscopy that enables day-long high-speed 3D intravital imaging of processes including immune responses and neural activity.

## Main

Computational imaging has revolutionized optical microscopy in areas such as super resolution^1–5^, optical sectioning^6,7^ and volumetric imaging^8,9^, but it is heavily dependent on reconstruction algorithms. For example, light-field microscopy (LFM) achieves unprecedented spatiotemporal resolution after three-dimensional (3D) reconstruction, facilitating sustained neural recordings and dynamic morphological imaging with low phototoxicity in vivo^9–15^. However, traditional reconstruction methods, mainly based on handcrafted Richardson–Lucy (RL) deconvolution^15–19^, are computationally expensive and prone to artifacts^20^. For instance, in scanning LFM (sLFM^15^) and virtual-scanning LFM (VsLFM^21^), the 3D deconvolution process can take days to reconstruct thousands of frames and requires parameter modification for different system configurations^22^. Moreover, existing LFM techniques suffer from the missing cone problem, which reduces axial performance, particularly in layers far from the native image plane^23^.

Recently, many supervised deep-learning methods, such as CARE^24^, VCD-Net^25^ and HyLFM-Net^26^, have been developed to substantially reduce the computational costs for practical applications. However, these methods still suffer from low spatial resolution and poor generalization in diverse sample structures or complex imaging environments. Moreover, they are not specifically optimized for sLFM data, limiting their fidelity. Dependency on ground-truth data is also a challenge in supervised learning. The training data pairs are not widely accessible, and the diversity of the samples is limited, so pretrained models often underperform on unseen data. Although imaging formation processes have been introduced for better interpretability and generalization^27–29^, they are difficult to apply to the four-dimensional (4D) measurements in LFM because there are phase correlations between different angular measurements used for aberration correction or noise reduction in complicated imaging environments^15,21^. If these 4D light-field measurements are treated merely as a series of separated two-dimensional (2D) images, the phase correlation will be lost, resulting in the degradation of deep-learning methods compared with iterative tomography in intravital environments. Moreover, accurate wave-optics-based point spread functions (PSFs) in the spatial-angular domain are crucial for high-resolution 3D reconstruction (Supplementary Fig. 1). Therefore, developing rapid, high-resolution 3D reconstruction algorithms for LFM and its variants without relying on data supervision remains a pivotal challenge for broad practical applications of LFM-based technologies in diverse complicated imaging conditions. No learning-based reconstruction technique has been developed yet, particularly for sLFM.

Here, we present SeReNet, a physics-driven self-supervised reconstruction network for LFM and its variants. By leveraging the 4D imaging formation priors of LFM, SeReNet achieves near-diffraction-limited resolution at millisecond-level processing speed without the requirement of training data pairs. SeReNet is trained in a self-supervised manner by gradually minimizing the loss between the forward projections of network estimation along 4D angular PSFs and the corresponding raw measurements. For broad generalizations of the reconstruction performance in complicated imaging environments, we fully integrate the imaging process in network training. This approach prevents overestimation of unknown information that the imaging system inherently cannot capture while accounting for the large freedoms provided by the 4D measurements, noises, non-rigid sample motions, and sample-dependent aberrations. An axial fine-tuning strategy can be integrated into SeReNet as an optional add-on to address the missing-cone problem and improve axial performance at the cost of slightly compromised generalization capability. Various benchmarks were conducted in both numerical simulations and experimental conditions, demonstrating that SeReNet outperforms recent state-of-the-art (SOTA) methods in speed, resolution, processing throughput, generalization capability and robustness to noise, aberrations and motions. SeReNet can be integrated in both unscanned LFM and sLFM, achieving processing speeds up to 700 times faster than that of iterative tomography. Compared with supervised neural networks in unscanned LFM, SeReNet achieves better performance when applied to distinct sample types or data from distinct microscopes owing to its superior generalization capability.

Equipped with SeReNet, sLFM facilitates versatile high-speed subcellular 3D observations in vivo with day-long durations in diverse animals including zebrafish (*Danio rerio*) embryos, *Dictyostelium discoideum*, *Caenorhabditis elegans*, zebrafish larvae and mice. Processing the massive tens of terabytes of data, with more than 300,000 volumes, produced from imaging these animals would take several years for previous iterative algorithms with high fidelity; however, SeReNet requires only 5 days, with even better axial performance. These advantages have allowed us to perform long-term monitoring of diverse subcellular dynamics during multiple liver injuries and conduct large-scale day-long cell tracking of immune responses. We believe that, with its broad generalization, low computational costs and high fidelity, SeReNet will lead to widespread practical applications of LFM and its variants in diverse fields such as neuroscience, immunology and pathology.

## Results

### Principle of SeReNet

**Fig. 1:**
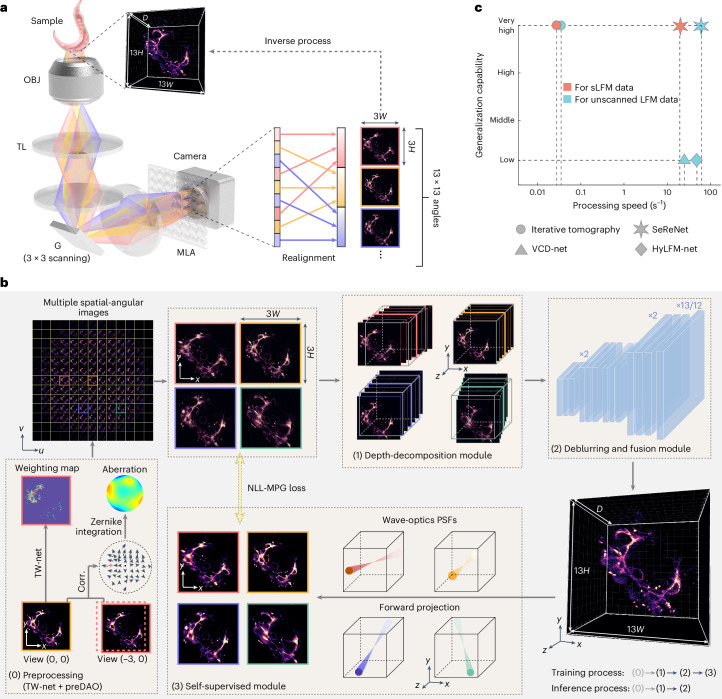
Principle of SeReNet. **a**, The imaging and reconstruction process of sLFM. Fluorescent signals from different angles, indicated by different colors, are captured by sLFM with an angular resolution of 13 × 13. The 3D sample (with a voxel number of *D* × 13*H* × 13*W*, where *D* is usually over 100 for axial sectioning) is encoded into 3 × 3 scanning light-field images (3 × 3 × 13*H* × 13*W*) and realigned into multiple spatial-angular views (13 × 13 × 3*H* × 3*W*). The reconstruction process is the inverse of the imaging process. OBJ, objective; TL, tube lens; G, galvo; MLA, microlens array. **b**, The processing pipeline of SeReNet with self-supervised training. Before network training, data are preprocessed using TW-Net and preDAO. TW-Net corrects sample motion (details in Supplementary Fig. 13a), whereas preDAO estimates and corrects optical aberrations (details in Supplementary Fig. 14a). Using the main modules of SeReNet, we first generated a focal stack with digital refocusing of multiple angular images with the depth-decomposition module, and then gradually transformed the stack into a volume with the deblurring and fusion module. Next, the 4D wave-optics PSFs were used to achieve forward projections of the 3D estimation. Finally, the loss between projections and raw measurement was iteratively reduced during training. The NLL-MPG loss was derived as the loss function (details in Supplementary Fig. 11a). After the model is trained, SeReNet can make rapid predictions without the forward projection process. Four representative angular views are shown for simplicity. **c**, Comparisons of the generalization capability and processing speed on reconstructing timelapse unscanned LFM and sLFM data (429 × 429 × 101 voxels for each volume) among SeReNet, iterative tomography, VCD-Net and HyLFM-Net. More detailed information is provided in Supplementary Table 2. SeReNet offers a runtime over 700 times faster than iterative tomography and better generalization over supervised networks.

The direct mapping from 4D multi-angular light-field images (*x*–*y* spatially and *u*–*v* angularly) to the 3D volume (*x*–*y*–*z* spatially) is ill-posed (Fig. 1a), because the number of measurement pixels is smaller than the number of reconstructed volume voxels. Therefore, previous supervised methods based on data priors could easily converge towards local optimum, leading to low generalization^25,26^. Such a problem becomes even worse, especially in complex imaging environments, owing to the large difference between the imaging formation process in training data and testing data. By contrast, iterative tomography with digital adaptive optics (DAO) fully exploits the high-dimensional freedom provided by 4D measurement of sLFM to achieve high-fidelity 3D reconstruction by better describing the whole imaging formation process in 4D, although it comes with large computational costs in iterative updates^15,22^. Moreover, unknown information not captured by the imaging system could be filtered out during the forward projection using PSF priors, which imposes physical constraints without relying on extensive data priors. Building on this concept, SeReNet harnesses 4D spatial-angular imaging formation priors in a self-supervised neural network, achieving high performance at millisecond-level processing speeds without the need for training data pairs (Fig. 1b,c).

The structure of SeReNet is divided into three main modules (Supplementary Fig. 2 and Supplementary Table 1). First, the depth-decomposition module employs image translation and concatenation operators to generate the initial 3D focal stack lacking optical sectioning from 4D light-field measurements (Supplementary Fig. 2a). By leveraging multiple angular PSFs to distinguish structures at different depths in the 3D volume, the depth-decomposition module explicitly utilizes the PSF information, enhancing the axial performance after reconstruction (Supplementary Fig. 3a). Second, the deblurring and fusion module, consisting of nine 3D convolutional layers and three linear interpolation layers (Supplementary Fig. 2b), is trained to generate a 3D estimation from refocused volumes. This module can effectively recover high resolution, as demonstrated by the ablation study (Supplementary Fig. 3b). Third, and most importantly, the self-supervised module performs forward projections of the estimated volume along multiple angular PSFs, minimizing the loss between projections and corresponding angular measurements in each iteration (Supplementary Fig. 2c). This module prevents SeReNet from incorporating unknown information not captured by the imaging system, as indicated by the PSF, thereby fostering SeReNet’s broad generalization capability. Compared with supervised networks such as VCD-Net and HyLFM-Net, SeReNet will not overestimate or guess uncaptured information, which in this case eliminates artifacts and the risk of overfitting sample textures. If we remove the self-supervised module, SeReNet can be trained in a fully-supervised way, similar to VCD-Net and HyLFM-Net, by directly computing loss functions by comparing 3D predictions with the 3D sample. These supervised processes attempt to infer information that is not captured by the imaging system itself, leading to reduced generalization (Supplementary Fig. 3c–g).

The three-module framework allows the network to gradually converge to a valid solution, with a parameter number of 195,000 for high-speed processing (Supplementary Fig. 4). After being trained with physical constraints, SeReNet can make rapid predictions on sLFM measurements using the first two modules, which have already learned the inverse mapping from light-field measurements to 3D volumes. The reconstruction speed of SeReNet is nearly three orders of magnitude faster than that of iterative tomography (Supplementary Table 2). More importantly, SeReNet exhibits network interpretability because the depth-decomposition module leverages multiple angular PSFs, and each intermediate feature layer accurately reflects physically understandable information in the deblurring and fusion module (Supplementary Fig. 5). Moreover, the high-dimensional property of sLFM cannot be simply replaced by mathematical RL operators in Richardson–Lucy network^27^ (RLN) or straight-line light propagation with simple scaling in neural radiance field (NeRF)-based methods^30^, because they do not contain wave-optics PSF constraints to prevent network overfitting, especially for generalized applications (Supplementary Figs. 1, 6 and 7).

### Advantages of SeReNet with comprehensive benchmarking

To maximize the physics-driven capabilities of SeReNet, we optimized the self-supervised framework to enhance its robust performance under complex imaging conditions. We then conducted both numerical simulations and experimental characterizations to demonstrate SeReNet’s advantages over previous methods, including an ablation study covering noise levels, sample motions, aberrations, and sample diversity.

First, to characterize the resolution of SeReNet, we imaged 100-nm-diameter fluorescence beads (Supplementary Fig. 8). We compared SeReNet with iterative tomography^15^. The measured full widths at half-maximum (FWHMs) revealed that SeReNet achieved a resolution of ~220 nm laterally and ~420 nm axially, approaching the diffraction limit. Although all methods experience a slight degradation in resolution as the axial defocus distance increases, SeReNet still demonstrated more uniform performance across the axial coverage, offering an extended depth of field (Supplementary Fig. 8d,e). Even when the system reverts to traditional LFM, SeReNet still maintains better resolution than other methods, including VCD-Net^25^ and HyLFM-Net^26^ (Supplementary Fig. 9). Considering its short processing time and high resolution, SeReNet achieves about two-times-higher processing throughput than do other SOTA approaches (Supplementary Table 2 and Methods). Furthermore, SeReNet is effective in sLFM with different scanning numbers and maintains stable performance, whereas previous learning-based methods are not designed for sLFM (Supplementary Fig. 10).

**Fig. 2:**
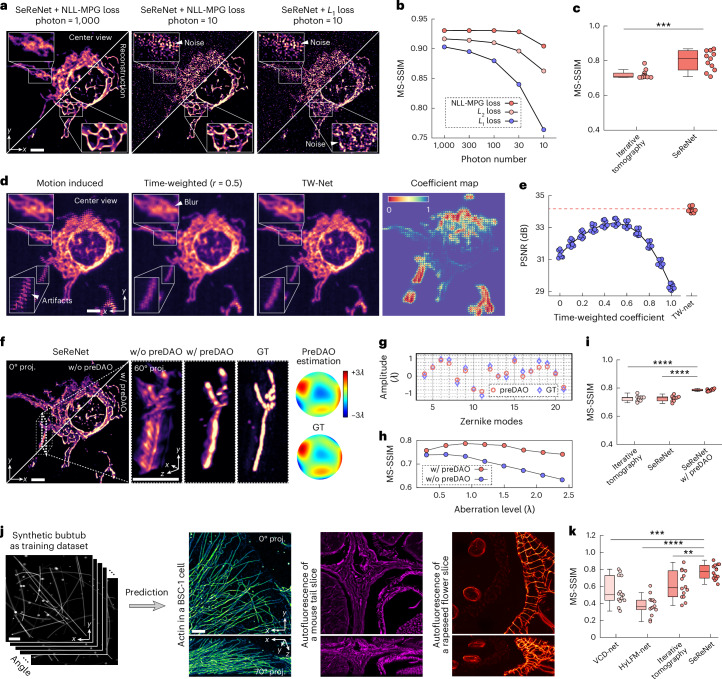
Evaluation and benchmarking of the robustness and generalization of SeReNet. **a**, Raw measurements and SeReNet reconstruction results of a mitochondria-labeled L929 cell with different levels of mixed Poisson–Gaussian noises applied in simulation. NLL-MPG loss and *L*_1_ loss are compared. **b**, Multiscale structural similarity (MS-SSIM) curves over photon numbers, comparing different loss functions. **c**, Boxplot showing MS-SSIM indices obtained by different methods under low-photon (5–15) conditions. *n* = 11 experiments. *P* = 7.78 × 10^−4^. **d**, Measurement with artificially induced non-rigid motion and its counterparts, corrected by time-weighted algorithm and TW-Net. The coefficient map estimated by TW-Net is shown. **e**, Peak SNR (PSNR) curve versus different methods and coefficients. *n* = 9 views are shown as scatter points. **f**, SeReNet results without (w/o) and with (w/) preDAO after the input was contaminated by an induced aberration wavefront, the root mean square (r.m.s.) of which was set to one wavelength. The estimated wavefront by preDAO and ground truth are attached. GT, ground truth; *λ*, wavelength. **g**, Visualization of the amplitudes of 18 Zernike modes decomposed from the estimated pupils by preDAO (red) and the ground truth (blue). **h**, MS-SSIM curves versus aberration levels with and without preDAO. **i**, Boxplot showing MS-SSIM indices obtained by different methods with severe aberrations. The r.m.s. was set to one wavelength. *n* = 10 aberration patterns were used. *P* = 1.42 × 10^−6^, 1.50 × 10^−6^ from left to right. **j**, Test of generalization from the bubtub dataset to multiple kinds of experimentally captured structures. **k**, Boxplot showing MS-SSIM indices obtained by different methods, compared with the ground truth. *n* = 14 represents the number of samples. *P* = 1.01 × 10^−4^, 3.06 × 10^−10^, 5.10 × 10^−3^ from left to right. In boxplots: center line, median; box limits, lower and upper quartiles; whiskers, 1.5 × interquartile range. Asterisks represent significance levels tested with two-sided paired *t*-test, significance at *P* < 0.05. **P < 1 × 10^−2^; ***P < 1 × 10^−3^; ****P < 1 × 10^−4^. All networks were trained on synthetic bubtub dataset. Scale bars, 10 μm (**a**,**d**,**f**,**j**). Source data

Second, noise in optical microscopy, typically characterized by a mixed Poisson–Gaussian (MPG) distribution dominated by the Poisson component, is inevitable^31^. To address this, we derived a negative log-likelihood loss function based on MPG distribution (NLL-MPG loss) for SeReNet (Fig. 1b, Supplementary Fig. 11a and Methods). Under low signal-to-noise-ratio (SNR) conditions, the NLL-MPG loss increased fidelity in distinguishing intricate organelles (Fig. 2a). Our method shows more stable performance than other loss functions (Fig. 2b and Supplementary Fig. 11b–d) and reconstruction methods (Fig. 2c and Supplementary Fig. 12), even in the case of a very low SNR with only a few dozen photons.

Third, the dynamics of cells and organelles can induce ‘checkerboard’ or ‘stripe’ artifacts during scanning in sLFM^22^. We developed a lightweight content-aware time-weighted network (TW-Net) embedded in SeReNet to automatically correct spatially non-uniform motions on the basis of optimal weighting of surrounding pixels (Fig. 1b, Supplementary Fig. 13a and Methods). Although previous time-weighted algorithms^15^ could also reduce motion artifacts but with reduced fidelity and dependency of hyperparameters, TW-Net-assisted SeReNet offers superior resolution, speed and user convenience (Fig. 2d,e and Supplementary Fig. 13b–g).

Fourth, tissue heterogeneity and imperfect imaging systems will distort wavefronts of light, especially in intravital imaging^32^. These aberrations will alter the imaging process by affecting the PSFs, which can degrade the performance of supervised neural networks without a good generalization capability. We designed a DAO^15^ preprocessing (preDAO) module for SeReNet to estimate and correct optical aberrations on the basis of 4D measurements (Fig. 1b, Supplementary Fig. 14a and Methods). Instead of iterative aberration estimation, we developed a non-iterative strategy for accurate wavefront estimations across different aberration levels (Fig. 2f–h). Compared with previous methods, SeReNet with preDAO showed greater robustness to optical aberrations and significantly improved speed (Fig. 2i and Supplementary Fig. 14b–e). This advantage is crucial for robust performance in complex intravital imaging environments by fully exploiting the 4D property of light-field measurements (Supplementary Fig. 14f).

Fifth, practical experiments yield diverse image structures across different species and cells. To improve the generalization ability, the self-supervised module in SeReNet combines 4D image-formation priors into the network training process to prevent overfitting of the 3D information not captured by the imaging system in previous supervised networks. In this case, SeReNet can be trained solely on simulated data and directly generalized to diverse experimental samples. Its performance is comparable to the result obtained by training it directly on the experimental sample, greatly reducing the dependence on experimental datasets (Supplementary Fig. 15). Therefore, we constructed a simulated dataset named ‘bubtub’, which contains the sLFM images of various geometric structures such as bubbles, beads and tubes, with different densities, diameters and optical aberrations (Supplementary Fig. 16a). The bubtub dataset mainly provides substantial structural information, enhancing dataset diversity and serving as a basis for the network to learn physical priors through the 4D image-formation convolution with multiple angular PSFs. During experiments, we found that SeReNet, trained with bubtub, generalized to diverse experimental biological structures with high fidelity (Fig. 2j). It outperformed other supervised networks, which struggled with aberration mismatches between training and testing (Fig. 2k and Supplementary Fig. 16b,c).

Finally, SeReNet is compatible with data-driven supervised networks. We incorporated a subtle data prior into the pretrained self-supervised network to enhance axial performance and alleviate the missing cone problem (Supplementary Fig. 17a and Methods). By simulating three 5-μm-diameter spherical shells, we characterized the optical sectioning capability for different methods. Axially improved SeReNet reduced axial tailing and achieved better optical sectioning by exploiting data priors (Supplementary Fig. 17b,c). In addition, the axially improved SeReNet is built on the self-supervised SeReNet pretrained model, with only a small dataset and epochs used for supervised axial fine-tuning, retaining better generalization than fully supervised networks (Supplementary Fig. 18). However, the improvement in axial resolution still induces a slight degradation of generalization^24^, because the enhanced axial information is not captured by the imaging system, leading to a balance between the increased axial resolution and generalization. Therefore, the axial fine-tuning strategy is designed as an option of SeReNet, allowing users to flexibly choose their own configurations between this tradeoff depending on experimental requirements.

All these strategies have been integrated into SeReNet to maximize its usability across various applications. In this paper, SeReNet was trained on the bubtub dataset with NLL-MPG loss, TW-Net and preDAO, and was utilized for both validations and applications. Details regarding the application of axial fine-tuning are provided in the figure legend.

### Experimental validations in diverse living model organisms

Although synthetic data and fixed biological samples have been used to demonstrate the advancements of SeReNet, verifying its practical utility with time-lapse data that capture real biophysiological processes is essential for broader applicability. To this end, we captured time-series sLFM images in diverse samples and reconstructed them using the SeReNet model trained only on the simulation dataset.

**Fig. 3:**
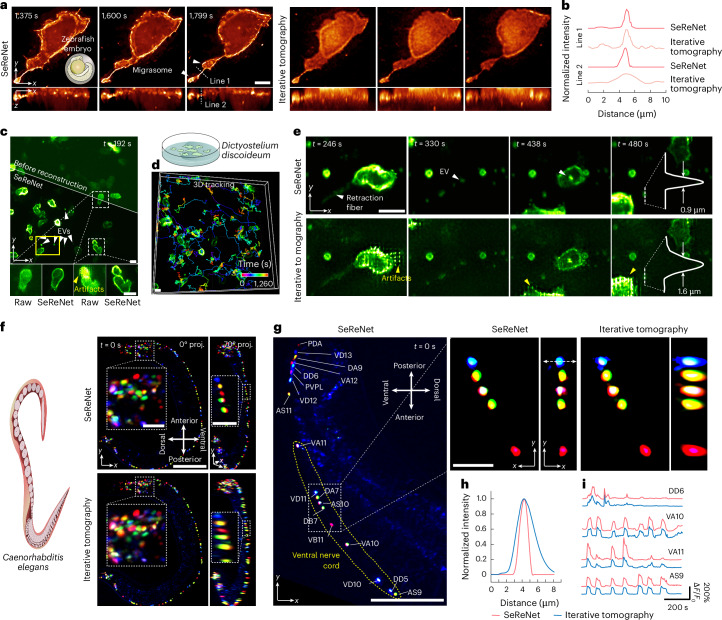
Experimental comparisons of SeReNet and other SOTA methods in diverse living organisms. **a**, Orthogonal maximum intensity projections (MIPs) showing the process of migrasome formation in a zebrafish embryo, obtained by SeReNet and iterative tomography. **b**, Normalized intensity profiles along the two lines marked in **a**. **c**, Center view and MIP obtained by SeReNet of membrane-labeled *D. discoideum* at *t* = 192 s, with white arrows indicating produced EVs and yellow arrows pointing to motion artifacts. The amoebas were cultured in a dish, as shown in the cartoon. **d**, Tracking of *D. discoideum* based on SeReNet results. The tracking traces were obtained through Imaris 9.0.1 software, with an overall tracking time of 1,260 s. A total of 49 cells were tracked with temporal-coding trajectory. The colors reflect different time points. **e**, Enlarged MIPs showing EV generation from *D. discoideum* at different stamps, comparing different methods. Profiles across an EV are compared, and yellow arrows indicate motion artifacts. **f**, Dual-directional MIPs and enlarged regions of an entire NeuroPAL worm (strain OH16230) obtained by SeReNet and iterative tomography. **g**, Orthogonal MIPs of neuron-labeled worm midbody by SeReNet, with enlarged regions showing comparisons between different methods. The identity is marked on the side of each neuron. **h**, Normalized intensity profiles along the marked dashed lines in **g**. **i**, Temporal traces (Δ*F*/*F*_0_) of GCaMP6s transients in four neurons, extracted from results of different methods. All networks were trained on the synthetic bubtub dataset, and the SeReNet here was the axially improved version. Scale bars, 10 μm (**a**,**c**–**e**, enlarged views in **f**,**g**), 50 μm (**f**,**g**, original views). Source data

First, we observed a membrane-labeled zebrafish embryo in vivo to investigate SeReNet’s applicability in developmental biology. Migrasomes, reported to be involved in organ morphogenesis^33^, require high resolution for detection owing to their small size. We showed the formation process of two migrasomes from an embryonic cell in different ways (Fig. 3a). Physics-inspired SeReNet could detect the subcellular details with their native morphologies (Fig. 3b and Supplementary Video 1). In addition, we demonstrated that SeReNet, even when trained with different datasets, exhibited stable performance (Supplementary Fig. 19).

In addition to migrasomes, extracellular vesicles (EVs) play an important role in cellular interactions in multicellular systems^34^. We imaged *D. discoideum*, which was membrane-labeled and cultured in a Petri dish. It is sensitive to photodamage, migrates fast and causes motion artifacts (part I of Supplementary Video 2). SeReNet corrected these artifacts, enabling high-fidelity reconstructions that allowed us to track the free-moving trajectories of *D. discoideum* (Fig. 3c,d). EVs and retraction fibers were visualized with narrower intensity profiles than those obtained using previous methods (Fig. 3e). During observation, we noted that large amounts of EVs were produced from elongated retraction fibers. Subsequently, an EV generated by one *D. discoideum* was picked up by another (part II of Supplementary Video 2). The phenomenon is similar to those of migrasomes in zebrafish embryos^33^ and living cells^35^, warranting further exploration.

*C.* *elegans* is another commonly used animal model, widely studied in developmental biology^36^ and neuroscience^9^. To show SeReNet’s application in large-scale neural imaging, we observed the whole body of a young NeuroPAL^37^ transgenic *C. elegans* worm (strain OH16230) with GCaMP6s indicators and multi-color neural identities. Each neuron was uniquely identified by its color and position (Fig. 3f and Supplementary Video 3). NeuroPAL localized fluorophore expression to cell nuclei, which were densely packed in 3D space and clearly identified by SeReNet with subcellular resolution. SeReNet also resolved four neurons in the ventral nerve cord, which are neatly and tightly packed in the midbody (Fig. 3g,h). The temporal traces of neurons showed enhanced SNR with our method (Fig. 3i).

With improvements in processing speed, resolution, robustness and versatility, synergizing SeReNet and sLFM presents a promising tool for subcellular observations both in vivo and ex vivo.

### High-fidelity investigation of subcellular dynamics in mice

Next, we used SeReNet to study liver injury in mice, highlighting its unique advantages in facilitating such applications. Mammalian liver injury is a systemic process involving multiple immune cells and organelles, and has become a global health concern^38–41^. Understanding the complex immune physio-pathogenesis at the cellular and subcellular levels is crucial for developing effective therapies. This not only requires an imaging system capable of high-speed 3D imaging in vivo, but also requires an algorithm for rapid, robust and high-fidelity reconstructions. Additionally, the liver’s dynamic environment, with constant blood flow and tissue movement, typically complicates imaging. However, SeReNet’s advanced noise reduction, aberration robustness and motion-correction capabilities ensured that the resulting images were of high quality.

**Fig. 4:**
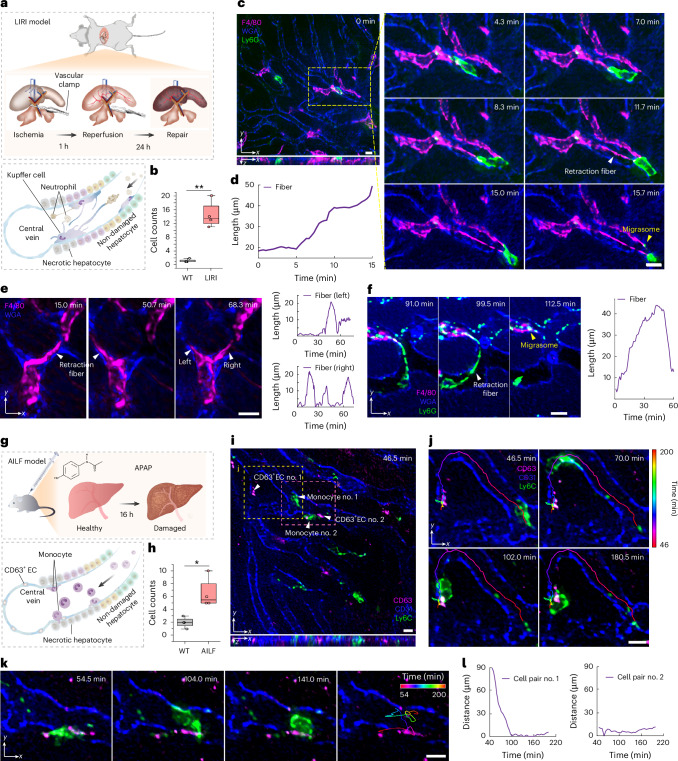
High-fidelity long-term imaging by SeReNet in mice with liver injury. **a**, Illustrations of the LIRI model. Mice were anesthetized and subjected to hepatic ischemia and reperfusion. After 24 h, liver regeneration was initiated. **b**, Boxplot showing the neutrophil counts in mouse livers without and with LIRI. *n* = 4 regions. *P* = 5.89 × 10^−3^. **c**, Orthogonal MIPs by SeReNet showing neutrophils (Ly6G, green) and KCs (F4/80, magenta) in the vessels (WGA, blue) of living mouse livers following LIRI, with enlarged MIPs showing the interactions between neutrophils and KCs. Arrows indicate the image blur and artifacts. **d**, Retraction fiber length of the KC over 15 min, showing the elongation process. **e**, MIPs showing that a KC stretched out a retraction fiber to touch another KC. The lengths of the two retraction fibers over 75 min are plotted. **f**, MIPs showing that neutrophils generated a long retraction fiber and produced a migrasome that was delivered into a KC. The retraction fiber length over 60 min is plotted. **g**, Illustrations of the AILF model. Mice were given an i.p. injection of APAP (600 mg kg^–1^) for 16 h to induce a proinflammatory phenotype. **h**, Boxplot showing the count of CD63^+^ ECs in mouse livers without and with AILF. *n* = 4 regions. *P* = 1.17×10^−2^. **i**, MIPs obtained by SeReNet of monocytes (Ly6C, green) and CD63^+^ ECs (CD63, magenta) in the vessels (WGA, blue) of livers of living mice with AILF. Arrows indicate the image blur and artifacts. **j**,**k**, Enlarged MIPs of two regions demonstrate the proximity process (**j**) and adhesion process (**k**) between monocytes and CD63^+^ ECs. The color-coded trajectories of two monocytes are overlaid. **l**, Centroidal distances between the monocytes and CD63^+^ ECs over time. In boxplots: center line, median; box limits, lower and upper quartiles; whiskers, 0th–100th percentiles. Asterisks represent significance levels tested with two-sided paired *t*-test. Significance at *P* < 0.05. *P < 0.05; **P < 1 × 10^−2^. SeReNet was trained on the synthetic bubtub dataset. Scale bars, 10 μm (**c**,**e**,**f**,**i**–**k**). Source data

We first established a liver ischemia–reperfusion injury (LIRI) model in wild-type mice (Fig. 4a and Methods). During recovery and liver regeneration after LIRI, Kupffer cells (KCs) and neutrophils play significant roles in tissue repair, but their specific mechanisms remain to be explored at the subcellular scale^42,43^. We injected antibodies and dyes intravenously (i.v.) into injured mice to label KCs, neutrophils and vessels, and the mouse livers were imaged during recovery (24 h post-LIRI). Compared with wild-type mice, in mice with LIRI, the number of neutrophils increased substantially, revealing various interactions between KCs and neutrophils (Fig. 4b and Supplementary Fig. 20). Notably, a neutrophil migrating in the vessels generated migrasomes inside a KC (Fig. 4c,d). As the neutrophil moved away, it was pulled by a thin retraction fiber extending from the KC for further interaction (part I of Supplementary Video 4). Thanks to SeReNet’s broad generalization capabilities, various subcellular structures during the bioprocess could be clearly distinguished without severe blurring or artifacts. We also observed KCs contacting each other through elongation and contraction of retraction fibers (Fig. 4e and part II of Supplementary Video 4), and a neutrophil producing a migrasome and delivering it into a KC by generating a long retraction fiber (Fig. 4f). These observations suggest that signals might be delivered between multiple immune cells through contact-generated migrasomes and intercellular pulling, facilitating innate immune system repair in mammals. Targeting these processes of organelle formation and intercellular pulling could offer potential therapies for LIRI.

Another common liver disease is drug-induced liver injury (DILI), characterized by the activation of endothelial cells (ECs) into a proinflammatory phenotype (Fig. 4g). This leads to an increase in CD63^+^ ECs with heightened adherent functions in the liver^44,45^ (Fig. 4h and Supplementary Fig. 21a,b). The dynamic interactions of CD63-marker-labeled ECs with immune cells, given their small size, require high spatiotemporal resolution to observe across an extended period for pathological state changes (Supplementary Figs. 21c and 22). We established the acetaminophen (APAP)-induced liver failure (AILF) model by injecting 600 mg kg^–1^ body weight of APAP intraperitoneally (i.p.) into wild-type mice 16 h before imaging (Fig. 4g,h and Methods). Monocytes, CD63^+^ ECs and vessels were then labeled through i.v. injection. SeReNet exhibited broad generalization, capturing intricate immune phenomena with improved fidelity and reduced artifacts across a long term, which fully exploits the low phototoxicity of sLFM. We observed CD63^+^ ECs recruiting monocytes from a distance using SeReNet-assisted sLFM (Fig. 4i,j and Supplementary Video 5). An hour-long observation revealed monocytes migrating in the vessels, adhering to CD63^+^ ECs and moving back and forth around them (Fig. 4k). The curves of intercellular distances verified these processes (Fig. 4l). Considering that recruitment and adhesion are common modes of intercellular communications, there could be signaling between endothelial cells and immune cells that exacerbates liver injury in mammals^44,46,47^. Targeting CD63^+^ ECs could provide new insights for developing effective therapies for drug-induced liver failure. Overall, SeReNet with sLFM reveals diverse intercellular interactions during liver injury over an extended period.

### Two-day-long continuous 3D imaging of inflammatory response

We further evaluated SeReNet’s capabilities by investigating the immune response after injury in zebrafish larvae, involving the active participation and migration of endogenous immune cells toward target tissues^48,49^. This process usually spans from seconds to several days, necessitating hundreds of thousands of frames for comprehensive analysis owing to the unpredictable behavior of cells. However, the massive quantity of data generated imposes a heavy computing burden on previous reconstruction algorithms, limiting the practical observation duration.

**Fig. 5:**
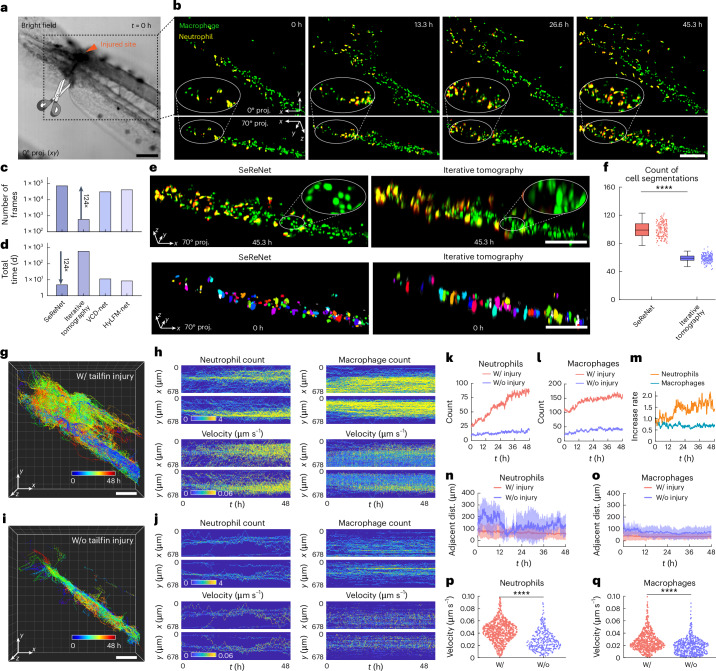
Rapid high-resolution reconstruction of 2-day-long monitoring of inflammatory dynamics in zebrafish with more than 345,600 volumes. **a**, Bright-field image depicting the zebrafish tailfin injury. **b**, Dual-directional MIPs of neutrophils (yellow) and macrophages (green) migrating in the vasculature, obtained by SeReNet. **c**,**d**, Comparison of volume numbers reconstructed within 48 h (**c**) and total processing time for the whole sequence (**d**). **e**, Shown are 70° MIPs comparing different reconstruction methods. Illustrative cell segmentations using CellPose are plotted below. **f**, Boxplot showing counts of cell segmentations. *n* = 201 images at different times. *P* = 1.07 × 10^−151^. **g**, Tracked traces of cells by SeReNet during inflammatory process. All cells (*n* = 1,531) were counted. **h**, Maps of average local cell densities (left) and velocities (right) along the *x* and *y* directions over time in injured zebrafish. **i**, Tracked traces of cells by SeReNet in non-injured zebrafish. All cells (*n* = 806) were counted. **j**, Maps of average local cell densities (left) and velocities (right) along the *x* and *y* directions over time in non-injured zebrafish. **k**,**l**, Curves of neutrophil (**k**) and macrophage (**l**) flow quantities over time. **m**, Increased rate in cell quantities for neutrophils and macrophages, calculated as the ratio of the cell number in the injured state to the non-injured state; data were normalized to *t* = 0 h. **n**,**o**, Curves of adjacent distances between neutrophils (**n**) and macrophages (**o**) over time. The adjacent distance of each cell is defined as the average distance between the ten cells closest to it. Data are represented as mean ± s.d. **p**,**q**, Violin plot of mean velocities of neutrophils (**p**) and macrophages (**q**). Mean velocity (μm s^–1^) was calculated for individual tracks by linear regression of trajectory lengths versus time. *n* represents the cell number. *n* = 828 (neutrophil, injured), *n* = 284 (neutrophil, non-injured), *n* = 703 (macrophage, injured), *n* = 522 (macrophage, non-injured). *P* = 2.30 × 10^−154^, 2.13 × 10^−19^. The boxplot formats: center line, median; box limits, lower and upper quartiles; whiskers, 1.5 × interquartile range. Asterisks represent significance levels tested with two-sided paired *t*-test; significance was set at *P* < 0.05. ****P < 1 × 10^−4^. SeReNet was the axially improved version and was trained on synthetic bubtub dataset. Scale bars, 100 μm (**a**,**b**,**e**,**g**,**i**). Source data

To demonstrate SeReNet’s advantage in this scenario, we utilized xenograft zebrafish larvae (*Tg(coro1a:EGFP;lyz:DsRed2)*) genetically expressing neutrophils and macrophages for imaging. The distal tips of the tailfin were carefully incised to generate an injury without amputation (Fig. 5a). With its unprecedented processing throughput, SeReNet with sLFM enabled day-long imaging and real-time reconstruction of multiple migrations of immune cells in the injured tails of living zebrafish larvae (Fig. 5b and part I of Supplementary Video 6). The larvae were continuously observed with dual colors at 1 volume per second, spanning 48 h and 345,600 time points (172,800 volumes for each channel). SeReNet took approximately 5 days on an NVIDIA A100-SXM4 GPU to handle this large dataset, producing a detailed, dynamic picture of immune cells actively migrating toward the injured site over the 2 days (Fig. 5c,d). By contrast, using deconvolution-based iterative tomography within the same time and computing resources, only about 1% of frames could be reconstructed. Reconstructing the whole sequence with iterative tomography would require several years, which is impractical for practical applications with downstream inflammatory analysis. Beyond the enhancement in efficiency, SeReNet also exhibits improved fidelity with higher resolution and more clearly distinguished cells compared with previous methods (Fig. 5e,f).

After acquiring this massive dataset, we quantified immune infiltration over 2 days. We tracked cell cohorts to characterize the migration of both neutrophils and macrophages toward the lesion, using the volumes obtained by SeReNet (Fig. 5g–j). There was a noticeable increase in cell number, velocity and migration after injury (Fig. 5h,j and part II of Supplementary Video 6). The phenomenon illustrates the pro-inflammatory property of immune cells in vivo. By monitoring cell densities, we observed a rapid accumulation of neutrophils and macrophages after injury (Fig. 5k,l). Neutrophils, in particular, showed more significant changes before and after injury, with a higher increase rate in quantity within 48 h (Fig. 5m). Quantitative analysis revealed that immune cells home in on sites of injury, as indicated by increased number and reduced intercellular distances (Fig. 5n,o). During this phase, a surge in migration speed occurs, while immune cells maintained a steady rhythm of movement in the non-injured zebrafish (Fig. 5p,q). Collectively, our efficient framework documented a continuous 48-h inflammatory response following injury, capturing intricate details of immune-cell dynamics. These lengthy, high-resolution observations underscore the potential of SeReNet in long-term, highly dynamic intravital biological experiments, extending its practicality significantly.

## Discussion

In this paper, we introduce SeReNet, a physics-driven self-supervised network for 3D reconstruction of LFM and its variants. SeReNet features a 700-fold increase in processing speed, more uniform resolution, and better sectioning ability compared with iterative tomography. It also has a twofold resolution enhancement, better generalization and greater robustness to noise, sample motion and aberrations compared with previous supervised networks. The ultrahigh processing throughput of SeReNet enables day-long recordings of up to 345,600 volumes, allowing for thorough analysis of sophisticated subcellular dynamics across multiple species. Its broad generalization capability ensures that pretrained SeReNet can be stably and robustly applied to data from the same sLFM system without requiring further parameter tuning. We have also demonstrated the effectiveness of SeReNet on different configurations of the system, including magnification, numerical aperture, angular resolution and scanning number (Supplementary Figs. 10 and 23).

Inspired by NeRFs^30^, which generate 3D representations of scenes from 2D images, SeReNet exhibits notable differences compared with traditional NeRFs. First, traditional NeRF methods are primarily designed for natural scene images under the assumption of straight-line ray propagation and do not incorporate physical priors such as wave-based PSF constraints and preDAO. By contrast, SeReNet is specifically designed for microscopy, in which the incorporation of wave-optics PSF priors is critical to maintain high resolution and fidelity near diffraction limit. Second, traditional NeRF-based methods are optimized directly on the specific sample to be reconstructed, leading to a lack of generalization. SeReNet better integrates the PSF physical priors through its elaborate network modules (Methods), effectively filtering out information not captured by the imaging system. As a result, SeReNet has superior generalization, a crucial capability in microscopy, since ground-truth images are often lacking. Third, after training, SeReNet achieves fast inference speeds at the millisecond level, whereas traditional NeRF-based methods require additional training for diverse structures, limiting their practicality for processing large-scale time-lapse data.

Notably, the proposed SeReNet and VsLFM^21^ belong to two individual processes within the processing pipeline of sLFM (Supplementary Fig. 24). VsLFM aims to replace the physical scanning process when the sample is highly dynamic, which can address the motion artifacts during the scanning process; however, a 3D reconstruction process is still required. Although the iterative tomography algorithm in VsLFM shows good performance and high generalization for 3D reconstruction, the reconstruction speed of iterative tomography is very slow, precluding its practical applications during daily use. SeReNet addresses this problem with even faster processing speed and improved generalization compared with previous supervised neural networks for LFM reconstruction. Our results show that SeReNet is also compatible with VsLFM^21^ (Supplementary Fig. 25). In addition, because SeReNet mainly relies on spatial-angular constraints, it could be well suited for other multi-view computational imaging systems, such as two-photon synthetic aperture microscopy^50^ (Supplementary Fig. 26). Future applications could extend to other setups such as Fourier LFM^11^, Fourier ptychographic microscopy^51^, confocal sLFM^52^ and meta-imaging sensors^53^, all of which would benefit from SeReNet’s accelerated reconstruction speeds.

The current SeReNet has several limitations. First, although SeReNet leverages physical priors effectively, its performance depends heavily on the accuracy of the PSFs. Inaccurate PSFs can lead to degradation in performance, depending on the level of optical aberrations. When imaging with severe aberrations, relying on an ideal PSF priors could lead to reduced performance. Although preDAO can be used in this process, considering aberrations during PSF generation in the network training process could further enhance the performance. Second, the current version of SeReNet uses only 195,000 network parameters to reduce the computational cost for high-throughput data. In the future, we can also consider scaling up with stronger computational resources to achieve better resolution. Optimizing the angle-selection strategy in sLFM could also accelerate the training process^54^. Third, the axially improved SeReNet mitigates the missing-cone problem, but there is a slight degradation in generalization. Incorporating a larger-scale dataset with real samples to construct a foundation model could further increase the generalization for axial improved SeReNet.

The practical limitations of computational imaging lie in processing speed and fidelity. Traditional deep-learning methods excel in speed, but their fidelity and generalization ability are constrained. SeReNet fills this niche, offering great promise for shedding light on various biological mysteries in cell biology, neuroscience, immunology and medicine. We believe that implementing SeReNet and leveraging its advantages, together with the great performance of computational imaging and mesoscopes, will facilitate large-scale processing and analysis of intercellular interactions at the organ level^55^, paving the way for new discoveries and therapeutic approaches.

## Methods

### Experimental setup

SeReNet’s performance was evaluated in multiple sLFM and LFM systems. Most experiments were performed on an inverted sLFM system we reported previously^15,22^, configured with a ×63, 1.4-numerical-aperture (NA) oil-immersion objective (Zeiss Plan-Apochromat ×63/1.4 Oil M27), a sCMOS camera (Andor Zyla 4.2 Plus) and a commercial microlens array (MLA) (RPC Photonics MLA-S100-f21) featuring a pitch size of 100 μm and focal length of 2,100 μm. Each microlens covered exactly 13 × 13 angular pixels. We also constructed other sLFM systems to demonstrate the versatility of SeReNet. The tumor organoid experiments (Supplementary Fig. 23a) were performed on an sLFM system that was equipped with an Olympus UPLFLN20XP objective (×20/0.5-NA, air-immersion) and a customized MLA, with a pitch size of 97.5 μm and focal length of 1,950 μm. The jellyfish experiments (Supplementary Fig. 23b) were performed on an upright system with an Olympus MVPLAPO objective (×2/0.5-NA, air-immersion) and a customized MLA with a pitch size of 69 μm and focal length of 800 μm. The brain slice experiments (Supplementary Fig. 23c) were performed on an upright sLFM system with an Olympus XLPLN25XWMP2 objective (×25/1.05-NA, water-immersion) and a customized MLA with a pitch size of 136.5 μm and focal length of 2,800 μm, to cover more angular views. To optimize the photon efficiency, the wide-field illumination was used to excite the sample. During sLFM acquisition, a 2D galvo was synchronized to periodically scan the light field at the image plane before the light passed through the MLA. In LFM imaging, the piezo tip/tilt platform was set to stationary in the offset position.

Detailed parameters for all the fluorescence experiments, including the fluorescent label, laser wavelength, excitation power, exposure time, imaging speed, objective, angular resolution and scanning number, are provided in Supplementary Table 3.

### Pipeline of SeReNet

As described in the previous study^15^, the scanning light-field image (*M*) is defined on a 4D phase space containing an angular coordinate (${\bf{u}}=({u}_{1},{u}_{2})$) and spatial coordinate (${\bf{x}}=({x}_{1},{x}_{2})$). The specific spatial-angular measurements of sLFM can be represented as $M({\bf{x}},{{\bf{u}}}_{j})$, where *j* corresponds to the index of the angular component **u**_*j*_. The corresponding PSF of the angular component **u**_*j*_ is represented by $H({\bf{x}},z,{{\bf{u}}}_{j})$, where *z* denotes the axial coordinate. Iterative tomography is intended to reduce the error map between the *j*th angular measurement ($M({\bf{x}},{{\bf{u}}}_{j})$) and the forward projection of the iteratively estimated volume (${g}^{k}({\bf{x}},z)$) along **u**_*j*_. The projection can be integrated as:

$${P}^{k}({\bf{x}},{{\bf{u}}}_{j})={\int }_{z}{g}^{k}({\bf{x}},z)\ast H({\bf{x}},z,{{\bf{u}}}_{j})dz,$$

where * is the 2D convolution on the **x** dimension, *dz* corresponds to the integration variable in the axial domain and *k* is the current iteration number. The iterative tomography can be viewed as an unsupervised optimization method, but its iterative process is very time-consuming. To accelerate the reconstruction, we propose that SeReNet should learn a direct mapping from spatial-angular measurements ($M({\bf{x}},{\bf{u}})$) to a high-resolution network estimation ($\tilde{g}({\bf{x}},z)$) by reducing the loss between $M({\bf{x}},{\bf{u}})$ and the forward projections of $\tilde{g}({\bf{x}},z)$ during the self-supervised network training process. For this purpose, SeReNet is designed with 195,000 network parameters, embedded in three main modules: the depth-decomposition module, the deblurring and fusion module and the self-supervised module (Supplementary Fig. 2 and Supplementary Table 1).

First, the depth-decomposition module extracts 3D information from spatial-angular components. Motivated by digital refocusing used in LFM^56^, we notice the angular disparities imply the depth information of samples, which enables the 3D reconstruction to converge more rapidly and accurately (Supplementary Fig. 6). A traditional digital refocusing algorithm^57^ can be separated into two steps: multiple spatial-angular views are first shifted according to the corresponding angle and required depth, to obtain multiple angular volumes (${g}_{a}({\bf{x}},{\bf{u}},z)$), and different angular volumes are then integrated into a refocused volume (${g}_{r}({\bf{x}},z)$). Because the integration operation is compressive and irreversible, SeReNet adopts only the first step of digital refocusing through depth-coded convolution series, as formularized by:

$${g}_{a}({\bf{x}},{\bf{u}},z)=M({\bf{x}}-\alpha z{\bf{u}},{\bf{u}})=M({\bf{x}},{\bf{u}})\ast \delta ({\bf{x}}-\alpha z{\bf{u}}),$$

where $\delta (\cdot )$ is impulse function, *α* is a scaling factor and *α***u** is the slope map of multi-angle PSFs. $\delta ({\bf{x}}-\alpha z{\bf{u}})$ is also regarded as geometrical-simplified PSFs, which can achieve axial sectioning roughly (Supplementary Fig. 1).

Second, the deblurring and fusion module is designed for incoherent aperture synthesis and high-fidelity 3D reconstruction. The angular volumes are rearranged into a 4D tensor, where the angle information is packed into the channel dimension. The module comprises nine 3D convolutional layers and three linear interpolation layers, containing most of SeReNet’s network parameters. This design enables more-uniform spatial resolution and better axial performance (Supplementary Figs. 8 and 9). Detailed network parameters are provided in Supplementary Fig. 2 and Supplementary Table 1. By enabling rich interaction and fusion of angular features, the module effectively captures physical correlations and progressively converges to a 3D estimation. The deblurring and fusion module can be formularized as:

$$\tilde{g}({\bf{x}},z)={f}_{\theta }({g}_{a}({\bf{x}},{\bf{u}},z)),$$

where *θ* represents the network parameters that need to be optimized.

Third, the self-supervised module guides the learning process rationally by imposing physical priors. Forward projections are performed on the estimated volume ($\tilde{g}({\bf{x}},z)$) along 21 randomly selected angles each time, using the 4D wave-optics angular PSFs of sLFM. All angular components are traversed during the whole training iterations and are balanced by the learning rate. After scaling to the same pixel size, the distance between the spatial-angular projections and corresponding measurements ($M({\bf{x}},{\bf{u}})$) are used as loss function for self-supervised training, which can be represented by:

$$\sum _{j}\rm{los{s}}_{\rm{NLL-MPG}}({\it{\tilde{P}}}({\bf{x}},{{\bf{u}}}_{\textit{j}}),\textit{M}({\bf{x}},{{\bf{u}}}_{\textit{j}})),$$

where $\tilde{P}({\bf{x}},{{\bf{u}}}_{j})={\int }_{z}\tilde{g}({\bf{x}},z)\ast H({\bf{x}},z,{{\bf{u}}}_{j})dz$ represents the forward projections of the estimated volume $\tilde{g}({\bf{x}},z)$. The loss calculation is derived in the next section. The selective angle strategy in SeReNet resembles the ptychographic approach in iterative tomography^15,17^ or axial slicing in HyLFM-Net^26^, but operates in frequency domain. This promotes efficient convergence and conserves GPU memory. Of note, the read-in and processing of the wave-optics PSFs is time-consuming. But fortunately, the self-supervised module is involved only in the training phase and does not lower the inference speed.

Our sLFM system acquired data with 3 × 3 scanning times and 13 × 13 angles. Forty-nine spatial-angular views near the microlens center were extracted as network input, which is consistent with iterative tomography in sLFM^15^. For network training, we first cropped the data into patches measuring 49 × 99 × 99 pixels (angle × height × width), which were then unrolled into angularly refocused volumes of size 49 × 101 × 99 × 99 pixels (angle × depth × height × width) in the depth-decomposition module. Next, the 3D estimation output from the deblurring and fusion module was of size 101 × 429 × 429 pixels (depth × height × width). Finally, the 3D output was used to generate multi-angle projections, each of size 429 × 429 pixels (height × width). SeReNet was implemented on the PyTorch platform with a single NVIDIA A100-SXM4 GPU. The training for 800 epochs on a typical dataset (roughly 90 sLFM data) took approximately 18 h.

During network inference, SeReNet took approximately 50 ms for a volume of size 101 × 429 × 429 pixels (depth × height × width). For reconstruction of data with more pixels, partially overlapping patches were processed separately and stitched using sigmoid-based volume fusion^58^. Source code for SeReNet has been released for broad open-source applications, maximizing its accessibility.

### Derivation of NLL-MPG loss function

For a given ground-truth volume ($g({\bf{x}},z)$), the ideal imaging process of sLFM is represented as:

$$M{\prime} ({\bf{x}},{{\bf{u}}}_{j})={\int }_{z}g({\bf{x}},z)\ast H({\bf{x}},z,{{\bf{u}}}_{j})dz,$$

where $M{\prime} ({\bf{x}},{{\bf{u}}}_{j})$ denotes spatial-angular measurements without noise. However, in practical applications, various types of noise are unavoidable. Among them, readout uncertainty follows a Gaussian distribution, whereas photon shot noise follows a Poisson distribution^59^. Therefore, the actual observation ($M({\bf{x}},{{\bf{u}}}_{j})$) can be better modeled as following a mixed Poisson–-Gaussian (MPG) distribution, which can be represented as:

$$M({\bf{x}},{{\bf{u}}}_{j})={\rm{Poisson}}(M{\prime} ({\bf{x}},{{\bf{u}}}_{j}))+n,$$

where $\rm{Poisson}(\cdot )$ is the Poisson process, *τ* is the number of photons arriving per unit time, and $n\sim N(\mu ,{\sigma }^{2})$ is the noise that follows a Gaussian distribution with a mean of *μ* and a variance of *σ*^2^. In SeReNet’s training process, the forward projections ($\tilde{P}({\bf{x}},{{\bf{u}}}_{j})$) in the self-supervised module can be regarded as the ideal spatial-angular measurements for the estimation $\tilde{g}({\bf{x}},z)$. If the algorithm converges, $\tilde{P}({\bf{x}},{{\bf{u}}}_{j})$ will be equivalent to $M{\prime} ({\bf{x}},{{\bf{u}}}_{j})$, and the noise model can be expressed as:

$$M({\bf{x}},{{\bf{u}}}_{j})={\rm{Poisson}}(\tilde{P}({\bf{x}},{{\bf{u}}}_{j}))+n.$$

*p*(*M*) is the probability density function of $M({\bf{x}},{{\bf{u}}}_{j})$, and the Poisson and Gaussian components are independent in the MPG noise model. The likelihood of *M* given $\tilde{P}$, *μ*, *σ*^2^, is the joint probability function of the MPG noise, derived as:

$$p(M|\tilde{P},\mu ,\sigma )=\prod _{{\bf{x}},{\bf{u}}}\mathop{\sum }\limits_{\tau =0}^{+\infty }\frac{{\tilde{P}}^{\tau }\exp (-\tilde{P})}{\tau !}\frac{1}{\sqrt{2\pi }\sigma }\exp \left(-\frac{{(M-\mu -\tau )}^{2}}{2{\sigma }^{2}}\right).$$

Following the maximum likelihood principle, the loss function based on negative log-likelihood can be expressed as:

$$\begin{array}{c}\mathrm{los{s}_{NLL-MPG}}=-\!\log(\,p(M|\tilde{P},\mu ,\sigma ))\\ =-\!\log\left(\prod _{{\bf{x}},{\bf{u}}}\mathop{\sum }\limits_{\tau =0}^{+\infty }\displaystyle\frac{{\tilde{P}}^{\tau }\exp (-\tilde{P})}{\tau !}\frac{1}{\sqrt{2\pi }\sigma }\exp \left(-\frac{{(M-\mu -\tau )}^{2}}{2{\sigma }^{2}}\right)\right).\end{array}$$

For convenient mathematical manipulation, Stirling’s approximation is used to deal with the factorial operator. The simplified loss function can be formularized as:

$$\mathrm{los{s}_{NLL-MPG}}=-\!\log \left(\prod _{{\bf{x}},{\bf{u}}}\mathop{\sum }\limits_{\tau =0}^{+\infty }\frac{{(\tilde{P}/\tau )}^{\tau }\exp (\tau -\tilde{P})}{\sqrt{2\pi \tau }}\frac{1}{\sqrt{2\pi }\sigma }\exp \left(-\frac{{(M-\mu -\tau )}^{2}}{2{\sigma }^{2}}\right)\right).$$

The Gaussian parameters *μ* and *σ* are camera-intrinsic properties, which are independent of the sample intensity but are influenced by the readout time. We characterized *μ* and *σ* given the specific exposure time, using the dark images without illumination provided, or the corner views of the LF measurement because they are also very dark. With the NLL-MPG loss, SeReNet can optimize the distance between probability distributions of signals instead of the absolute distance between gray values, to achieve considerable noise robustness in photon-limited conditions (Supplementary Figs. 11 and 12).

Next, we clarify the theoretical soundness of NLL-MPG loss. The NLL-MPG loss can guide the rationalized convergence of SeReNet, mirroring the principles of iterative tomography. Owing to the advanced manufacturing and cooling techniques for sCMOS, both the readout and dark noise are relatively low, whereas the photon shot noise plays the dominant role. Ignoring the effect of Gaussian noise, the measurements can be represented as:

$$M({\bf{x}},{{\bf{u}}}_{j})=\mathrm{Poisson}(\tilde{P}({\bf{x}},{{\bf{u}}}_{j})).$$

The neglection of the Gaussian term means that $\frac{\frac{1}{\sqrt{2\pi }\sigma }\exp (-\frac{{(M-\mu -\tau )}^{2}}{2{\sigma }^{2}})}{\mathop{\sum }\nolimits_{\tau =0}^{+\infty }\frac{1}{\sqrt{2\pi }\sigma }\exp (-\frac{{(M-\mu -\tau )}^{2}}{2{\sigma }^{2}})}$ should be a constant, where *μ* = $\sigma \to 0,$ and *τ* is equal to *M*. The loss function of SeReNet is correspondingly degenerated into:

$$\begin{array}{c}\mathrm{los{s}_{NLL-MPG}}=-\!\log (p(M|\tilde{P}))\\ =-\!\log \left(\prod _{{\bf{x}},{\bf{u}}}\displaystyle\frac{{\tilde{P}}^{M}\exp (-\tilde{P})}{M!}\right)\\ =\sum _{{\bf{x}},{\bf{u}}}P-M\,\log P+M!.\end{array}$$

The derivative of ${\rm{loss}}_{\rm{NLL-MPG}}$ with respect to $\tilde{g}({\bf{x}},z)$ is formularized as

$$\begin{array}{l}\displaystyle\frac{\mathrm{\partial los{s}_{NLL-MPG}}}{\partial \tilde{g}}=\\\qquad\displaystyle\frac{\partial \sum _{{\bf{x}},{\bf{u}}}{\int }_{z}\tilde{g}({\bf{x}},z)\ast H({\bf{x}},z,{{\bf{u}}}_{j})dz-M\,\log ({\int }_{z}\tilde{g}({\bf{x}},z)\ast H({\bf{x}},z,{{\bf{u}}}_{j})dz)}{\partial \tilde{g}}\\\qquad\displaystyle\frac{\partial \sum _{{\bf{x}},{\bf{u}}}{\int }_{z}\tilde{g}({\bf{x}},z) 1\ast H(-{\bf{x}},z,{{\bf{u}}}_{j})dz-M\,\log ({\int }_{z}\tilde{g}({\bf{x}},z)\ast H({\bf{x}},z,{{\bf{u}}}_{j})dz)}{\partial \tilde{g}}\\\qquad\qquad\qquad\qquad\quad\left(1-\frac{M({\bf{x}},{{\bf{u}}}_{j})}{{\int }_{z}\tilde{g}({\bf{x}},z)\ast H({\bf{x}},z,{{\bf{u}}}_{j})dz}\right)\ast H(-{\bf{x}},z,{{\bf{u}}}_{j}).\end{array}$$

We set it equal to 0, and replaced 1 with an updated item ($\frac{{g}^{k+1}({\bf{x}},z)}{{g}^{k}({\bf{x}},z)}$), leading to the formula of iterative tomography^15^:

$${g}^{k+1}({\bf{x}},z)\leftarrow {g}^{k}({\bf{x}},z)\left(\frac{M({\bf{x}},{{\bf{u}}}_{j})}{{g}^{k}({\bf{x}},z)\ast H(-{\bf{x}},z,{{\bf{u}}}_{j})}\ast H(-{\bf{x}},z,{{\bf{u}}}_{j})\right).$$

Therefore, the loss function based on the shot noise is theoretically consistent with iterative tomography. The NLL-MPG loss function, which accounts for both Gaussian and Poisson noises, represents a comprehensive upgrade of iterative tomography, making SeReNet more rationalizable and improving its convergence.

### Real-time TW-Net preprocessing for motion correction

In sLFM with 3 × 3 scanning, the high-resolution spatial-angular measurement ($M({\bf{x}},{\mathbf{u}},{t}_{0})$) at each time stamp *t*_0_ is composed of 9 low-resolution images (${M}_{L}({\bf{x}},{\mathbf{u}},t)$) at different galvo scanning positions, which can be represented as:

$$M({{\mathbf{x}}}+{\mathrm{pos}}(t),{{\mathbf{u}}},{t}_{0})={M}_{L}({{\mathbf{x}}},{{\mathbf{u}}},t),t \in [{t}_{0}-4,{t}_{0}+4].$$

pos(*t*) is the scanning position at the time *t*. Motion artifacts will appear if the actual spatial sampling positions of microlenses are shifted owing to the sample movements during nine-image acquisition. The previous time-weighted algorithm^15^ applies inverse distance weighting in the spatiotemporal domain, which can be represented as:

$$\begin{array}{c}{M}_{\mathrm{TW}}({{\mathrm{x}}},{{\mathrm{u}}},{t}_{0})={\mathrm{TW}}(M({{\mathrm{x}}},{{\mathrm{u}}},{t}_{0}),r)\\ ={\mathrm{rM}}({{\mathrm{x}}},{{\mathrm{u}}},{t}_{0})+(1-r)\mathop{\sum }\limits_{t=0}^{T}\displaystyle\frac{{r}^{|t-T/2|}}{\mathop{\sum }\nolimits_{tt=0}^{T}{r}^{|tt-T/2|}}{\mathrm{ShiftCubic}}({M}_{L}({{\mathrm{x}}},{{\mathrm{u}}},t),3).\end{array}$$

The time-weighted coefficient (*r*) ranges from zero to one, and in this case, the scanning period (*T*) is nine. $\mathrm{TW}(\cdot )$ is the time-weighted algorithm, and ${M}_{\mathrm{TW}}({\bf{x}},{\boldsymbol{\mathrm{u}}},{t}_{0})$ is the processed spatial-angular measurement. $\mathrm{ShiftCubic}()$ is a shift-interpolation function, which can be represented as:

$$\mathrm{ShiftCubic}({M}_{L}({\boldsymbol{\mathrm{x}}}+\mathrm{pos}(t),{\boldsymbol{\mathrm{u}}},t),3)=\mathrm{Cubic}({M}_{L}({\boldsymbol{\mathrm{x}}},{\boldsymbol{\mathrm{u}}},t),3).$$

$\mathrm{Cubic}(,3)$ is a threefold spatial interpolation function based on an extension of cubic spline interpolation. However, the global coefficient would cause resolution degradation owing to non-uniform motion property within the field of view^15,22^. The time-weighted algorithm is a low-pass filter to smooth the regions with motion artifacts. Regions without motion should not be processed. To address this, we developed a content-aware time-weighted network, termed TW-Net, for real-time non-uniform motion correction of sLFM data. The framework of TW-Net is based on a self-supervised learning framework, which can be represented as:

$$r({\boldsymbol{\mathrm{x}}})=\mathrm{TW-Net}(M({\boldsymbol{\mathrm{x}}},{\boldsymbol{\mathrm{u}}},{t}_{0})),$$

$${M}_{\mathrm{TW}}({\boldsymbol{\mathrm{x}}},{\boldsymbol{\mathrm{u}}},{t}_{0})=\mathrm{TW}(M({\boldsymbol{\mathrm{x}}},{\boldsymbol{\mathrm{u}}},{t}_{0}),r({\boldsymbol{\mathrm{x}}})).$$

*r*(**x**) is the spatially nonuniform time-weighted coefficients, and $\mathrm{TW-Net}(\cdot )$ is the TW-Net. A detailed workflow for TW-Net is provided in Supplementary Fig. 13a. TW-Net can find the optimal weighting map at every local region automatically by using MSE loss and a negative-log regularization to encourage the coefficient map to be as large as possible, to preserve high-resolution content. Unlike methods that only remove outliers within a local region, the network is better able to retrieve fidelity from dynamic samples across diverse datasets.

To train TW-Net, we first cropped single views of sLFM data into patches of size 1 × 153 × 153 pixels (angle × height × width), which were then processed by TW-Net to obtain a coefficient map of size 1 × 459 × 459 pixels. The time-weighted algorithm is applied with this map to get the final motion-corrected output. A total of 169 angles would be traversed one by one for sLFM data. TW-Net was implemented on the PyTorch platform with a single NVIDIA A100-SXM4 GPU. The training for 800 epochs on a typical dataset (roughly 90 sLFM samples) took approximately 3 h. During network inference, TW-Net took about 0.08 s to generate a time-weighted coefficient map with size of 169 × 459 × 459 pixels; the time-weighted algorithm took about 0.02 s. Finally, the spatial-angular images output from TV-Net were further reconstructed with SeReNet.

### preDAO processing for high-speed aberration correction

As described in a previous work^15^, different spatial-angular components of sLFM correspond to different sub-aperture regions. Aberrations due to refractive index inhomogeneities along the propagation path between the objective and the sample will deflect rays in different sub-aperture regions and shift their phases^60^. The correlation among angular measurements can therefore be exploited to infer aberrations, according to the translation-shifting property of the Fourier theorem. In the DAO algorithm^15^, aberration correction is performed digitally by shifting angular projections according to the estimated aberrations during the process of iterative tomography, which can be formularized as:

$$\mathrm{disp}^{k-1}({\bf{x}},{{\bf{u}}}_{j})=\mathrm{Cor{r}}_{\mathrm{ExDefoucs}}({P}^{k-1}({\bf{x}},{{\bf{u}}}_{j}),M({\bf{x}},{{\bf{u}}}_{j})),$$

$${\overline{P}^{k}}({\bf{x}},{{\bf{u}}}_{j})={P}^{k}({\bf{x}}+\mathrm{dis{p}}^{k-1}({\bf{x}},{{\bf{u}}}_{j}),{{\bf{u}}}_{j}).$$

$\mathrm{Cor{r}_{ExDefoucs}}(\cdot )$ represents 2D correlation with defocusing item excluded on dimension **x**, and $\mathrm{dis{p}}^{k}({\bf{x}},{{\bf{u}}}_{j})$ and ${\overline{P}^{k}}({\bf{x}},{{\bf{u}}}_{j})$ represent the disparity and corrected projection along the angle **u**_*j*_ for the *k*th iteration. We further developed a fast preDAO to correct the aberration in one step. In our preDAO, the aberration-induced disparities ($\mathrm{disp}({\bf{x}},{{\bf{u}}}_{j})$) between multiple angular measurements and the center view are estimated using 2D correlation with the defocusing component excluded, and are then corrected digitally by shifting angular measurements before reconstruction (Supplementary Fig. 14a). The calculation for preDAO can be expressed as:

$$\mathrm{disp}({\bf{x}},{{\bf{u}}}_{j})=\mathrm{Cor{r}_{ExDefoucs}}(M({\bf{x}},{{\bf{u}}}_{j}),M({\bf{x}},{{\bf{u}}}_{0})),$$

$$\overline{M}({\bf{x}},{{\bf{u}}}_{j})=M({\bf{x}}+\mathrm{disp}({\bf{x}},{{\bf{u}}}_{j}),{{\bf{u}}}_{j}).$$

$M({\bf{x}},{{\bf{u}}}_{0})$ represents the center-view measurement, and $\overline{M}({\bf{x}},{{\bf{u}}}_{j})$ represents multiple spatial-angular measurements after aberration correction by preDAO. The preDAO algorithm does not require iteration but still exhibits desirable accuracy in aberration estimation, nearly matching that of the DAO algorithm (Supplementary Fig. 14), and preDAO can be completed within 5 ms for one frame of the sLFM measurement (size of 49 × 99 × 99 pixels, angle × height × width).

### SeReNet fine-tuning for enhanced axial performance

To mitigate the intrinsic problem of ‘missing cone’^23^ in light-field imaging, we introduce an axial fine-tuning strategy aimed at leveraging the axial prior of volumetric data. During the SeReNet fine-tuning process, we increase the model capacity as well as the complexity by replacing the original last three layers of deblurring and fusion module with a fine-tuning module (based on U-net structure), as described in Supplementary Fig. 17 and Supplementary Table 1. The fine-tuning module involves two symmetric downsampling and upsampling layers with skip connections, which would not change the output feature size. The self-supervised module is not used in the fine-tuning stage.

When applying axially improved SeReNet, we first obtain a pretrained SeReNet using synthetic bubtub dataset. Then, we fix the pretrained model and optimize other parameters in the fine-tuning module. During the fine-tuning process, we use synthetic volumetric data as targets for supervision, which are also generated in the bubtub dataset (Supplementary Fig. 16a). The NLL-MPG loss function is also used with respect to 3D volumetric data. The ‘self-supervised pretraining + fine-tuning’ strategy is achieved only by updating the network parameters within the fine-tuning module while keeping the other parts of SeReNet to preserve physical priors. Therefore, axial performance could be improved without other sacrifices.

Axially improved SeReNet has approximately 214,700 parameters, just 10% more than that in original SeReNet. We trained the axially improved SeReNet with ~800 epochs, taking 8 h to achieve convergence. For network inference, the axially improved SeReNet took approximately 65 ms to reconstruct a volume of size 101 × 429 × 429 pixels (depth × height × width) on a single NVIDIA A100-SXM4 GPU.

### Calculation of processing throughput

For fluorescence imaging, the information content is commonly described by the number of optically resolvable spots within the 3D field of view, also defined as space-bandwidth product (SBP)^61^. Owing to the non-uniform spatial resolution of LFM or sLFM across the axial depths, the information content is calculated by the following formula:

$$\mathrm{Content}=\mathop{\sum}\limits_{p=1}^{N}16 \frac{{D}_{x}{D}_{y}{D}_{z}}{{r}_{xy,p}^{2}{r}_{z,p}}({\rm{bits}}).$$

The bit depth of each voxel is 16, *N* denotes the number of blocks in the axial direction, *D*_*x*_, *D*_*y*_ and *D*_*z*_ denote the spatial ranges along *x*, *y* and *z* directions (unit of μm) and *r*_*xy,p*_ and *r*_*z,p*_ represent the average lateral and axial resolutions in the *p*th block (unit of μm), as demonstrated in Supplementary Figs. 8 and 9. Then, the processing throughput is defined as the product of information content per volume and the number of volumes processed per second:

$$\mathrm{Throughput}=\mathop{\sum}\limits_{p=1}^{N}16\frac{{D}_{x}{D}_{y}{D}_{z}}{{r}_{xy,p}^{2}{r}_{z,p}}\left/{t}_{V}({\rm{bits}}\,{\rm{s}^{-1}}).\right.$$

*t*_*V*_ represents the average time to reconstruct one volume.

### Benchmarking with diverse algorithms

We compared the performance of SeReNet with SOTA algorithms, including iterative tomography^15^, RLN^27^, VCD-Net^25^, HyLFM-Net^26^ and DINER^30^. All methods were evaluated on 3 × 3 scanning light-field images for subsequent 3D reconstructions, unless stated otherwise. For iterative tomography, the reconstructions were performed as outlined in the original paper^15^. The processing time for a single volume (size of 101 × 429 × 429 pixels, depth × height ×width) was roughly 36 s on a single NVIDIA A100-SXM4 GPU.

The RLN, designed for a fixed imaging process with a spatially-uniform PSF, requires both input and output to be 3D volumes, preventing it from directly performing sLFM reconstruction. To adapt RLN for sLFM images, we implemented a preprocessing step. Spatial-angular views of sLFM were transformed into a volume through upsampling and digital refocusing^56^, creating the input for RLN. The corresponding high-resolution volume served as the target. Multiple spatial-angular views (size of 49 × 459 × 459 pixels, angle × height × width) were laterally upsampled into high-resolution ones (size of 49 × 1,989 × 1,989 pixels) using bicubic interpolation and were digitally refocused into a blurry 3D volume (size of 101 × 1,989 × 1,989 pixels, depth × height × width). We used the original network structure of RLN^27^ without any modifications. To facilitate use on our system, the TensorFlow version of the code was converted to PyTorch. Then, RLNs were trained with the blurry volumes as input and corresponding ground-truth volumes (size of 101 × 1,989 × 1,989 pixels) as the target data. For RLN training, the input and target were randomly cropped to 100 × 260 × 260 pixels. For network inference, partially overlapping patches of size 100 × 260 × 260 pixels were cropped with a 55-pixel overlap and were then processed by the networks, followed by sigmoid-based image fusion. We trained 800 epochs on a typical dataset (roughly 90 sLFM data samples) for RLN; the inference time for a single volume (size of 101 × 429 × 429 pixels) was roughly 220 ms. Both network training and inference were done on a single NVIDIA A100-SXM4 GPU.

The original VCD-Net and HyLFM-Net are designed for unscanned LFM data. We used the open-source codes of VCD-Net and HyLFM-Net to reconstruct the unscanned LFM data. To expand the applications to scanning light-field data, the network structures of VCD-Net and HyLFM-Net were slightly modified. The high-resolution spatial-angular images obtained through 3 × 3 scanning times were used as input; corresponding ground-truth volumes served as labels. In detail, our sLFM system acquired spatial-angular images with size of 169 × 459 × 459 pixels (angle × height × width) and obtained volumes of size 101 × 1,989 × 1,989 pixels (depth × height × width) after reconstruction. Thus, the input channel of VCD-Net and HyLFM-Net were adjusted to169 pixels, and the final output channel was set to 101. The original HyLFM-Net had an overall 4× scale magnification, and its end was appended by a 13/12× bicubic interpolation to match the volume size. Correspondingly, the original VCD-Net had four 2× subpixel upscaling layers, including a bicubic interpolation that was attached to the end of the network to match the volume size. Except for above adjusted parameters, we kept the modified networks as consistent as possible with that proposed in the original paper^25,26^. For network training, we randomly cropped small patches measuring 169 × 192 × 192 pixels as well as corresponding volume regions of 101 × 832 × 832 pixels, to create data pairs. Both VCD-Net and HyLFM-Net require around 800 epochs on a typical dataset (roughly 90 sLFM data, totaling 72,000 iterations) for training convergence. For network inference, partially overlapped patches of size 169 × 237 × 237 pixels were cropped and processed by the networks. The same sigmoid-based image fusion used in SeReNet was adopted to stitch the output sub-volumes. The entire training process of VCD-Net and HyLFM-Net took approximately 15 h, and the inference time for one volume (size of 101 × 429 × 429 pixels) took roughly 100 ms for VCD-Net and 65 ms for HyLFM-Net. Network training and inference were conducted on a single NVIDIA A100-SXM4 GPU. RLN, VCD-Net and HyLFM-Net are fully supervised and demand ground truth for network training. The training datasets are indicated in the figure legends.

For comparison with traditional NeRF, we used DINER^30^, a SOTA NeRF method, to reconstruct sLFM data. The directions of ray propagation were derived from wave-optics multi-angular PSFs. The input data, initially sized 49 × 273 × 273 pixels (angle × height × width), was first upsampled to 49 × 1,183 × 1,183 pixels before being processed by DINER. After 150 epochs, DINER produced an output volume of size 101 × 1,183 × 1,183 pixels (depth × height × width). The training time was approximately 1,200 s on a single NVIDIA A100-SXM4 GPU.

### Difference of model architectures between SeReNet and DINER

Both DINER and SeReNet are inspired by NeRFs, but differ in their model architectures. DINER primarily utilizes multi-layer perceptrons as its foundational architecture, whereas SeReNet is mainly based on convolutional neural networks. Specifically, DINER begins by randomly initializing three volumes (referred to as ‘hash-table’ length), with size of 3 × depth × height × width. These volumes are then mapped into the final prediction volume of depth × height × width, using a multi-layer perceptron. The number of neurons in each perceptron layer is configured as (3, 64, 64, 1). Both the multi-layer perceptron and the initialized three volumes are trainable parameters. To process the input LFM data, DINER is trained by updating all these parameters to generate the final prediction volume as the reconstruction result. Consequently, DINER must be retrained for each new dataset, which contributes to its poor generalization and slow processing speed. During the self-supervised training process, the predicted volume is projected into multi-angular images following the straight-line ray propagation. These projections are compared to the input LFM images using a loss function, which is then backpropagated to update the parameters. Multiple angles are iteratively used to calculate the loss function for each training iteration. After ~150 epochs, DINER will converge under the specific light field scene. The corresponding trainable parameter number of DINER is about 130 million. In the experimental setup of this study, the depth is set to 31 pixels, height and width are set to 1,183 pixels and the number of angles is set to 49. Under these conditions, the total number of trainable parameters in DINER is approximately 130 million.

By contrast, SeReNet employs a convolutional neural network rather than multi-layer perceptrons as its network architecture, which significantly reduces the number of parameters. As a result, SeReNet has approximately 600 times fewer parameters than DINER. Additionally, SeReNet introduces a depth-decomposition module before the convolutional neural network, which leverages a series of depth-coded convolutional operators to better extract depth information and enhance axial performance. In the self-supervised module, SeReNet incorporates wave-optics PSFs along multiple angles during the training process, rather than relying solely on straight-line ray propagation. This strategy more accurately reflects the image-formation process at the microscopy scale, facilitating improved network convergence. Throughout the training process, SeReNet updates only the parameters within the convolutional neural network without directly outputting a representation of a specific light field scene. Therefore, a well-trained SeReNet exhibits great generalization capabilities, enabling it to directly infer real biological data from a synthetic training set. This also achieves a high processing speed at millisecond level, which is unattainable with DINER.

### Definition of diffraction-limited resolution

The diffraction-limited resolution is defined based on Abbe limit and Rayleigh criterion, with the equation:

$$\begin{array}{c}\mathrm{resolutio{n}_{lateral}}=\displaystyle\frac{0.61 \lambda }{\mathrm{NA}},\\ \mathrm{resolutio{n}_{axial}}=\displaystyle\frac{n \lambda }{\mathrm{N{A}}^{2}},\end{array}$$

where NA is the NA of the objective lens, *n* is the refractive index and *λ* is the center wavelength of light (525 nm in this work). According to the equations, the optical diffraction limit primarily depends on NA and wavelength of the optical system, which is applicable to single-photon optical imaging systems.

### Bead preparation and resolution characterization

For the preparation of bead phantom, we first mixed 1,000 mg of pure agarose (Thermo Fisher UltraPure Low Melting Point Agarose, cat. no. 16520100) with 100 ml pure water, and heated the mixture to 80 °C, to generate 10 mg ml^–1^ diluted agarose. Then, we used 1 μl fluorescent beads with the diameter of 100 nm (Thermo Fisher TetraSpeck Microspheres, cat. no. T7279) that were diluted with 100 μl pure water. Next, we mixed 1 μl diluted fluorescent beads and 1 ml diluted agarose after the agarose cooled to around 40 °C, and transferred them into a 35-mm dish (Thermo Fisher Nunc glass bottom dish, cat. no. 150682). After about 30 min for solidification, the 3D uniformly distributed beads were created successfully. The environmental temperature was set at around 27 °C. During imaging, we used a ×63/1.4-NA oil-immersion objective to capture 50 groups of light field images and scanning light field images, each of them falling within a non-overlapping, randomly selected region. After acquisition, we applied reconstruction methods including iterative tomography, VCD-Net, HyLFM-Net and SeReNet to generate 3D stacks. The FWHM was utilized for quantitative resolution characterization. We measured the cross-sectional profiles laterally and axially with a Gaussian fit and calculated the FHWMs of these profiles as resolution indices. The calculation was conducted using a customized MATLAB script. We presented the data in the form of mean ± s.d. at different axial positions.

### L929 cell experiments

The L929 cells expressing *TOM20-GFP* and *Tspan4-mcherry* were generously provided by the laboratory of L. Yu at the School of Life Sciences at Tsinghua University. Subsequently, the cells were cultured on glass-bottom dishes (Thermo Fisher Nunc glass bottom dish, cat. no. 150682) to prepare them for imaging.

### Zebrafish experiments

To observe migrasome formation in zebrafish embryos, wild-type (Tuebingen strain) zebrafish embryos that were premaintained at 28.5 °C in Holtfreter’s solution (NaCl 59 mM, KCl 0.67 mM, CaCl_2_ 0.76 mM, NaHCO_3_ 2.4 mM), were injected with 300 pg of *PH-EGFP* mRNA at around the 16-cell stage (1.5 h postfertilization, hpf). At the 70% epiboly stage (8 hpf), we embedded the injected embryos into 1% low-melting-point agarose in a 35-mm dish (Thermo Fisher Nunc glass bottom dish, cat. no. 150682) for intravital imaging.

To demonstrate the day-long recordings of immune cell migrations in zebrafish larvae, *Tg(coro1a:EGFP;lyz:DsRed2)* transgenic zebrafish embryos were collected and kept at 28.5 °C in Holtfreter’s solution. At 3 days postfertilization, the tails of zebrafish larvae were pinched by tweezers, then mounted in 1% low-melting-point agarose for imaging. Meanwhile, larva tails without injury were also observed as a control group. During imaging, the environmental temperature was controlled at around 27 °C. The biological sex of the zebrafish used in the study is unknown.

### *Dictyostelium discoideum* cell culture

AX2 axenic strain cells were provided by the H. Cai laboratory (National Laboratory of Biomacromolecules, Institute of Biophysics, Chinese Academy of Sciences, China). The AX2 cell line, overexpressing myr–GFP, was cultured in HL5 medium (Formedium cat. no. HLF2), supplemented with antibiotics at 22 °C. The plasmids pDM323-poly was also provided by the H. Cai laboratory. The transformants overexpressing myr–GFP were maintained in HL5 medium containing G418 (20 μg ml^–1^) and hygromyosin (50 μg ml^–1^). The 35-mm dish (Thermo Fisher Nunc glass bottom dish, cat. no. 150682) was coated with poly-l-lysine (PLL, 1 μg ml^–1^) in BSS (10 mM NaCl, 10 mM KCl, 3 mM CaCl_2_) for 1 h. The cells were washed with BSS three times. Data were captured immediately after the cells were transferred onto the PLL surface. During imaging, the environmental temperature was kept at around 22 °C.

### *C. elegans* experiments

*C. elegans* strains were cultured on nematode growth medium plates seeded with OP50 *Escherichia coli* cells^62^. The *otIs670[NeuroPAL]; otIs672[panneuronal::GCaMP6s]* strains^37^ were provided by *Caenorhabditis* Genetics Center (CGC) and maintained at 20 °C. Young-adult hermaphrodites aged 3–4 days were anesthetized in M9 buffer containing 0.05 mM levamisole and then mounted on 2% agar pads. During sLFM imaging, the environmental temperature was kept at around 20 °C.

### Mouse experiments

Male wild-type C57BL/6J mice aged 6–8 weeks were purchased from Vital River Laboratory Animal Technology and housed with food and water available ad libitum under a 12 h light–dark cycle at around 22 °C with a relative air humidity of ~50%. To establish the LIRI model, wild-type mice were anesthetized with Avertin (350 mg kg^–1^ body weight, i.p.), and a midline laparotomy was performed. Then, 70% hepatic ischemia was accomplished using a microvascular clamp placed across the portal vein, hepatic artery and bile duct above the branching to the right lateral lobe. Blanching of the liver was considered a positive marker for hepatic ischemia. After 1 h of ischemia, reperfusion was initiated by removal of the clamp and confirmed by a return of the reddish color of the liver. Next, the abdominal incision was sutured, and the mice were placed on a thermostatic heating pad until they awoke. After 24 h, the mice in the recovery and regeneration process were i.v. injected with 5 μg of WGA-AF488 dye (cat. no. W11261, Thermo Fisher), 2 μg of PE F4/80 antibody (cat. no. 123110, Biolegend, 0.2 mg ml^–1^), 3 μg of Alexa Fluor 647 Ly6G antibody (cat. no. 127610, Biolegend, 0.5 mg ml^–1^) and 70 μl PBS. After 20 min, the mice were deeply anesthetized with tribromoethanol (350 mg kg^–1^, i.p.), and then dissected, exposing the living liver on a home-made holder with a 170-μm-thick glass bottom. To develop AILF, wild-type mice were given i.p. injection of 600 mg kg^–1^ APAP (cat. no. 10024, Cayman) for 16 h, following overnight fasting. Then, the mice with AILF were i.v. injected with 4 μg of CD63 FITC anti-mouse CD63 antibody (cat. no. 143920, Biolegend, 0.5 mg ml^–1^), 10 μg of Alexa Fluor 594 Ly6C antibody (cat. no. NB100-65413AF594, Novus Biologicals, 0.62 mg ml^–1^), 5 μg of APC anti-mouse CD31 antibody (cat. no. 102410, Biolegend, 0.2 mg ml^–1^) and 70 μl PBS. After 20 min, the mice were deeply anesthetized with tribromoethanol (350 mg kg^–1^, i.p.), and then dissected, exposing the living liver on the same home-made holder. During sLFM imaging, the mice were maintained in their normal physiopathogenic states using an instrument to maintain the temperature at 37 °C (ThermoStar Homeothermic Monitoring System, RWD).

### Preparation of BSC-1 cells

The Biologics Standards-Cercopithecus-1 (BSC-1) cell line was purchased from Pricella Life Technology. The cells were cultured under conditions of 5% CO_2_ and 37 °C. Actin-Phalloidin Alexa 555 (Cytoskeleton, phdh1) was used for immunofluorescence staining.

### Preparation of mouse tail slice and rapeseed flower slice

The mouse tail slice and rapeseed flower slice were purchased directly from Sagaoptics to test the performance of SeReNet. The biological sex and age of the samples are unknown.

### Preparation of tumor organoid

Tumor tissues were preserved in Advanced Dulbecco’s Modified Eagle’s Medium with Nutrient Mixture Ham’s F-12 (Ad-DF) (cat. no. 12634, Invitrogen), 1% penicillin–streptomycin, 1% Hepes and 1% GlutaMAX (hereafter referred to as Ad-DF+++). For tissue dissociation, the fresh tissues were minced into 1–3-mm^3^ pieces in a small volume of digestion buffer: Ad-DF+++ supplied with 1 mg ml^–1^ collagenase type I. The tissues were digested on a shaker at 80 rpm at 37 °C for 1 h with occasional pipetting until the visible pieces disappeared. Dissociated cell clusters were washed once with Ad-DF+++ and spined down at 400*g* for 4 min. Dissociated cell clusters were resuspended in 70% cold Matrigel and seeded in a prewarmed 6-well plate at 50-μl drops. During sLFM imaging, a microscope incubator system (Tokai Hit, INUF-IX3D-F1) was used to maintain the environmental conditions of temperature of 37 °C and CO_2_ concentration of 5%.

### Preparation of mouse brain slice

The brain was collected from the Thy1-YFP-H transgenic mouse (Jackson stock no. 003782, male) and placed in 4% PFA overnight at 4 °C. Then the brain was cut into 50-μm-thick slices and sealed in antifade solution (Applygen Technologies, cat. no. C1210) for imaging.

### 2pSAM experiments

For microglia imaging, CX3CR1-GFP transgenic mice (Jax cat. no. 008451, male, 8–12 weeks) were observed 2 weeks after craniotomy. For neutrophil imaging, wild-type C57BL/6J mice (male, 8–12 weeks old) were injected with 3 μg of Alexa-Fluor-647-labeled Ly6G antibody (cat. no. 127610, Biolegend), and cortical layer 2/3 was observed immediately after craniotomy. For neural imaging, Rasgrf2-2A-dCre;Ai148d mice (Jax cat. no. 022864, male, 8–12 weeks) transgenic mice (male, 8–12 weeks) were observed 2 weeks after craniotomy.

### Ethics statement

This work was carried out following all relevant ethical regulations for animal research. All biological experiments were conducted with ethical approval from the Animal Care and Use Committee of Tsinghua University.

### Performance metrics

The r.m.s. error (r.m.s.e.), PSNR and multiscale structural similarity (MS-SSIM) indices were used to evaluate the performance. *X* denotes the reference, and *Y* denotes the output estimation. The r.m.s.e. metric was calculated as:

$$\mathrm{r.m.s.e.}=\sqrt{\mathop{\sum}\limits_{i=1}^{n}\frac{{\Vert{X}_{i}-{Y}_{i}\Vert}_{2}^{2}}{K}},$$

where *K* is the total pixel number in matrix *X* or *Y*. The PSNR metric was calculated as:

$$\mathrm{PSNR}=10{\log}_{10}\frac{\max({\Vert X\Vert}_{2}^{2})}{{\Vert X-Y\Vert }_{2}^{2}}.$$

The MS-SSIM metric was calculated as

$$\mathrm{MS-SSIM}=\mathop{\prod}\limits_{s=1}^{5}\mathrm{SSIM}{({X}_{s},{Y}_{s})}^{{e}^{-\frac{{(s-3)}^{2}}{2}}/{\sum}_{s=1}^{5}{e}^{-\frac{{(s-3)}^{2}}{2}}},$$

where

$$\mathrm{SSIM}=\frac{(2{\mu}_{X}{\mu}_{Y}+{(0.01 \max (X))}^{2})(2{\sigma}_{XY}+{(0.03 \max (X))}^{2})}{({\mu}_{X}^{2}+{\mu}_{Y}^{2}+{(0.01 \max (X))}^{2})({\sigma }_{X}^{2}+{\sigma}_{Y}^{2}+{(0.03 \max (X))}^{2})},$$

*X*_*s*_ and *Y*_*s*_ denote the down-sampling counterparts of *X* and *Y* by a factor of 2^*s*–1^, *µ*_*X*_ and *µ*_*Y*_ denote the average, *σ*_*X*_ and *σ*_*Y*_ denote the s.d. and *σ*_*XY*_ denotes the cross covariance. The local SSIM maps were first calculated using sliding 2D Gaussian windows (11 × 11, s.d. of 1.5) for 2D images and sliding 3D Gaussian windows (11 × 11 × 11, standard deviation 1.5) for 3D stacks. Then the mean value of the local SSIM maps were returned as the SSIM metric.

### Data analysis

The sLFM data acquisition was accomplished using our previously released software GUI (sLFdriver^22^). All data processing and analysis was conducted with Python (3.7 version) scripts and our customized MATLAB (MathWorks, MATLAB 2018b) scripts, including preDAO algorithm (v0.1), TW-Net (v0.1) and SeReNet (v0.1), which can be found at GitHub (https://github.com/kimchange/SeReNet) and Zenodo (10.5281/zenodo.14909862). The 3D rendering in figures and videos was carried out using Amira (Thermo Fisher Scientific, Amira 2019) and Imaris (Imaris 9.0.1 software). To extract the neural activities, the CNMF algorithm^63^ (v1.11.5) was used to derive neuronal segmentations and temporal traces. The temporal traces were calculated by Δ*F*/*F*_0_ = (*F – F*_0_)/*F*_0_, where *F* represents the averaged intensity of the ROI and *F*_0_ denotes the mean value of *F*. The fiber lengths and cell-to-cell distances in Fig. 4 were calculated manually by the biologists. For cell tracking in Figs. 3 and 5, the reconstructed timelapse data were imported into Imaris software, where spots were automatically detected. The quality parameter was set to 120. The 3D tracking was conducted using the built-in autoregressive motion algorithm in Imaris software. Tracked traces shorter than 10 µm were removed. For cell segmentation in Fig. 5e,f, we used Python code for the CellPose algorithm (v1)^64^ with default parameters.

### Statistics and reproducibility

Data shown in Figs. 3–5 and Supplementary Figs. 15, 19–23 and 26 are representative of *n* = 6 experiments. Characterization data shown in Supplementary Figs. 8, 9, 16 and 17 are representative of *n* = 6 experiments. Simulated data shown in Fig. 2 and Supplementary Figs. 1, 3–7, 10–14, 18 and 25 are representative of *n* = 12 experiments.

## Material availability

All other materials that support the findings of this study are available from the corresponding authors upon request.

### Reporting summary

Further information on research design is available in the Nature Portfolio Reporting Summary linked to this article.

## Online content

Any methods, additional references, Nature Portfolio reporting summaries, source data, extended data, supplementary information, acknowledgements, peer review information; details of author contributions and competing interests; and statements of data and code availability are available at 10.1038/s41592-025-02698-z.

## Supplementary information

Supplementary Information Supplementary Figs. 1–26 and Tables 1–3 and titles of Supplementary Videos 1–6.

Reporting Summary

Supplementary Video 1 Comparison of reconstruction methods for visualizing migrasome formation in zebrafish embryos in vivo.

Supplementary Video 2 Comparison of reconstruction methods for visualizing extracellular vesicle dynamics in *Dictyostelium discoideum.*

Supplementary Video 3 Comparison of reconstruction methods for visualizing multi-color identities and calcium dynamics in NeuroPAL transgenic *C. elegans* (strain OH16230).

Supplementary Video 4 SeReNet reveals behaviors and functions of retraction fiber in living mouse livers following LIRI.

Supplementary Video 5 SeReNet reveals the recruitment of monocytes by CD63^+^ endothelial cells in living mouse livers following AILF.

Supplementary Video 6 SeReNet enables rapid 3D reconstruction of two-day-long orchestrated dynamics in zebrafish larvae with and without tailfin injury spanning more than 300,000 time points.

## Source data

Source Data Fig. 2 Statistical source data for Fig. 2.

Source Data Fig. 3 Statistical source data for Fig. 3.

Source Data Fig. 4 Statistical source data for Fig. 4.

Source Data Fig. 5 Statistical source data for Fig. 5.

## Data Availability

The synthetic bubtub dataset and all relevant data for SeReNet have been made publicly available on GitHub (https://github.com/kimchange/SeReNet) and Zenodo (10.5281/zenodo.14909862)^65^. Source data are provided with this paper.

## Code availability

All relevant codes of SeReNet are readily accessible and available on GitHub (https://github.com/kimchange/SeReNet) and Zenodo (10.5281/zenodo.14909862)^65^. Code and example data for drawing comparisons among different networks are also provided.
