# Supplementary material for: Physics-driven self-supervised learning for fast high-resolution robust 3D reconstruction of light-field microscopy
Source: Nat Methods. 2025 May 12;22(7):1545–55. doi: 10.1038/s41592-025-02698-z (PMC12240845; doi:10.1038/s41592-025-02698-z)
Supplement: Supplementary file 1 — Supplementary Figs. 1–26 and Tables 1–3 and titles of Supplementary Videos 1–6. [file 41592_2025_2698_MOESM1_ESM.pdf]

nature methods

Supplementary information

https://doi.org/10.1038/s41592-025-02698-z

Physics-driven self-supervised learning for fast high-resolution robust 3D reconstruction of light-field microscopy

In the format provided by the authors and unedited

Supplementary Information

Physics-driven self-supervised learning for fast high-resolution robust 3D reconstruction of light-field microscopy

Zhi Lu[#], Manchang Jin[#], Shuai Chen, Xiaoge Wang, Feihao Sun, Qi Zhang, Zhifeng Zhao, Jiamin Wu[†], Jingyu Yang[†], Qionghai Dai[†]

I. Supplementary Figures

**Supplementary Figure 1**	Performance comparison of iterative tomography using wave-optics PSFs and geometric-optics PSFs in sLFM reconstruction
**Supplementary Figure 2**	Network architecture of SeReNet
**Supplementary Figure 3**	Ablation study on the three modules of SeReNet with comparisons to other supervised networks
**Supplementary Figure 4**	Convergence process of SeReNet
**Supplementary Figure 5**	Interpretability of SeReNet due to the physics-driven design
**Supplementary Figure 6**	Comparison between RLN and SeReNet on sLFM data
**Supplementary Figure 7**	Comparison between DINER and SeReNet for reconstructing sLFM data
**Supplementary Figure 8**	Resolution characterization of SeReNet in sLFM
**Supplementary Figure 9**	Resolution characterization of SeReNet in LFM without scanning
**Supplementary Figure 10**	Performance of SeReNet on sLFM data with different scanning numbers
**Supplementary Figure 11**	Evaluation of SeReNet using different loss functions under different noise levels

**Supplementary Figure 12**	Comparisons of noise robustness among different methods
**Supplementary Figure 13**	Content-aware motion correction of SeReNet without the need of parameter tuning
**Supplementary Figure 14**	Evaluation of aberration robustness of different methods
**Supplementary Figure 15**	Comparison of SeReNet trained on different datasets
**Supplementary Figure 16**	Pipeline of constructing bubtub simulation dataset and evaluation of generalization
**Supplementary Figure 17**	Finetuning strategy for axially improved SeReNet
**Supplementary Figure 18**	Analysis of the axial finetuning strategy on reconstruction resolution and generalization
**Supplementary Figure 19**	Experimental comparison of SeReNet trained on bubtub and bubtub variant
**Supplementary Figure 20**	Changes of immune microenvironments in mouse livers following LIRI observed by sLFM with SeReNet
**Supplementary Figure 21**	Changes of immune microenvironments in mouse livers following AILF observed by sLFM with SeReNet
**Supplementary Figure 22**	Example slide of CD63+ EC staining using multiplex immunohistochemistry (mIHC)
**Supplementary Figure 23**	Compatibility of SeReNet with various LFM configurations
**Supplementary Figure 24**	Illustration and benchmarking of different reconstruction algorithms for LFM
**Supplementary Figure 25**	Synergizing SeReNet and Vs-Net for rapid high-resolution reconstruction of LFM data
**Supplementary Figure 26**	Applicability of SeReNet for the 3D reconstruction of two-photon synthetic aperture microscopy (2pSAM)

II. Supplementary Tables

**Supplementary Table 1**	Detailed network parameters of SeReNet

**Supplementary Table 2**	Comparison of algorithm performance and efficiency between four light-field reconstruction strategies
**Supplementary Table 3**	Parameters for fluorescence experiments

III. Supplementary Videos

**Supplementary Video 1**	Comparison of reconstruction methods for visualizing migrasome formation in zebrafish embryos *in vivo*
**Supplementary Video 2**	Comparison of reconstruction methods for visualizing extracellular vesicle dynamics in *Dictyostelium discoideum*
**Supplementary Video 3**	Comparison of reconstruction methods for visualizing multi-color identities and calcium dynamics in NeuroPAL transgenic *C. elegans* (strain OH16230)
**Supplementary Video 4**	SeReNet reveals behaviors and functions of retraction fiber in living mouse livers following LIRI
**Supplementary Video 5**	SeReNet reveals the recruitment of monocytes by CD63+ endothelial cells in living mouse livers following AILF
**Supplementary Video 6**	SeReNet enables rapid 3D reconstruction of two-day-long orchestrated dynamics in zebrafish larvae with and without tailfin injury spanning over 300,000 time points

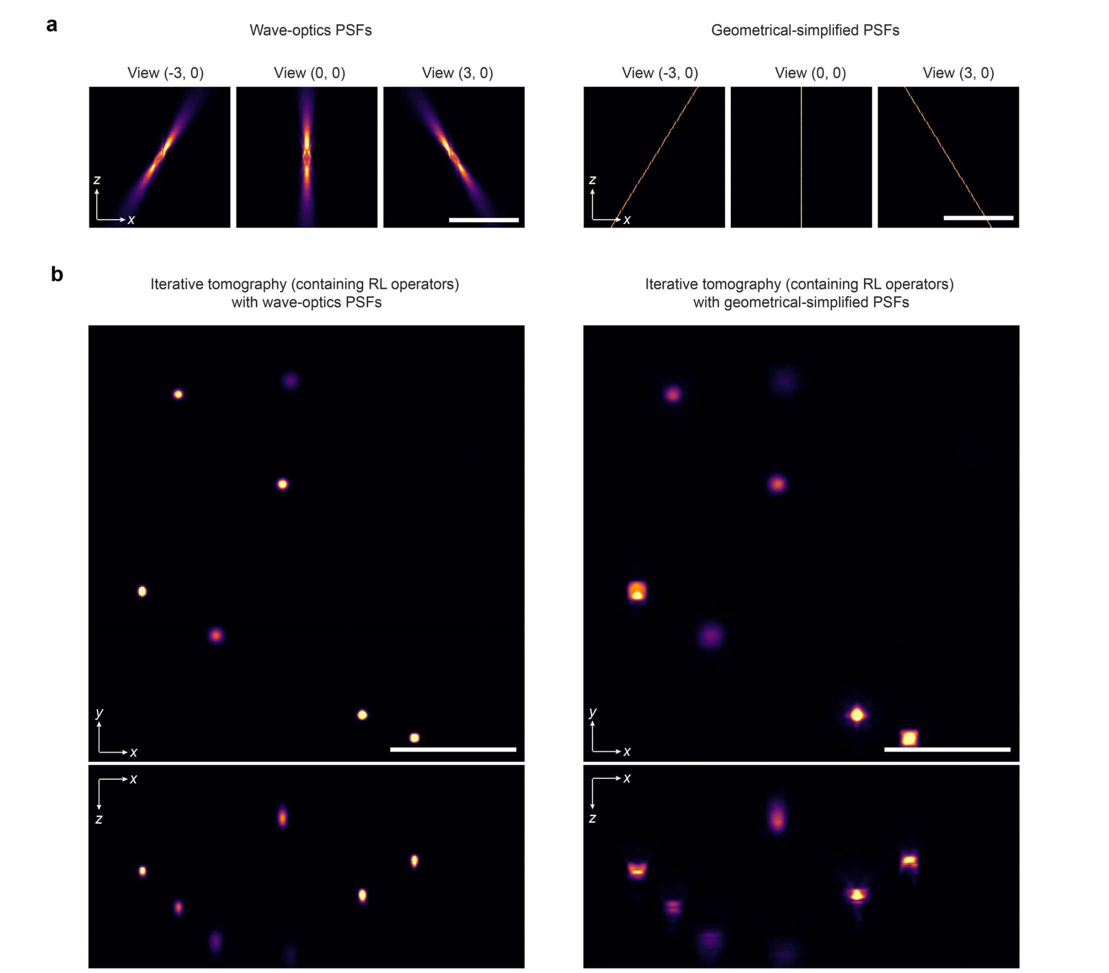

Supplementary Figure 1 | Performance comparison of iterative tomography using wave-optics PSFs and geometric-optics PSFs in sLFM reconstruction. a, The *xz* maximum intensity projections (MIPs) of wave-optics PSFs (left) and geometrical-simplified PSFs (right) in sLFM system configurated with a 63×/1.4 NA oil-immersion objective. Example angular components are shown. **b**, Orthogonal MIPs of 100-nm diameter fluorescence beads, reconstructed by iterative tomography using wave-optics PSFs (left) and geometrically simplified PSFs (right). During iterative tomography, RL operators serve as mathematical constraints for algorithm convergence. However, relying solely on RL operators is insufficient, resulting in poor performance when using geometrically simplified PSFs. Conversely, wave-optics PSFs, which accurately account for the physical model, enable the recovery of high resolution. Scale bars, 10 μm (**a-b**).

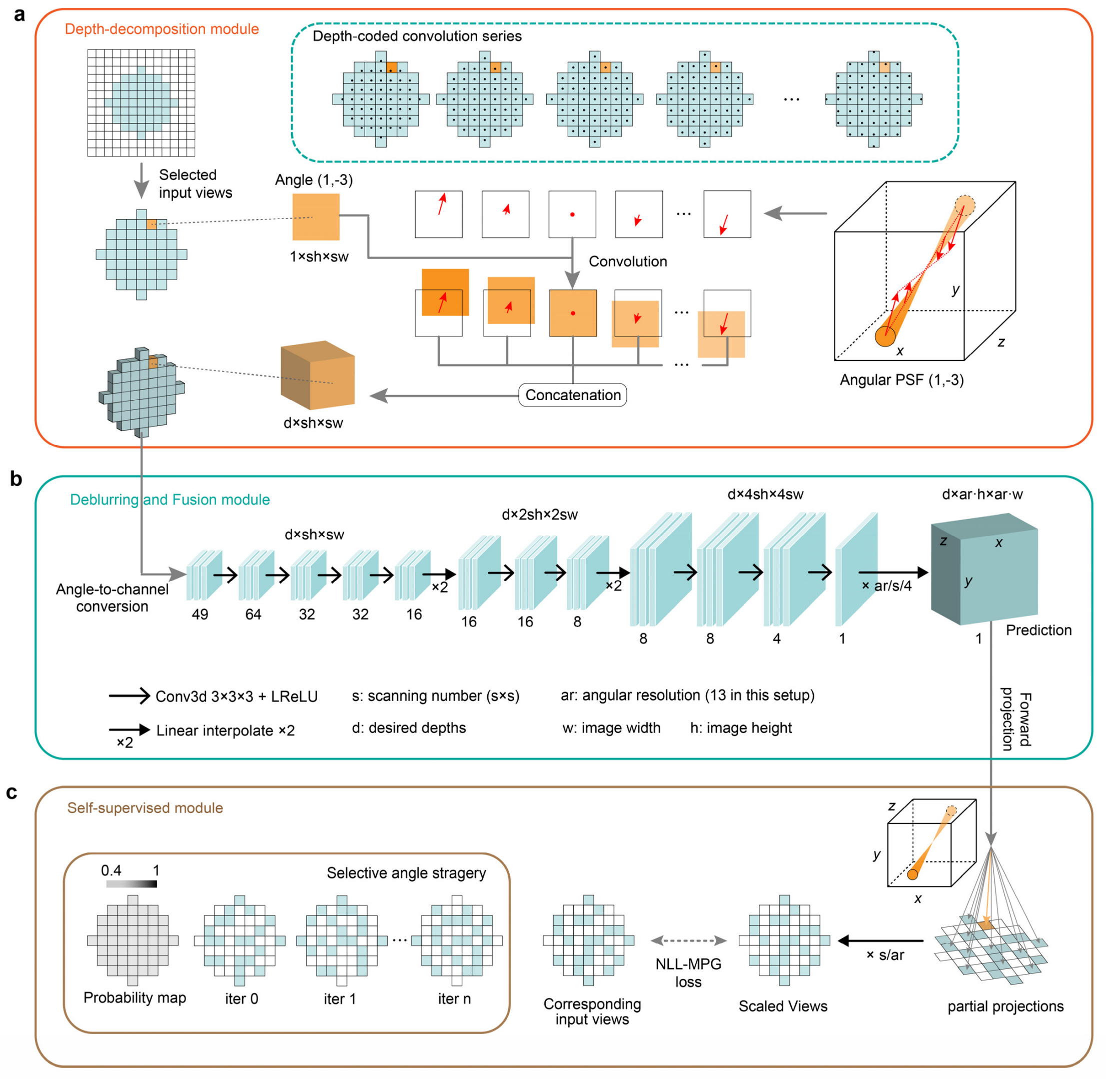

Supplementary Figure 2 | Network architecture of SeReNet. a, Depth-decomposition module. As indicated by PSFs of sLFM, sample signals at different depths cause image shifts at varying distances in different spatial-angular measurements, where the ratio of distance to depth is related to the angle between angular PSF and the optical axis. A depth-coded convolution series is applied to transform the angular measurements into refocused volumes along sub-aperture beam propagation directions. Each block in depth-coded convolution series represents a single view of spatial-angular components, with black points indicating convolution centers. Traditional convolution is a specific case of depth-coded convolution series. For samples not confined to the focal plane, depth-coded convolution is applied at different spatial positions, determined by angular PSFs. The lower row shows convolution operators at different depths along the angle of (1, -3), with red arrows indicating convolution centers. Convolution results at various depths are concatenated to obtain refocused 3D features along different angles. 49 views near the microlens center are used, consistent with iterative tomography. **b**, Deblurring and fusion module. A convolutional network with nine 3D convolutional layers and three linear interpolation layers enhances the 3D features and transforms them into a 3D volume. The number of channels for

intermediate features is listed below the network. **c**, Self-supervised module. The output volume from the deblurring and fusion module is projected to spatial-angular images using different angular PSFs. These spatial-angular projections are scaled to match the pixel size of measurements. The loss between projections and corresponding measurements is calculated and minimized to optimize network parameters via backpropagation. Due to limited GPU memory, 21 randomly selected views (indicated by blue patches) are used for loss calculation in each iteration.

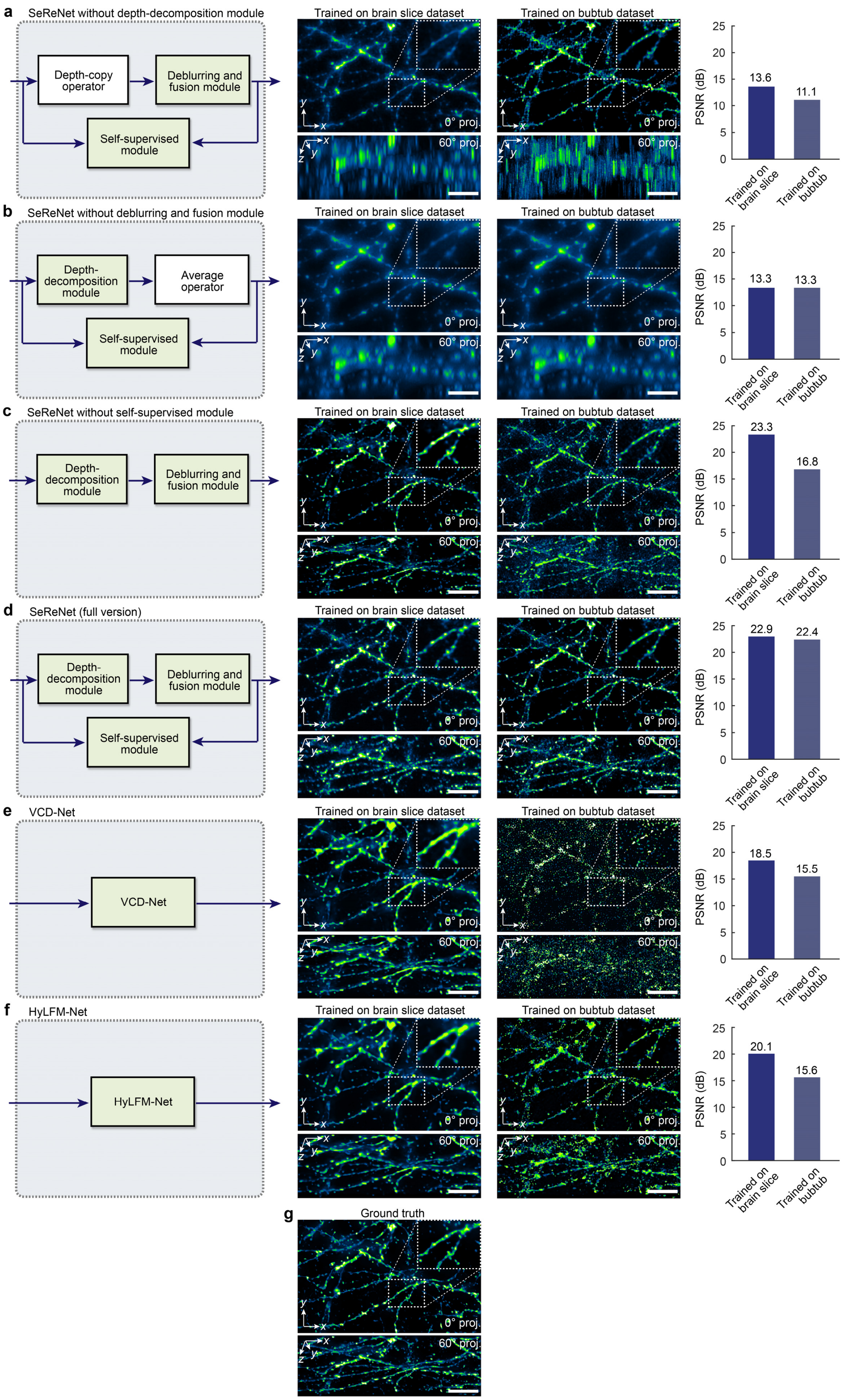
a
SeReNet without depth-decomposition module
Depth-copy operator
Deblurring and fusion module
Self-supervised module
Trained on brain slice dataset
Trained on bubtub dataset
0° proj.
60° proj.
PSNR (dB)
13.6
11.1
Trained on brain slice
Trained on bubtub
b
SeReNet without deblurring and fusion module
Depth-decomposition module
Average operator
Self-supervised module
13.3
13.3
c
SeReNet without self-supervised module
Depth-decomposition module
Deblurring and fusion module
23.3
16.8
d
SeReNet (full version)
Depth-decomposition module
Deblurring and fusion module
Self-supervised module
22.9
22.4
e
VCD-Net
18.5
15.5
f
HyLFM-Net
20.1
15.6
g
Ground truth

Supplementary Figure 3 | Ablation study on the three modules of SeReNet with comparisons to other supervised networks. **a**, Left, simplified schematic of SeReNet without depth-decomposition module. Right, orthogonal MIPs of a mouse brain slice captured by sLFM, and then reconstructed by the corresponding method trained on brain slice dataset and synthetic bubtub dataset respectively, with bar charts to show comparison of PSNR indices. **b**-**f**, Corresponding results using different networks, including SeReNet without deblurring and fusion module (**b**), SeReNet without self-supervised module (**c**), SeReNet (**d**), VCD-Net (**e**) and HyLFM-Net (**f**). The depth-decomposition module was replaced by a depth-copy operator in (**a**), and the deblurring and fusion module was replaced by an average operator in (**b**), to meet the requirements of the subsequent architectures. **g**, Ground-truth counterpart, which was captured by confocal microscopy with 60×/1.42NA objective lens. The input data for networks is the spatial-angular images of the ground truth through forward projection with PSFs of sLFM. The brain slice dataset consists of 107 volumes captured from confocal microscopy, and uses the PSFs of sLFM for forward projection to obtain spatial-angular images to create training pairs, which were not involved in network predictions. The synthetic bubtub dataset can refer to Supplementary Fig. 16a. SeReNet without depth-decomposition module, SeReNet without deblurring and fusion module, SeReNet were only trained on spatial-angular images without supervision, while SeReNet without self-supervised module, VCD-Net and HyLFM-Net were trained in a supervised way using spatial-angular images as inputs and high-resolution 3D volumes as targets. Scale bar, 20 μm (**a**-**g**).

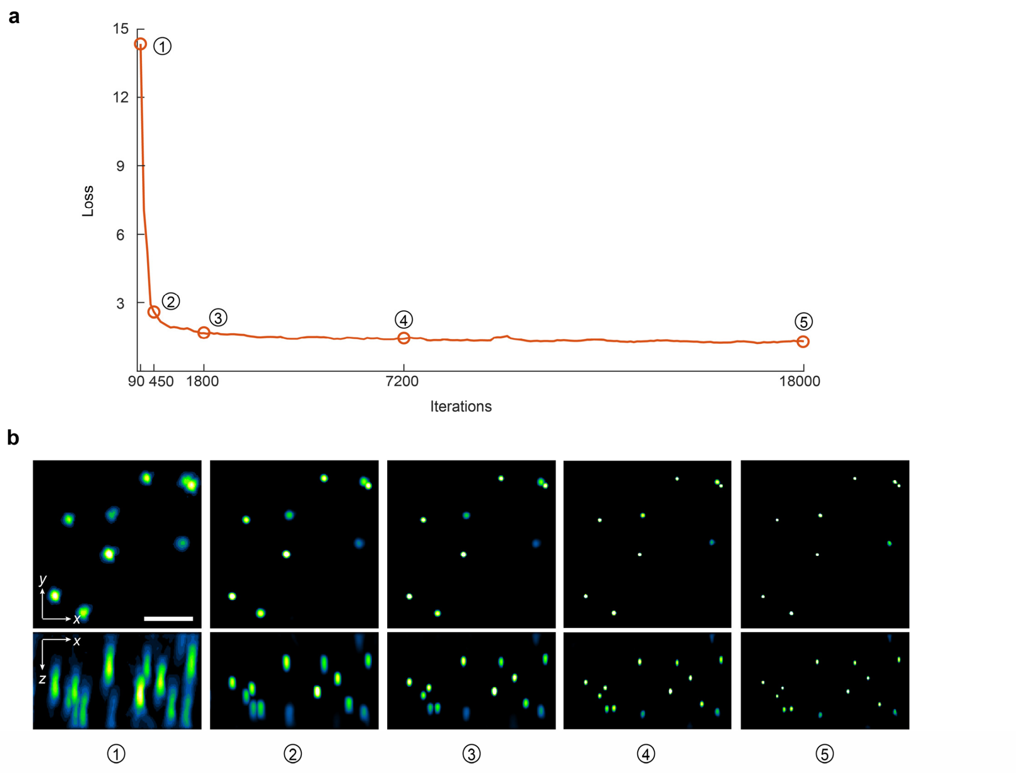

Supplementary Figure 4 | Convergence process of SeReNet. a, Convergence curves of SeReNet. All parameters were randomly initialized before network training. As the number of iterations increases, the loss between spatial-angular views estimated by SeReNet and actual measurements decreases iteratively, demonstrating that SeReNet converges to a valid solution. **b**, Representative intermediate results on 100-nm-diameter beads. The images show progressively improved performance at different iterations during the training phase. Scale bar, 10 µm (**b**).

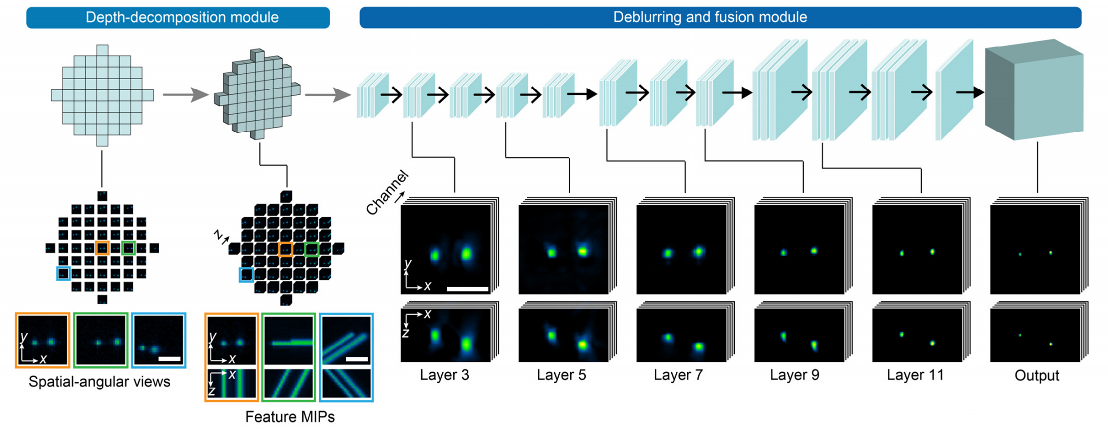

Supplementary Figure 5 | Interpretability of SeReNet due to the physics-driven design. The inference process of SeReNet comprises the depth-decomposition module and the deblurring and fusion module, enabling high-resolution 3D reconstruction. We visualize the intermediate 3D features of different layers using 100-nm-diameter beads as input data. As data flows through the network, the bead centers become highlighted while the blurry edges are suppressed. Scale bars, 5 μm.

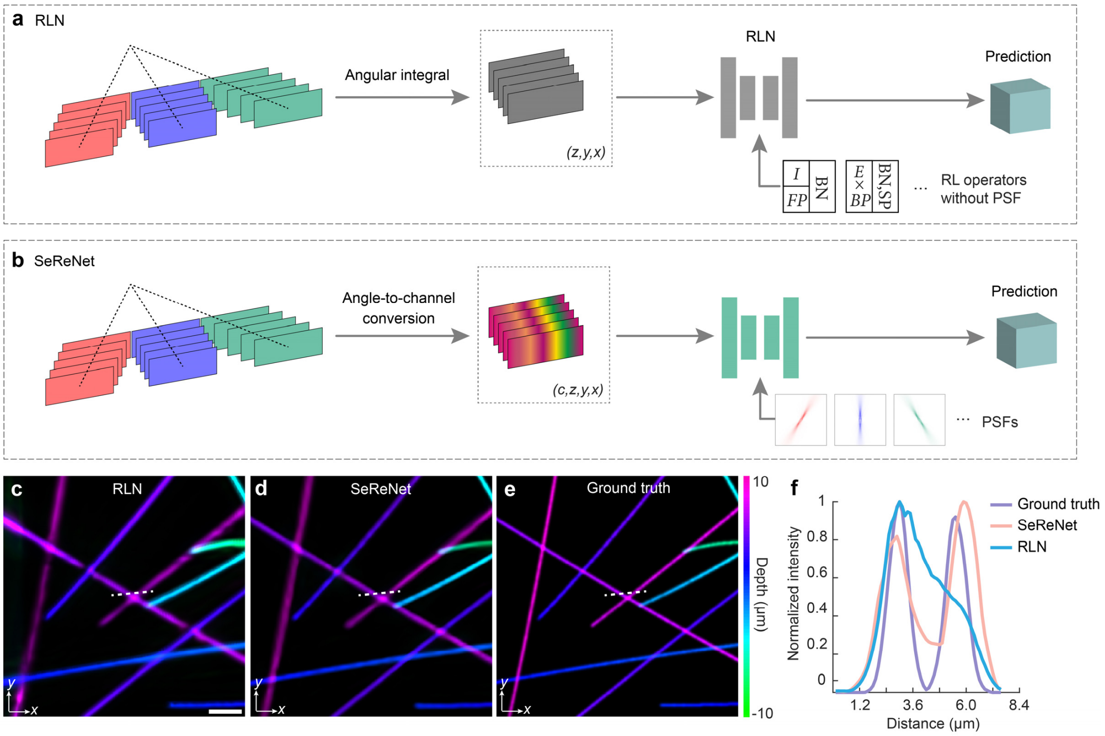

Supplementary Figure 6 | Comparison between RLN and SeReNet on sLFM data. a-b, Simplified schematics of Richardson-Lucy Network (RLN, **a**) and SeReNet (**b**). SeReNet leverages the PSF priors as the physical constraints to prevent overestimating information intrinsically not captured in the imaging system, while RLN applies operators to provide mathematical constraints without PSF introduced. To adapt RLN to sLFM data, we first digitally refocused the spatial-angular measurements into a volume, using these as input and the corresponding iterative tomography reconstructions as targets for RLN training (Methods section). Both networks were trained on synthetic tubulin dataset not involved in network predictions. **c-e**, MIPs and enlarged regions of 0.3-μm-diameter tubulins, reconstructed by RLN (**c**) and SeReNet (**d**). Ground truth is shown in **e**. **f**, Normalized intensity profiles along the dashed white lines in **c-e**, indicating that physics-driven SeReNet outperforms RLN with mathematical priors. Scale bars, 5 μm (**c-e**).

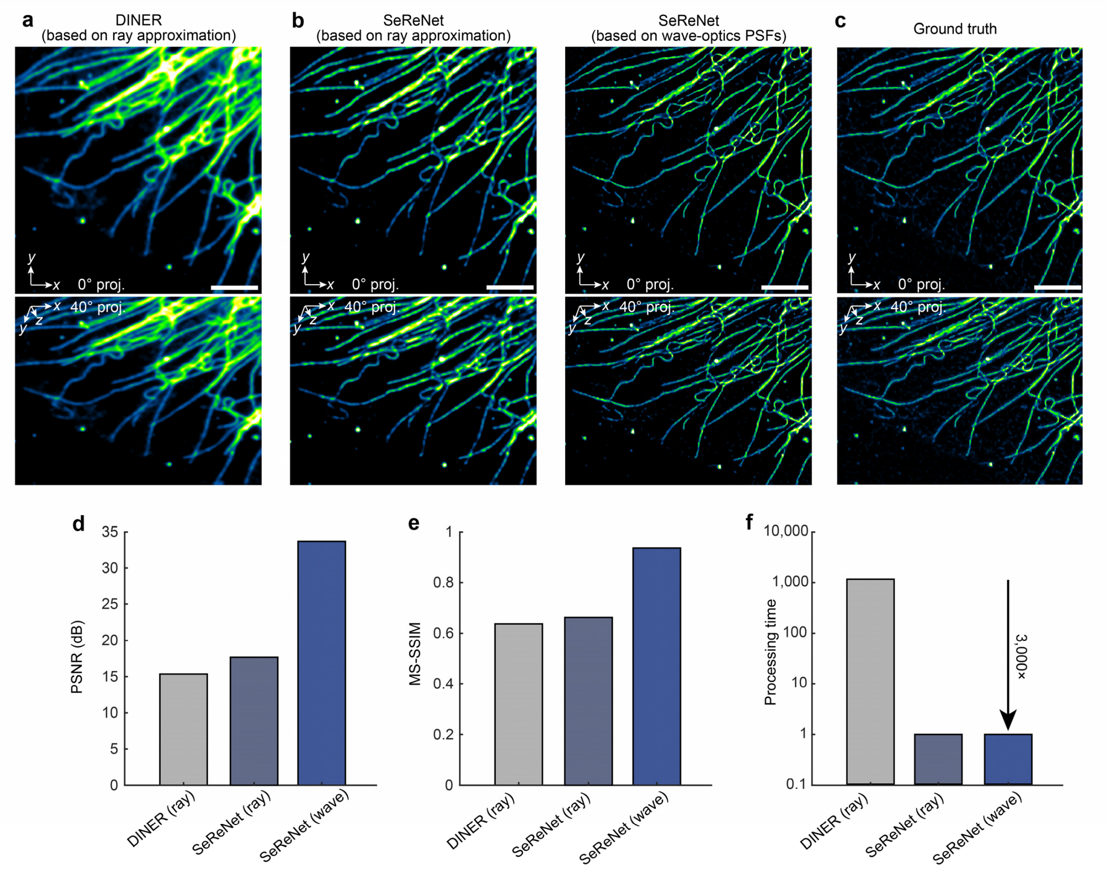

Supplementary Figure 7 | Comparison between DINER and SeReNet for reconstructing sLFM data. a, Orthogonal MIPs and enlarged regions of an actin-labelled BSC-1 cell captured by sLFM in simulation, and then reconstructed by DINER, which is based on ray approximation. **b**, Corresponding results using two SeReNet models, which are based on ray approximation and wave-optics PSFs, respectively. The former SeReNet model was trained by geometrically simplifying the wave-optics PSFs, as shown in Supplementary Fig. 1a. **c**, Ground-truth counterpart, which was captured by confocal microscopy with 60×/1.42NA objective lens. The input data for DINER and SeReNet is the spatial-angular images of the ground truth through forward projection with PSFs of sLFM. **d-e**, Bar chart showing PSNR (**d**) and MS-SSIM (**e**) for comparing DINER (based on ray approximation), SeReNet (based on ray approximation) and SeReNet (based on wave-optics PSFs). **f**, Bar chart showing the comparison of processing time. In this experiment, DINER model was trained on the actin data, and SeReNet models were trained on the synthetic bubtub dataset (Supplementary Fig. 16a). Scale bars, 20 μm (**a-c**).

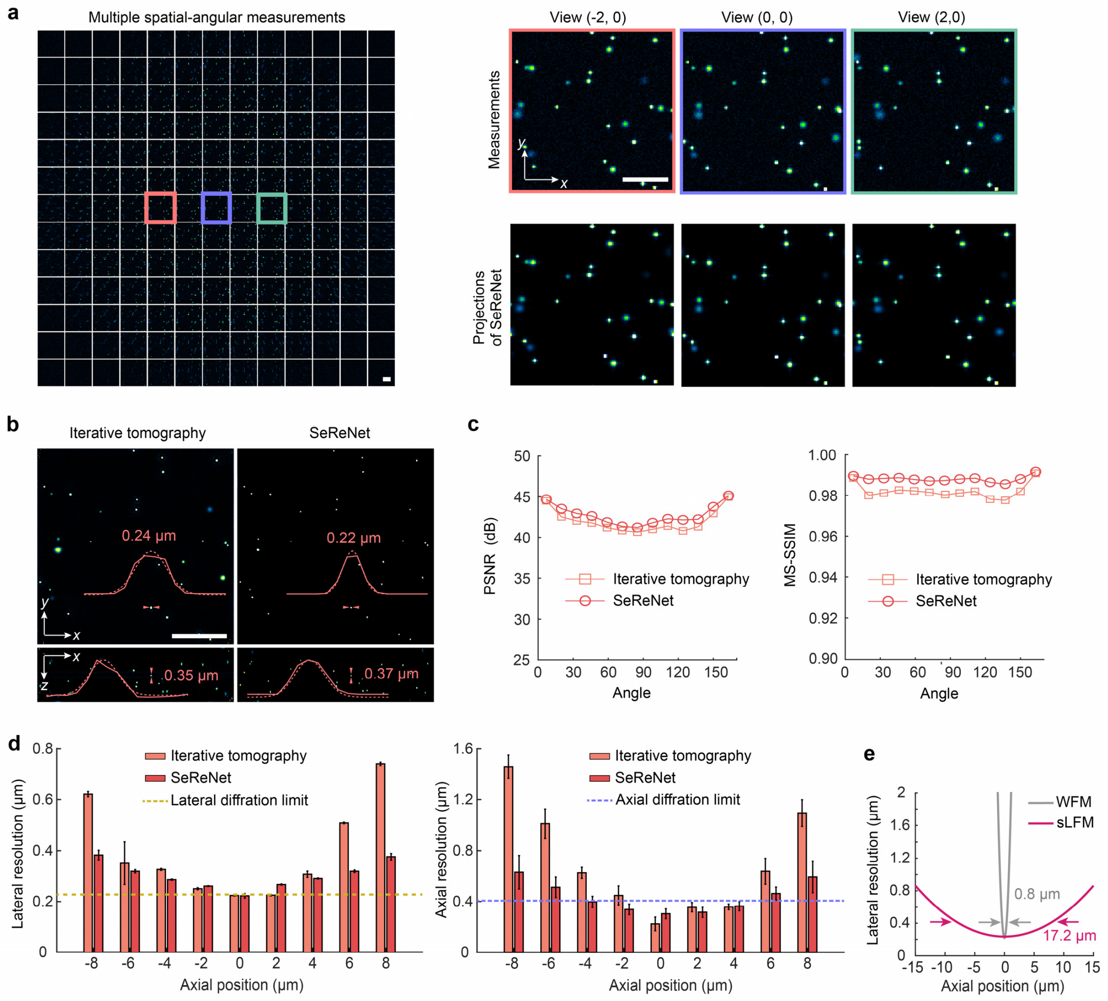

Supplementary Figure 8 | Resolution characterization of SeReNet in sLFM. a, The left side displays multiple spatial-angular measurements of 100-nm fluorescence beads randomly distributed in low-melt agarose, captured by sLFM with a 63×/1.4 NA oil-immersion objective. Enlarged example angular views are shown in the upper-right corner. The lower-right corner presents projections along corresponding angles from SeReNet results, demonstrating the consistency. **b**, Orthogonal MIPs and achieved FWHMs by iterative tomography and SeReNet. **c**, The PSNR and MS-SSIM curves versus different angles applied for distinct methods. Indices were calculated on projections along corresponding angles of reconstruction results, with the spatial-angular measurements of sLFM as ground truth. **d**, Boxplots of averaged lateral resolution (left) and axial resolution (right) between iterative tomography (n = 7,559 beads) and SeReNet (n = 9,798 beads) at different axial positions. Resolutions were estimated by measuring the FWHMs with a Gaussian fit. Dashed lines mark diffraction-limit resolutions at a center wavelength of 525 nm, using the equations described in Methods section. Error bars represent standard deviations. Although the resolution slightly degrades as the axial defocus distance increases, SeReNet shows more uniform resolution performance across axial coverage compared to iterative tomography. **e**, Comparison on depth of field of sLFM and WFM through simulation, showing sLFM has an extended depth of field for volumetric imaging. The depth of field is defined as the axial range

where the resolution remains at half of its optimal value. In this experiment, SeReNet was trained on the fluorescence bead dataset captured by sLFM not involved in resolution characterization, and the SeReNet is the self-supervised version without axial finetuning. Scale bars, 20 μm.

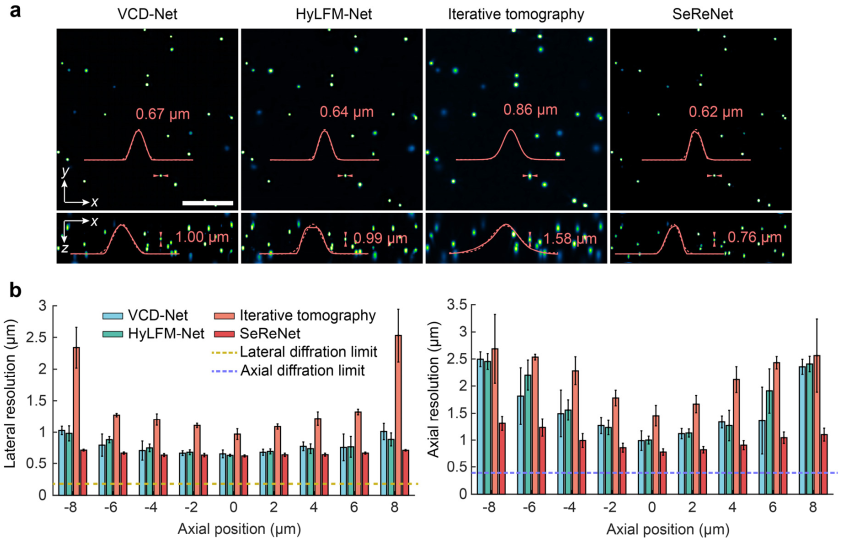

Supplementary Figure 9 | Resolution characterization of SeReNet in LFM without scanning. **a**, Orthogonal MIPs and corresponding FWHMs reconstructed by VCD-Net, HyLFM-Net, iterative tomography and SeReNet with the same input data captured by LFM without scanning. **b**, Boxplots of averaged lateral resolution (left) and axial resolution (right) among VCD-Net, HyLFM-Net, iterative tomography and SeReNet at different axial positions. Resolutions were estimated by measuring the FWHMs of 100-nm fluorescence beads with a Gaussian fit. Dashed lines mark diffraction-limit resolutions at a center wavelength of 525 nm, using the equations in Methods section. Error bars represent standard deviations. In this experiment, SeReNet was trained on the fluorescence bead dataset captured by LFM not involved in resolution characterization, and the SeReNet is the self-supervised version without axial finetuning. VCD-Net and HyLFM-Net were trained using the fluorescence bead dataset captured by LFM as inputs and corresponding sLFM reconstructions by iterative tomography as targets. Scale bars, 20 μm.

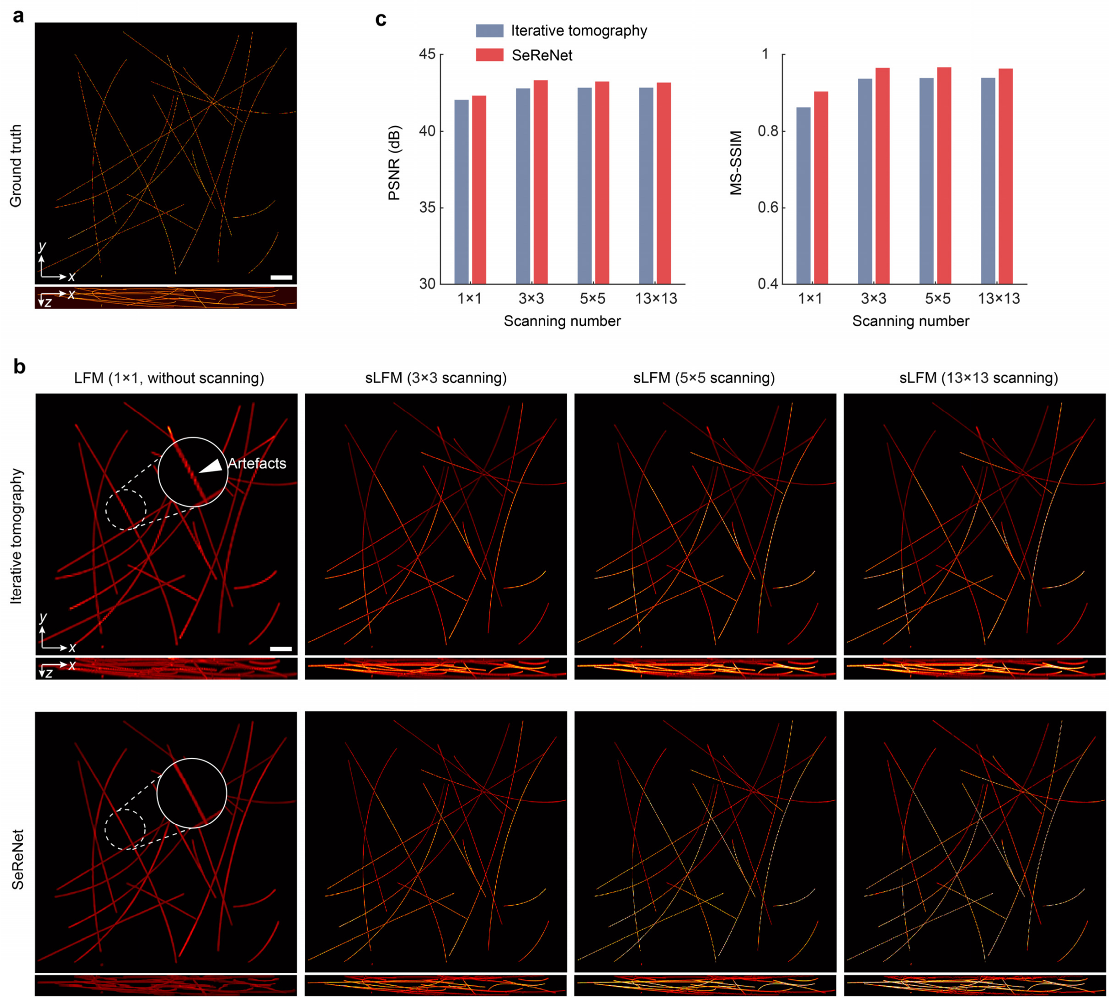

Supplementary Figure 10 | Performance of SeReNet on sLFM data with different scanning numbers. **a**, Synthetic 0.3-μm diameter tubulins, used as ground truth. **b**, Orthogonal MIPs of the reconstruction results obtained by iterative tomography and SeReNet with different scanning numbers. **c**, Bar chart showing PSNR (left) and MS-SSIM (right) for iterative tomography and SeReNet across different scanning numbers. SeReNet demonstrates stable performance in all conditions, indicating its applicability to LFM or sLFM with varying scanning numbers. The networks were trained on synthetic tubulin dataset captured by sLFM with different scanning numbers that were not involved in network predictions. Scale bars, 20 μm (**a**-**b**).

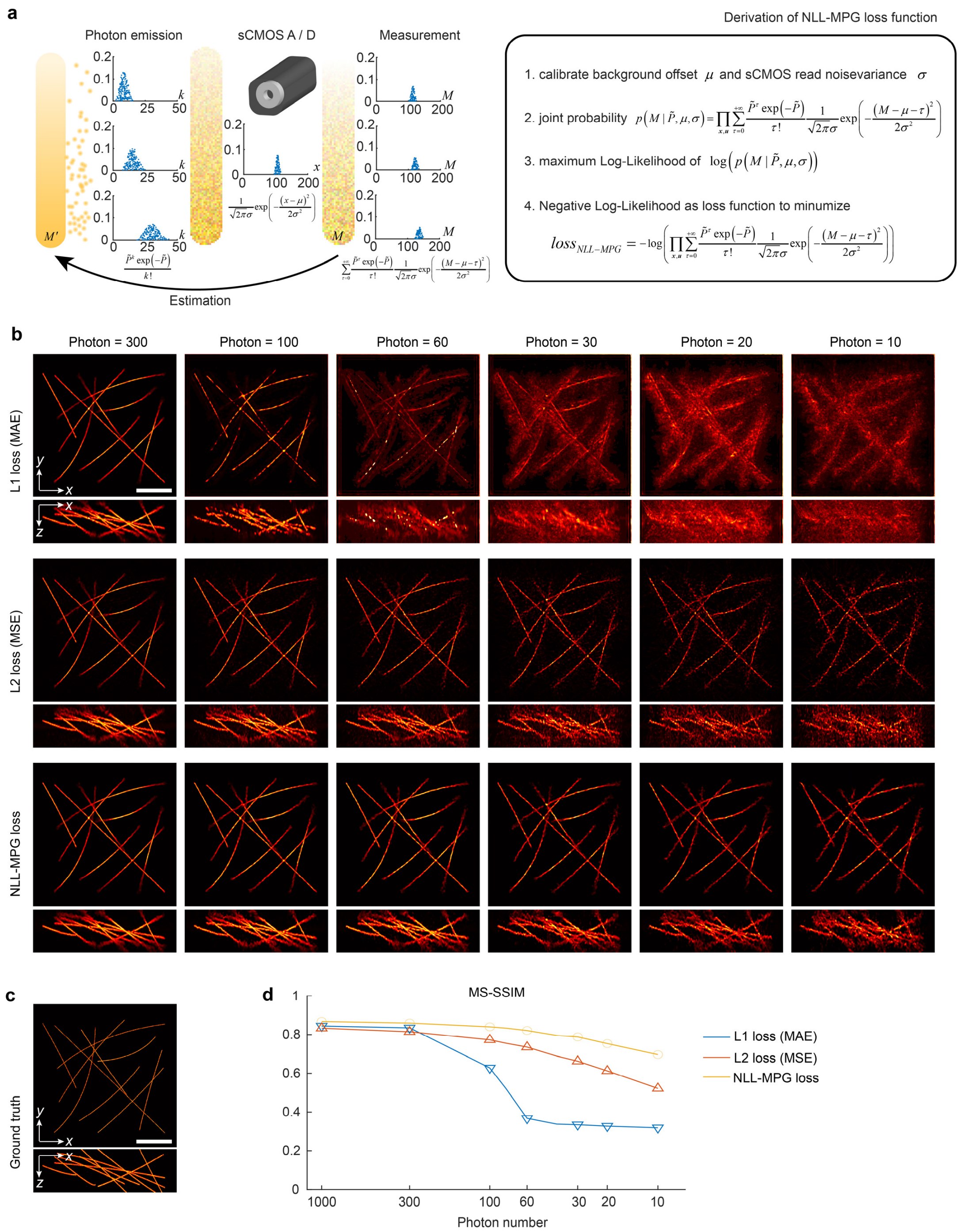

Supplementary Figure 11 | Evaluation of SeReNet using different loss functions under different noise levels. a, Explanation of the NLL-MPG loss derivation. **b**, Orthogonal MIPs of SeReNet with L1 loss (upper), L2 loss (middle) and NLL-MPG loss (lower) at increasing noise levels indicated by the reduction of maximum photon number. Simulation parameters include a 16-bit image depth, Gaussian noise variance of 9, and maximum intensity photon numbers of 100, 30, 10, and 3. **c**, Synthetic 1-μm diameter tubulins, used as ground truth. **d**, Curves of MS-SSIM versus photon numbers applied for SeReNet with different loss functions. Indices were calculation on *xz* MIPs. SeReNet was trained on synthetic tubulin dataset that were not involved in network inference. Scale bars, 20 μm (**b-c**).

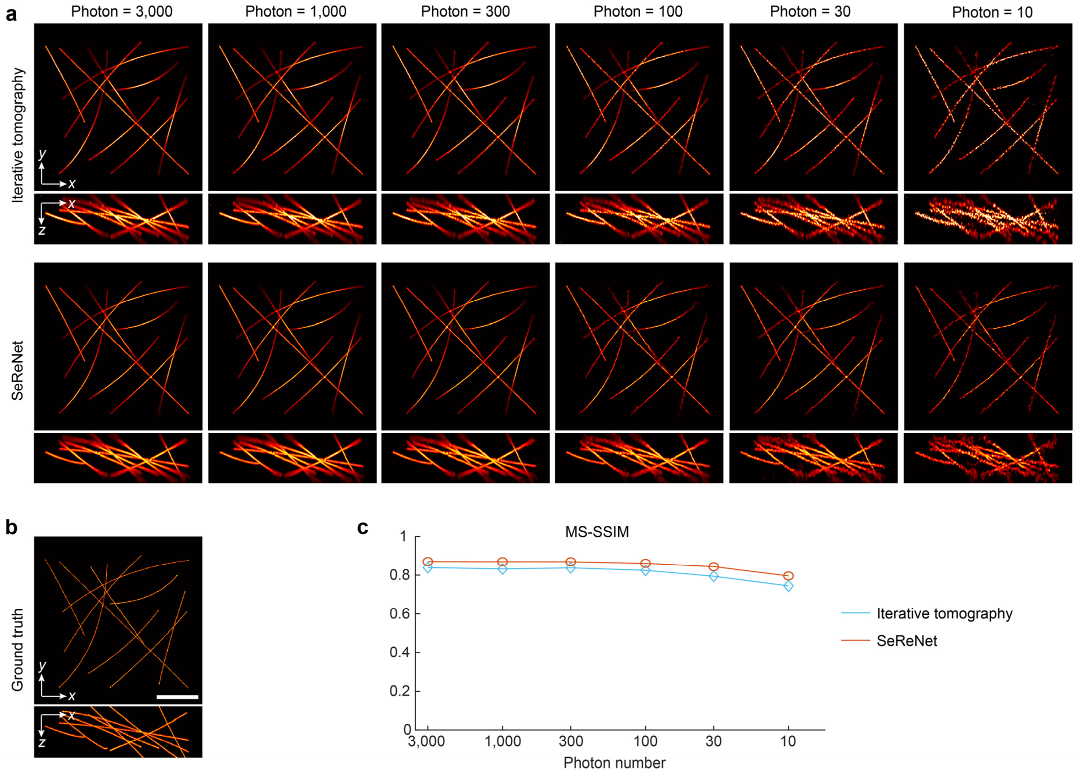

Supplementary Figure 12 | Comparisons of noise robustness among different methods. a, Orthogonal MIPs of iterative tomography and SeReNet at increasing noise levels, indicated by the reduction of maximum photon number. Simulation parameters include a 16-bit image depth, Gaussian noise variance of 5, and maximum intensity photon numbers of 3000, 1000, 300, 100, 30, and 10. **b**, Ground truth. **c**, Curves of MS-SSIM versus varying noise levels applied for different methods. Indices were calculated on *xz* MIPs. SeReNet was trained on synthetic tubulin dataset that were not involved in network inference. Scale bars, 20 μm (**a-b**).

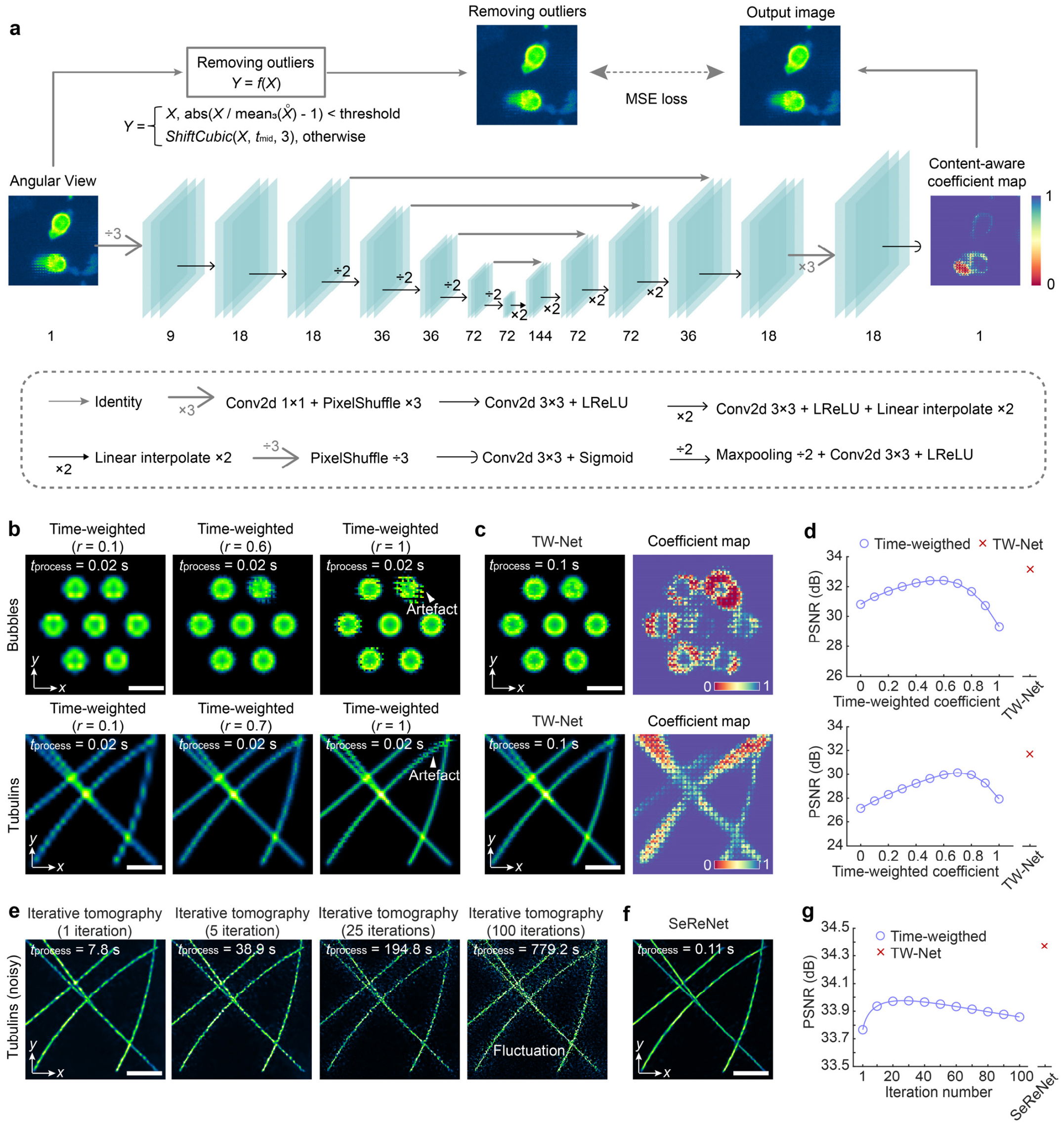

Supplementary Figure 13 | Content-aware motion correction of SeReNet without the need of parameter tuning. a, Structure of TW-Net. A lightweight self-supervised network with 13 layers is developed to correct the nonrigid motion artefacts in 9-frame acquisitions of sLFM before reconstruction. The input sLFM data is transformed into 9-frame LFM data. A U-Net with skip connection extracts motion information features, followed by a sigmoid function to generate a coefficient map within [0, 1]. This map is then applied to a time-weighted algorithm to produce a corrected image as output. In the meantime, an outlier removal algorithm processes the input data to create a reference with minimal motion artifacts. The loss function is defined as the MSE between image after removing outliers and network output. **b**, Center views of synthetic beads (upper row) and tubulins (bottom row) with artificially induced motions, which were processed by different time-weighted coefficients to remove artefacts, albeit with degrade resolution. **c**, TW-Net counterpart removed the artefacts without parameter tunning. Estimated coefficient maps are shown on the right. **d**, PSNR curves for different methods and coefficients, showing TW-Net achieves higher fidelity compared to the time-weighted algorithm. **e**, MIPs of synthetic tubulins

captured by sLFM in low-SNR conditions and reconstructed by iterative tomography with varying iteration numbers. Increased iteration numbers lead to fluctuation-like artefacts that degrade reconstruction quality. **f**, SeReNet counterpart showing robustness against noise without parameter tunning. **g**, PSNR curves for different methods and coefficients, highlighting SeReNet's capability to maintain high resolution while reducing artefacts. Processing times for each subfigure are labelled in the insets. SeReNet was trained on synthetic tubulin dataset, implemented after TW-Net corrected sample motions. Scale bars, 10 μm (**b-c**, **e-f**).

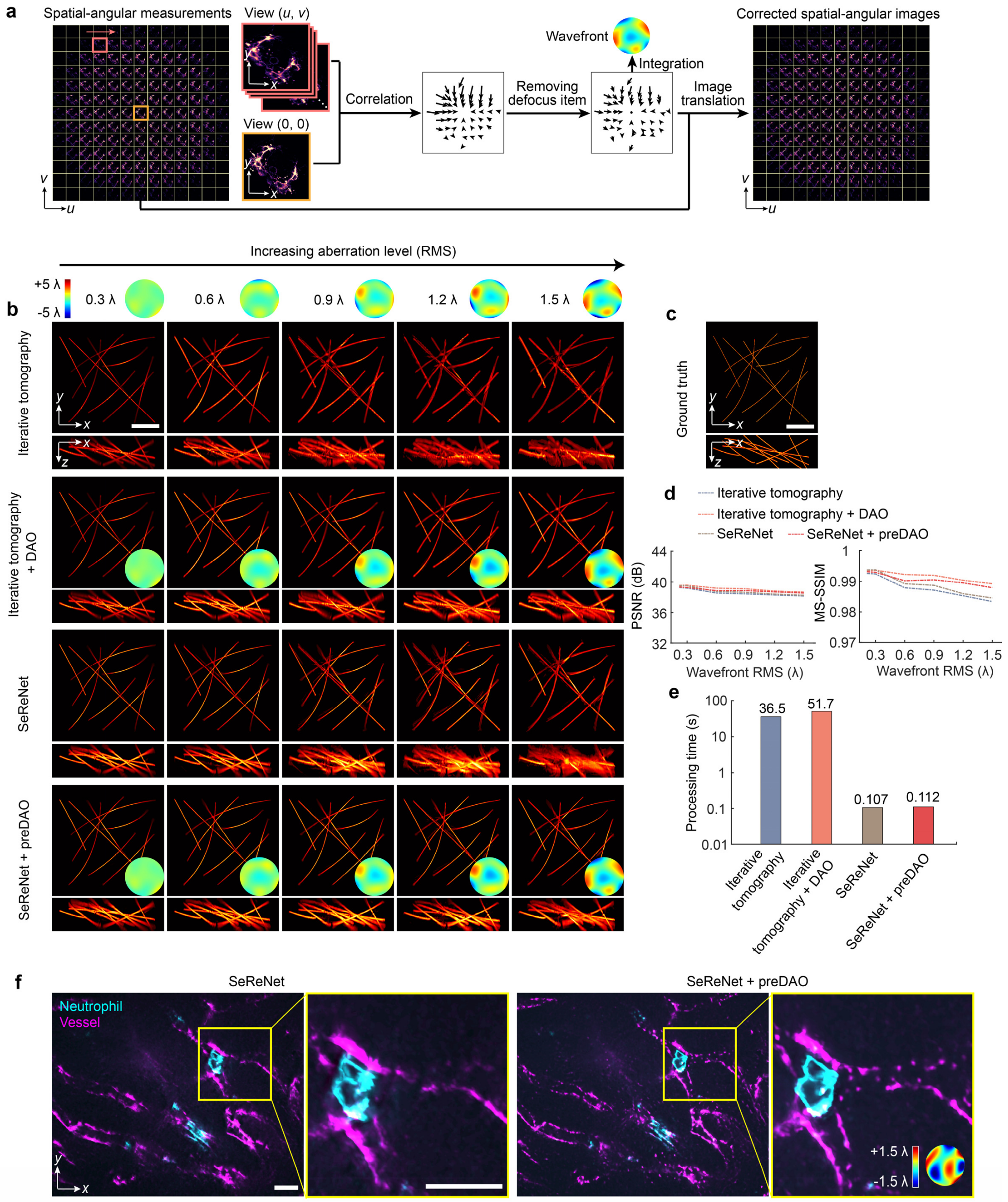

Supplementary Figure 14 | Evaluation of aberration robustness of different methods. a, Illustration of preDAO algorithm. Aberration-induced disparities between center view and other angular views are computed as a 2D shift map using correlation operators. After removing the defocus component, this shift map is utilized to estimate the wavefront and correct spatial-angular images. **b**, First row, randomly generated wavefronts with increasing root mean square (RMS) values, as artificially introduced aberrations. Second row to last row, corresponding results reconstructed by iterative tomography, iterative tomography with DAO, SeReNet and SeReNet with preDAO, respectively. Insets depict the wavefronts estimated by DAO in iterative tomography and preDAO used for SeReNet. Networks were trained on synthetic tubulin dataset

without aberrations that were not involved in network predictions. **c**, Ground truth. **d**, Curves of PSNR (upper row) and MS-SSIM (lower row) versus different levels of induced aberrations for various methods. **e**, Time required for processing a volume covering 737 × 737 × 121 voxels for each method. **f**, The reconstructed MIPs and enlarged regions of neutrophils (cyan) in vessels (magenta) of a living mouse liver, obtained by SeReNet without and with preDAO. SeReNet was trained on synthetic bubtub dataset. Scale bars, 20 μm (**b-c**, **f**).

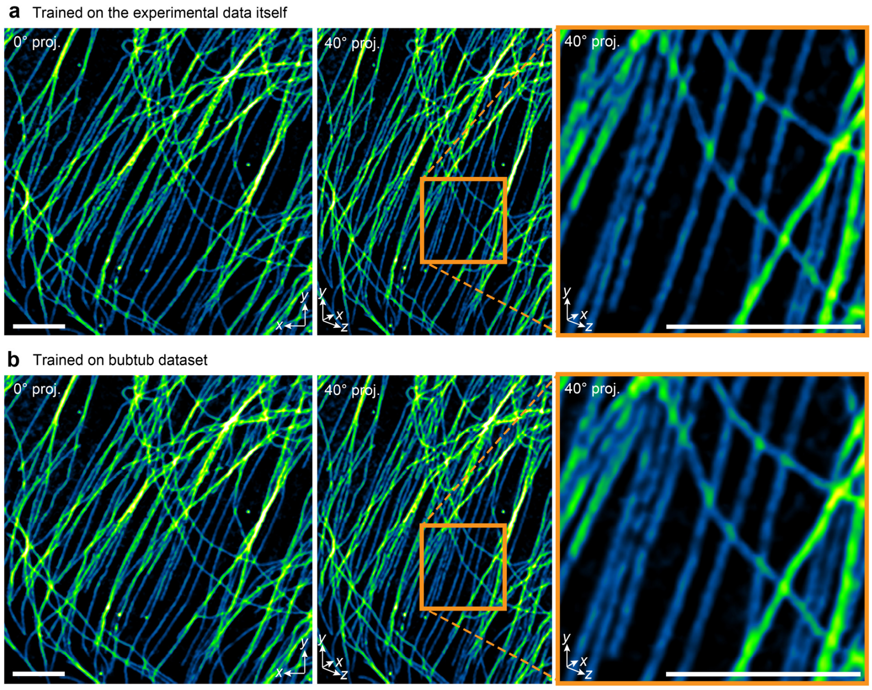

Supplementary Figure 15 | Comparison of SeReNet trained on different datasets. a, Two reconstructed MIPs and enlarged region of an actin-labelled BSC-1 cell captured by sLFM, and then obtained by SeReNet directly trained on the experimental data itself. **b**, Corresponding results obtained by SeReNet trained on the synthetic bubtub dataset. Scale bars, 10 μm.

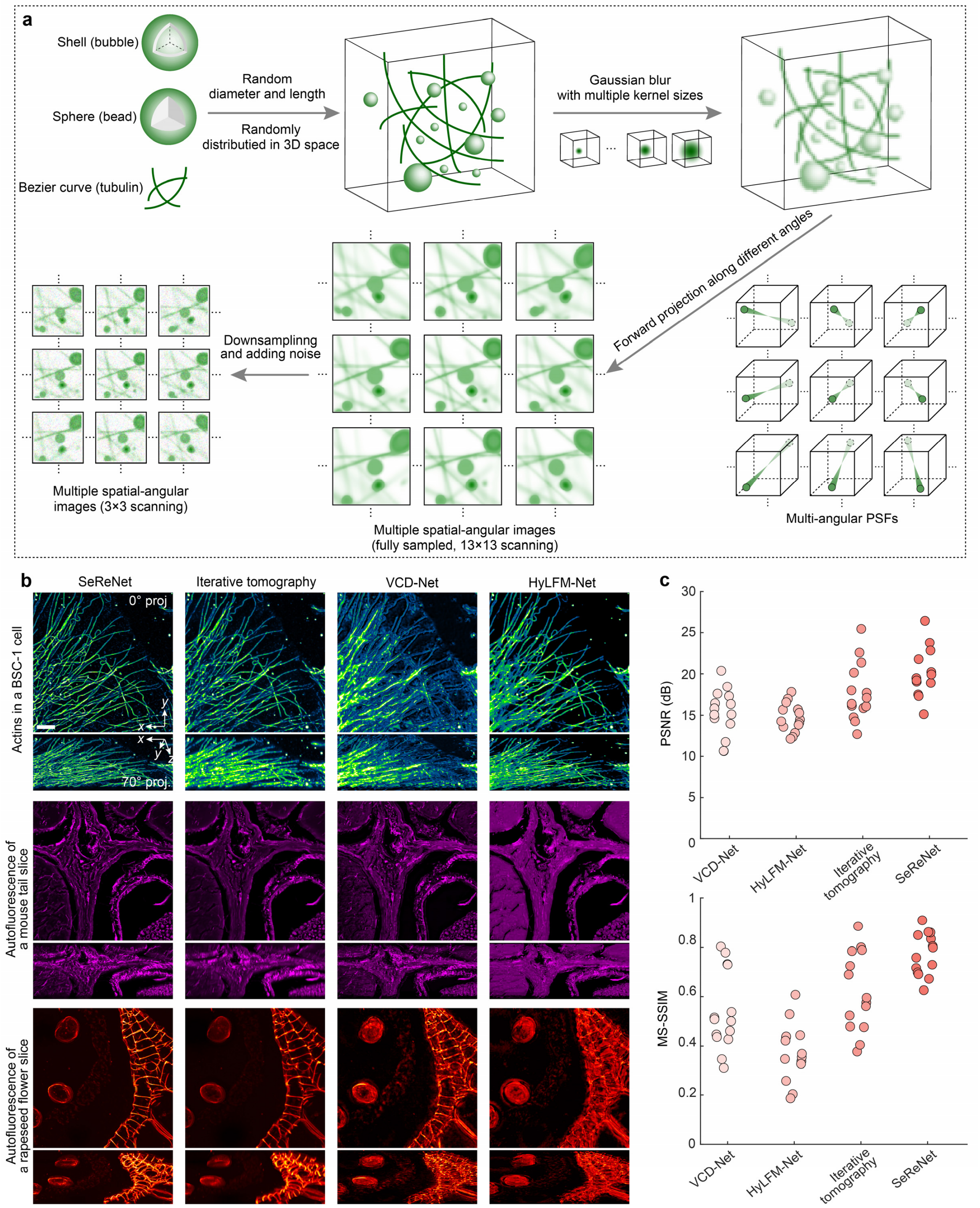

Supplementary Figure 16 | Pipeline of constructing bubtub simulation dataset and evaluation of generalization. a, Three structures including shells, spheres and Bezier curves with random diameters and lengths, are randomly distributed in 3D space, to create a groundtruth volume. The volume is then blurred using Gaussian kernels of varying sizes to simulate real-world conditions. Next, the blurry volume is projected into the spatial-angular domain along the multi-angular PSFs. To simulate real imaging conditions similar to sLFM, the dataset undergoes down-sampling and the addition of MPG noise, resulting in multiple spatial-angular images with a 3×3 scanning configuration, named the bubtub dataset. Ground truth volumes are also included when training axially improved SeReNet. **b**, Generalization results from the bubtub dataset to sLFM images of actins in a BSC-1 cell (upper row), autofluorescence of a mouse tail slice (middle row),

autofluorescence of a rapeseed flower slice (bottom row), reconstructed by SeReNet, iterative tomography, VCD-Net and HyLFM-Net. **c**, Comparison of PSNR and MS-SSIM indices among different reconstruction methods. This figure expands upon Figs. 2j and 2k for further comparisons. Scale bar, 20 μm (**b**).

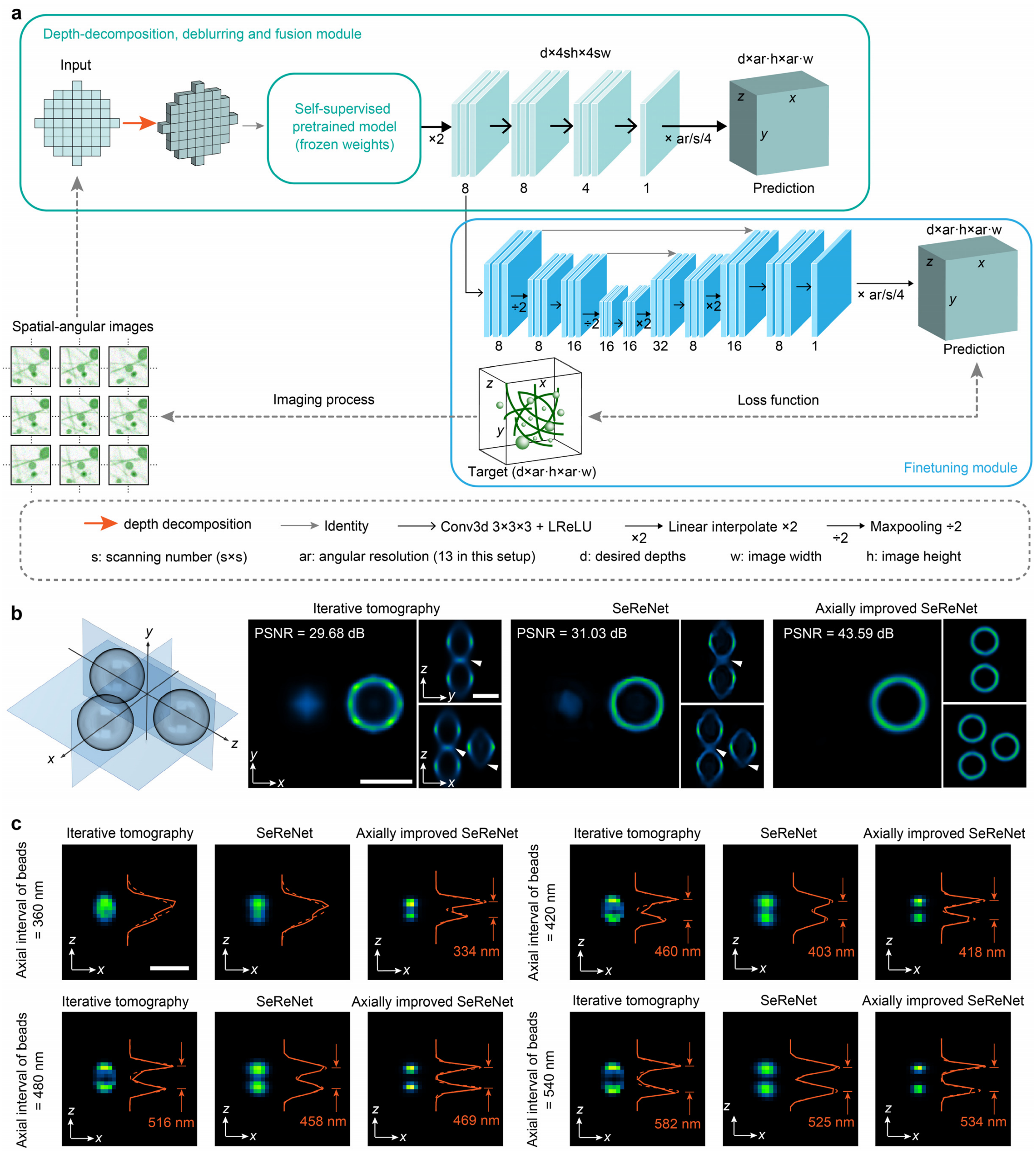

Supplementary Figure 17 | Finetuning strategy for axially improved SeReNet. a, A finetuning network module is introduced to enhance the axial performance of SeReNet. It consists of four 3D convolutional layers, two pooling layers, and three linear interpolation layers, replacing the final 3 layers of SeReNet. The module refines intermediate features into the final 3D output volume. Training follows a two-step strategy: first, fully pretraining the self-supervised SeReNet (Supplementary Fig. 2). Then, using a limited set of synthetic bubtub pairs to optimize the finetuning module while keeping the self-supervised weights frozen. The loss function compares the volume predicted by the finetuning module to the target. **b**, Left, simulation geometry comprising three 5-μm-diameter spherical shells close together. Right, cross-sectional slices along *xy*, *xz* and *yz* directions reconstructed by iterative tomography, SeReNet and axially improved SeReNet. **c**, MIPs of two virtually-separated beads with cross-section profiles along the dashed lines, acquired by sLFM and reconstructed by iterative tomography, SeReNet and axially

improved SeReNet, respectively. We imaged the same 100-nm bead at two positions with specific axial interval (360 nm, 420 nm, 480 nm and 540 nm, respectively) shifted by a piezo translation stage and added the images together to create the two virtually-separated beads. The intensity profiles after reconstruction were subsequently fitted with Gaussian functions, and the distance between the two peaks was measured. Due to the proximity to the diffraction limit, this measured distance slightly deviates from the actual displacement of the translation stage. All networks were trained on the synthetic bubtub dataset. Scale bars, 5 μm (**b**) and 1 μm (**c**).

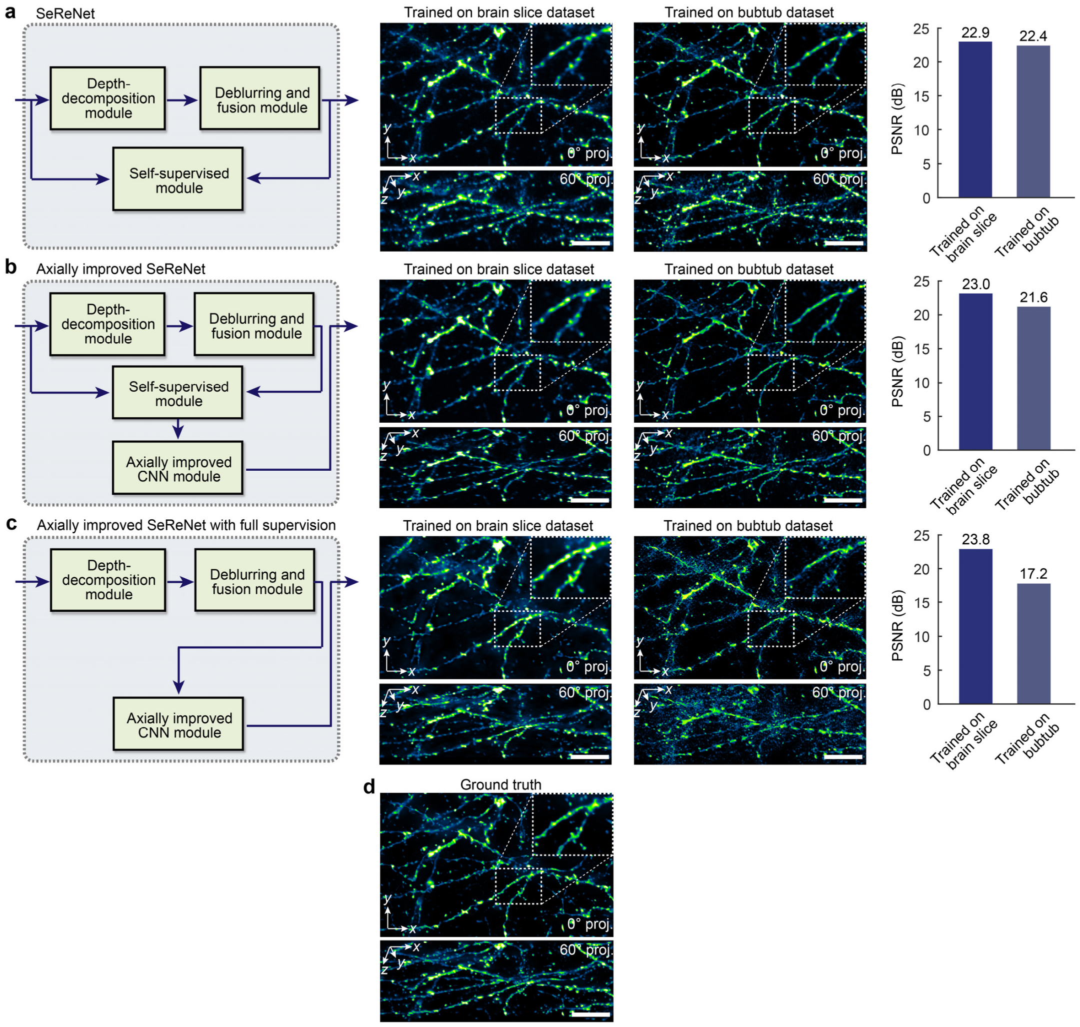

Supplementary Figure 18 | Analysis of the axial finetuning strategy on reconstruction resolution and generalization. a, Left, simplified schematic of SeReNet. Right, orthogonal MIPs of a mouse brain slice captured by sLFM, and then reconstructed by the corresponding method trained on brain slice dataset and synthetic bubtub dataset, with bar charts to show comparison of PSNR indices. **b-c**, Corresponding results using different networks, including axially improved SeReNet (**b**) and fully-supervised (**c**). **d**, Ground-truth counterpart, which was captured by confocal microscopy with 60×/1.42NA objective lens. The input data for networks is the spatial-angular images of the ground truth through forward projection with PSFs of sLFM. The information of brain slice dataset and bubtub dataset can refer to Supplementary Fig. 3. SeReNet was only trained on spatial-angular images without supervision, while fully-supervised SeReNet, It is noted that axially improved SeReNet was pretrained in a self-supervised way, and then finetuned using supervised data pairs. Scale bar, 20 μm (**a-d**).

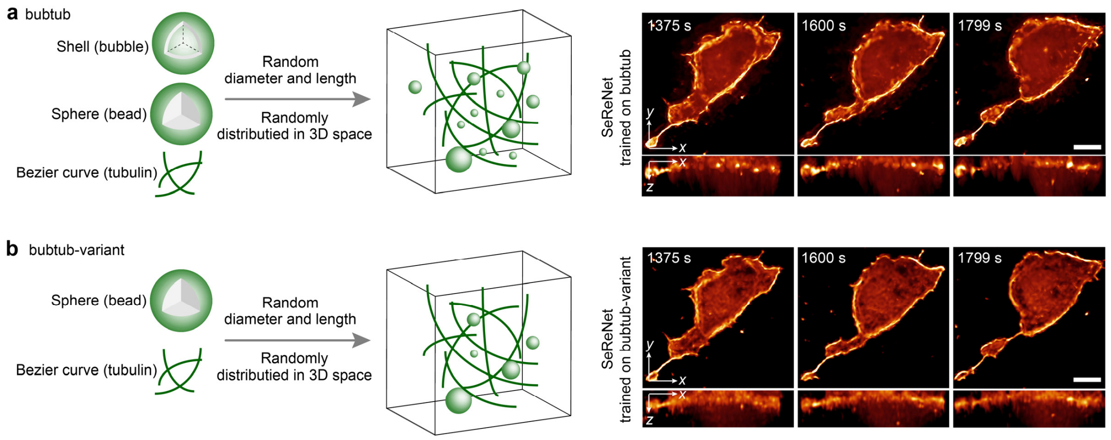

Supplementary Figure 19 | Experimental comparison of SeReNet trained on bubtub and bubtub variant. a, Orthogonal MIPs showing the process of migrasome formation in a zebrafish embryo, obtained by SeReNet trained on bubtub dataset (**a**) and bubtub-variant dataset without shells (**b**). This figure expands upon Fig. 3a to provide additional comparisons. Scale bars, 10 μm (**a-b**).

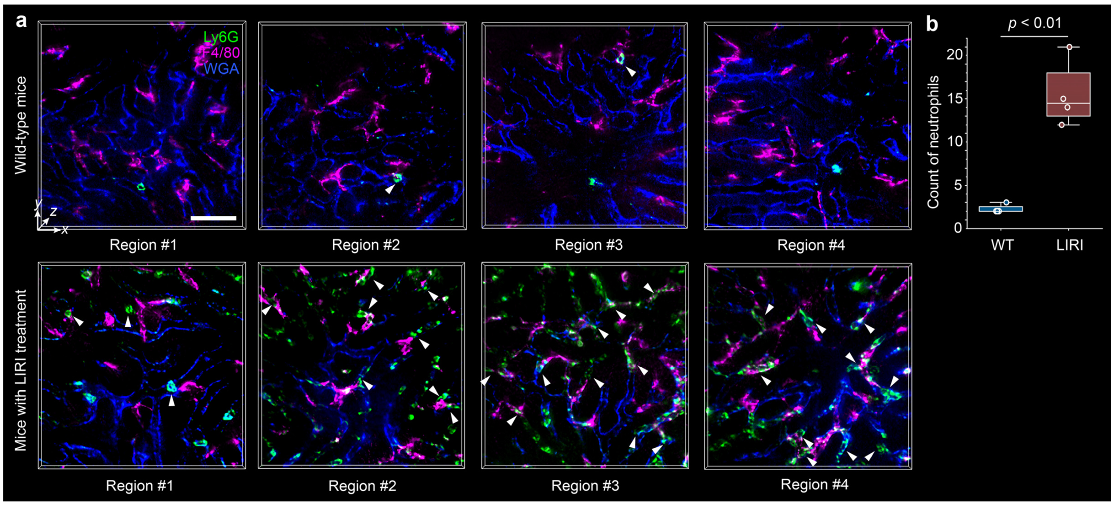

Supplementary Figure 20 | Changes of immune microenvironments in mouse livers following LIRI observed by sLFM with SeReNet. a, Four representative regions of KCs (F4/80, magenta) and neutrophils (Ly6G, green) within liver vessels (WGA, blue) of mice without (upper row) and with LIRI surgery (lower row). Volumes were obtained by SeReNet and rendered using Imaris software. SeReNet was trained on the synthetic bubtub dataset. LIRI surgery was performed 24 hours prior to sLFM imaging, during the mice's recovery and regeneration process. Increased neutrophil numbers are observed after LIRI, with white arrows indicating interactions between neutrophils and KCs. **b**, Boxplot showing the counts of neutrophils in mouse livers without and with LIRI surgery. The boxplot format: center line, median; box limits, lower and upper quartiles; whiskers, 0th–100th percentiles. $n = 4$ represents the number of regions. P values were calculated by the two-sided paired t-test. $P = 5.89\times10^{-3}$. Scale bars, 50 µm (**a**).

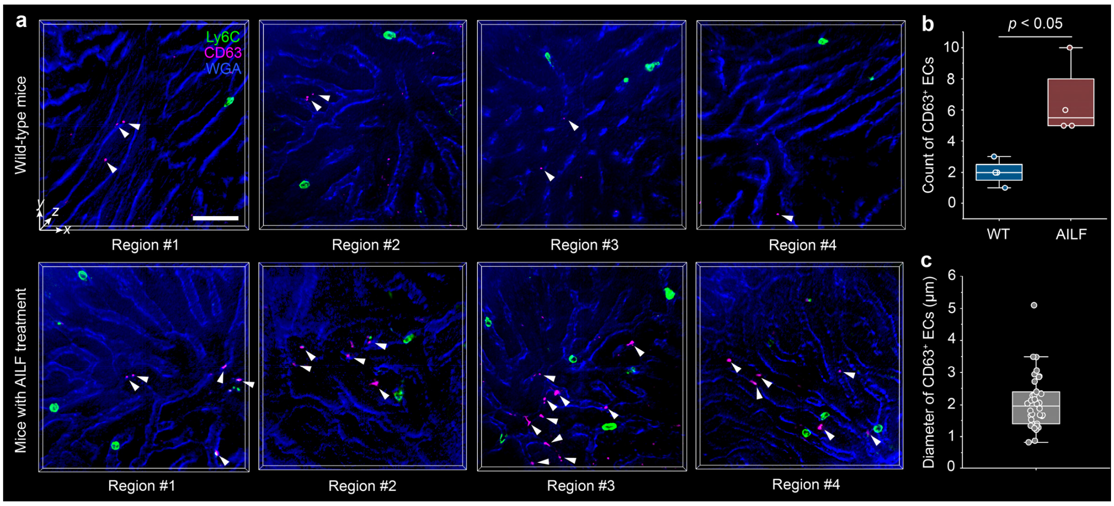

Supplementary Figure 21 | Changes of immune microenvironments in mouse livers following AILF observed by sLFM with SeReNet. a, Four representative regions depicting EC subtype ($CD63^+$, magenta) and monocytes (Ly6C, green) in liver vessels (WGA, blue) of mice without (upper row) and with AILF (lower row). Volumes were obtained by SeReNet and rendered using Imaris software. The network was trained on the synthetic bubtub dataset. Mice were treated with 600 mg/kg of APAP for 16 h before sLFM imaging, to establish AILF models. $CD63^+$ ECs showed an increased presence during AILF, indicated by white arrows. **b**, Boxplot showing the count of $CD63^+$ ECs in mouse livers without and with AILF. The boxplot format: center line, median; box limits, lower and upper quartiles; whiskers, 0th–100th percentiles. n = 4 represents the number of regions. P values were calculated by the two-sided paired t-test. $P = 1.17\times10^{-2}$. **c**, Boxplot showing the quantified diameters of $CD63^+$ EC markers, indicating sizes ranging from 1 to 3 μm. Diameter measurements were based on FWHM calculations of intensity profiles across $CD63^+$ EC markers using Gaussian fitting. Statistical analysis was conducted with n = 32 manually counted cells. The boxplot format: center line, median; box limits, lower and upper quartiles; whiskers, 0th–100th percentiles. Scale bars, 50 μm (**a**).

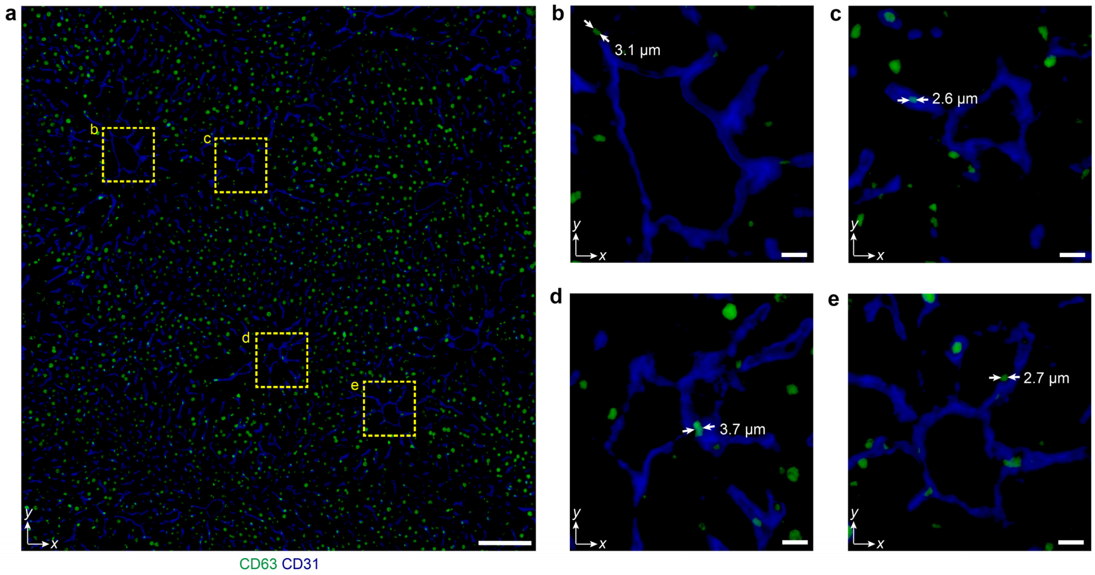

Supplementary Figure 22 | Example slide of CD63$^+$ EC staining using multiplex immunohistochemistry (mIHC). a, Whole slide of a mIHC in an APAP-challenged mouse liver section stained for ECs (CD31$^+$, blue) and a specific EC subtype (CD31$^+$CD63$^+$, green). Mice were exposed to APAP for 16 hours prior to tissue collection. A 4-µm-thick liver section was stained and imaged using the Aperio Versa 8 tissue imaging system (Leica). **b-e**, Enlarged regions from panel **a**, showcasing CD63$^+$ ECs, which are identified by their dual staining with fluorescent proteins and are approximately 3 µm in size. These structures are smaller than cell nuclei, emphasizing the need for subcellular resolution to accurately detect them. Scale bars, 100 µm (**a**) and 10 µm (**b-e**).

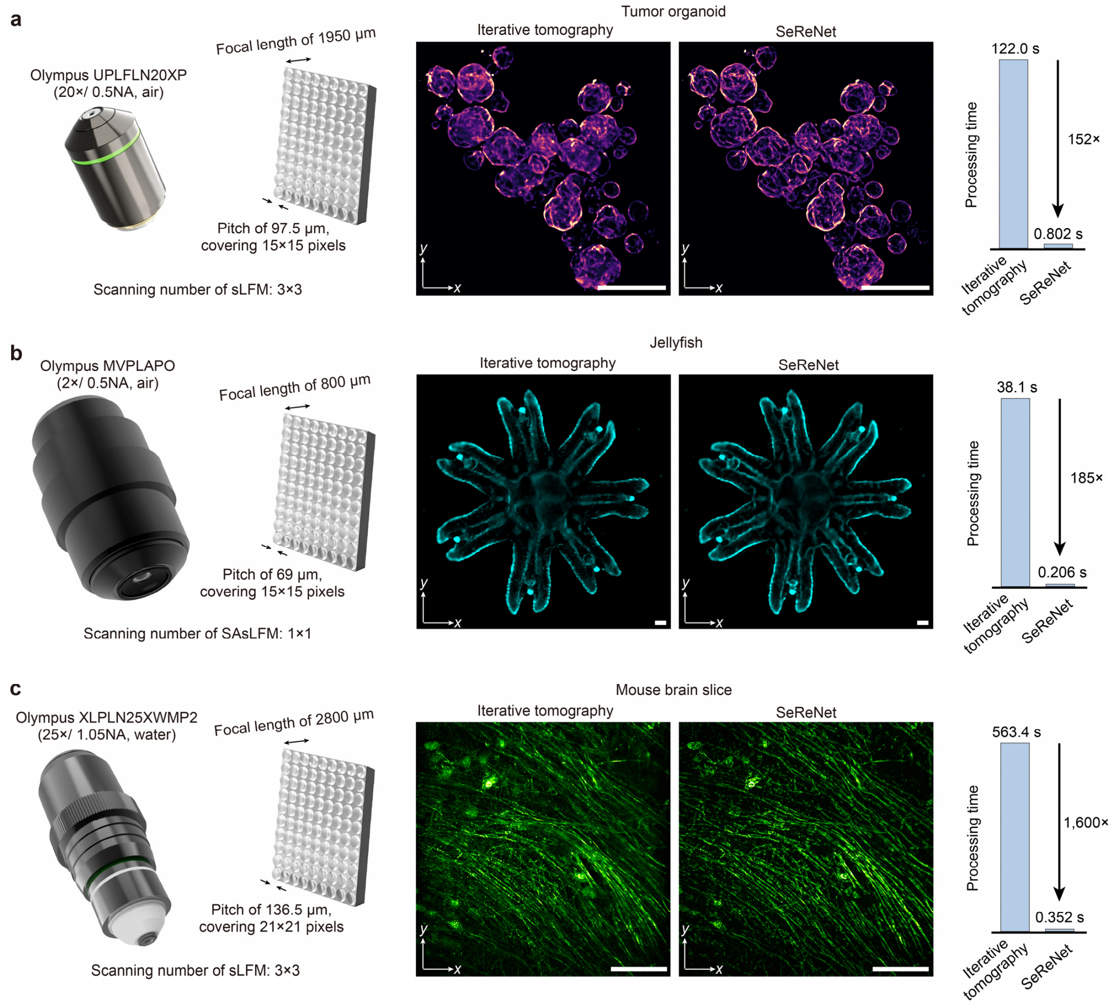

Supplementary Figure 23 | Compatibility of SeReNet with various LFM configurations. a-c, Left, optical parameters of objective lens and MLA in LFM systems. Middle, MIPs of diverse biological samples including a tumor organoid (**a**), a jellyfish ephyra (**b**) and a mouse brain slice (**c**), reconstructed by iterative tomography and SeReNet. The networks were trained on synthetic data with corresponding optical parameters. Right, the comparison of processing time. SeReNet exhibits comparable performance with iterative tomography on data captured by different sLFM systems, but at improved processing speed with 2-3 orders of magnitude. All networks were trained on the synthetic bubtub dataset. Scale bars, 100 μm (**a-c**).

a

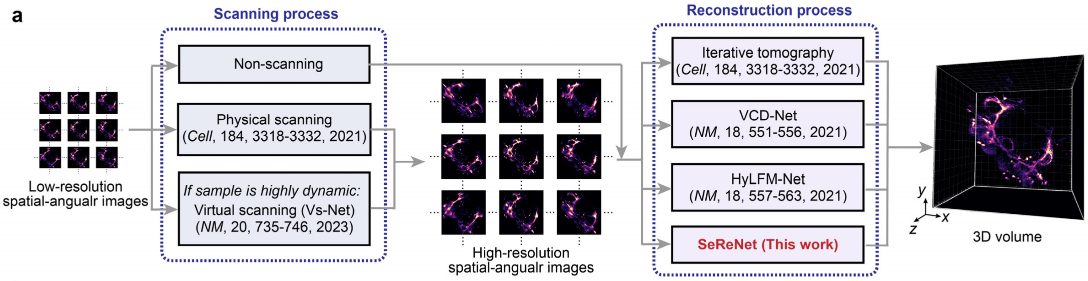

b

	Scanning process	Reconstruction process	Resolution	Processing speed	Generalization
**Case (1)**	Non-scanning	Iterative tomography	Low	Low	Very high
**Case (2)** (*Cell*, 184, 3318-3332, 2021)	Physical scanning	Iterative tomography	High	Low	Very high
**Case (3)** (*NM*, 20, 735-746, 2023)	Virtual scanning	Iterative tomography	High	Low	High
**Case (4)** (*NM*, 18, 551-556, 2021)	Non-scanning	VCD-Net	Middile	High	Low
**Case (5)** (*NM*, 18, 557-563, 2021)	Non-scanning	HyLFM-Net	Middle	High	Low
**Case (6) (This work)**	Non-scanning	SeReNet	Middle	Very high	Very high
**Case (7) (This work)**	Physical scanning	SeReNet	High	Very high	Very high
**Case (8) (This work)**	Virtual scanning	SeReNet	High	Very high	High

Supplementary Figure 24 | Illustration and benchmarking of different reconstruction algorithms for LFM. a, Illustration of the LFM processing pipeline, including two individual steps. The first scanning process is used to address the frequency aliasing problem during the measurement of spatial-angular information and enhance the spatial resolution for each angular measurement. Physical scanning can be applied with very high generalization capability but may generate motion artefacts for highly dynamic samples. Virtual-scanning network (Vs-Net) can then be used in this process to replace physical scanning without motion artefacts at the cost of reduction of generalization capability. The reconstruction process is used to reconstruct the high-resolution 3D information based on the input of spatial-angular measurements. SeReNet, VCD-Net, and HyLFM-Net all belong to this reconstruction process to achieve high-speed 3D reconstruction, compared with traditional deconvolution process. Our SeReNet achieves better generalization capability than the other supervised algorithms. **b**, Illustration of the performance of different cases with different combinations of algorithms on reconstruction resolution, speed, and generalization.

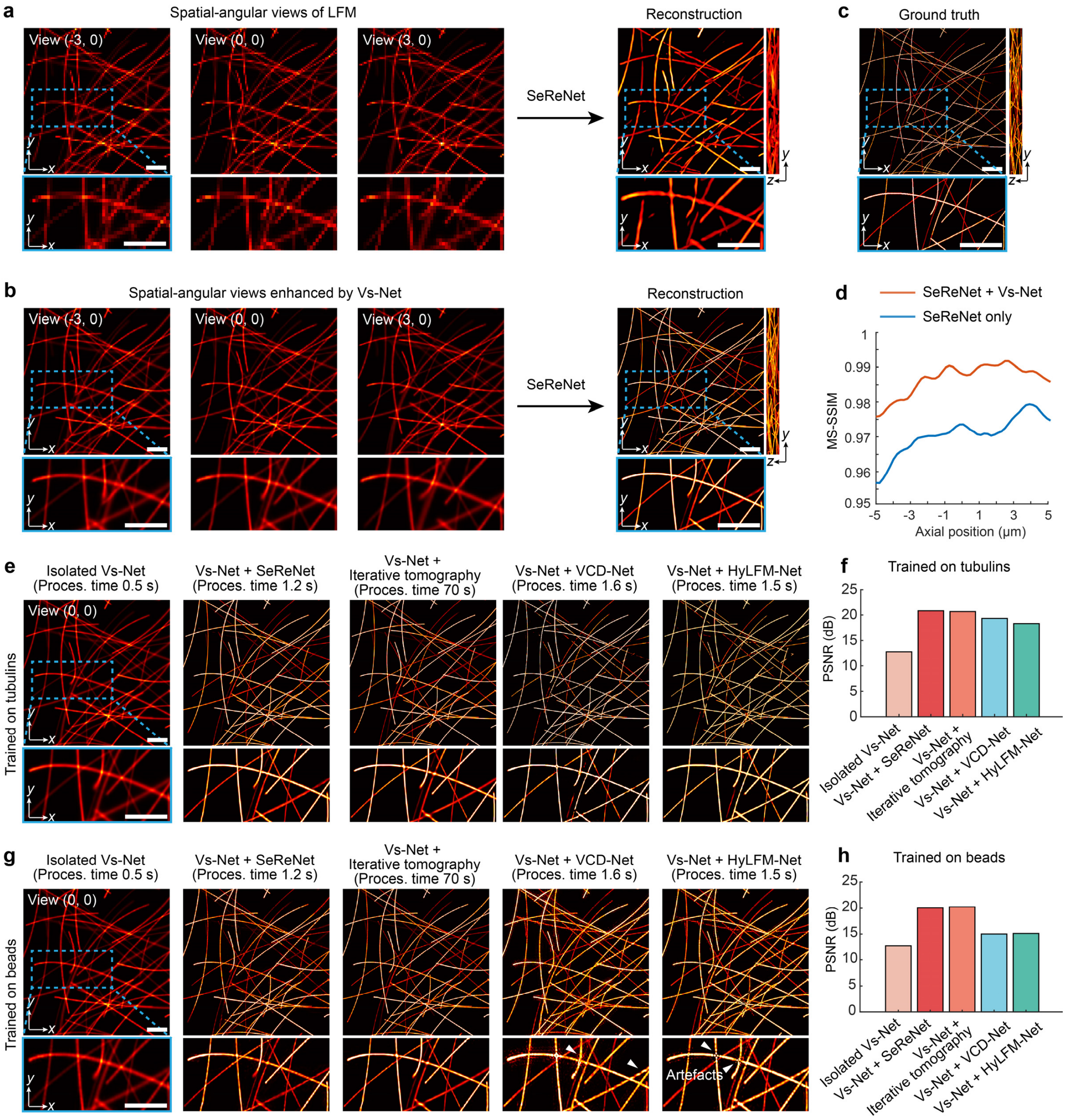

Supplementary Figure 25 | Synergizing SeReNet and Vs-Net for rapid high-resolution reconstruction of LFM data. **a**, Spatial-angular views of 0.4-μm-diameter synthetic tubulins captured by LFM and the subsequent SeReNet reconstructions. **b**, Vs-Net-enhanced spatial-angular views and SeReNet reconstructions, demonstrating improved resolution. **c**, Ground truth. **d**, The MS-SSIM curves versus axial depths for different methods, indicating the performance enhancements. **e**, MIPs and enlarged regions comparing isolated Vs-Net, Vs-Net + SeReNet, Vs-Net + iterative tomography, Vs-Net + VCD-Net, and Vs-Net + HyLFM-Net. **f**, Comparison of PSNR among different methods. All networks were trained on the synthetic tubulin dataset. For the isolated Vs-Net with only 4D spatial angular measurements as output, we calculate the PSNR between the center view and the ground truth MIP. **g-h**, Corresponding generalization results using a dataset only containing beads to train Vs-Net, SeReNet, VCD-Net and HyLFM-Net. Arrows point to image artefacts. Scale bars, 20 μm.

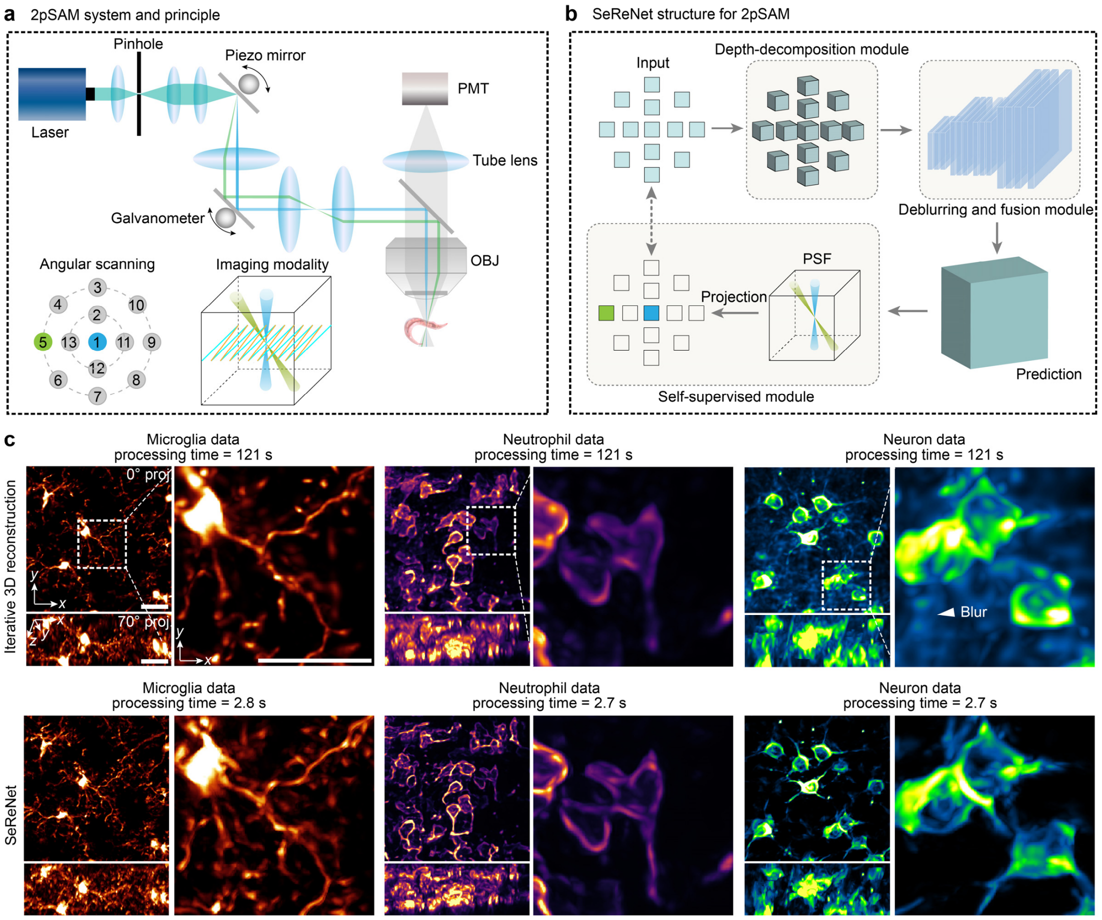

Supplementary Figure 26 | Applicability of SeReNet for the 3D reconstruction of two-photon synthetic aperture microscopy (2pSAM). a, Principle of 2pSAM that captures 13 different spatial-angular measurements in a single frame. **b**, Structure of SeReNet adapted for 2pSAM data. By slightly modifying the depth-composition module, SeReNet accommodates input data with 13 angles, using angular PSFs of 2pSAM as physical constraints, ultimately achieving 3D high-resolution reconstruction. **c**, Two directional MIPs and enlarged regions of three different samples captured by 2pSAM, reconstructed by iterative 3D reconstruction (upper row) and SeReNet (lower row). Processing times for each experiment are indicated. Arrows indicate the image blur. All networks were trained on the synthetic bubtub dataset. Scale bars, 20 μm (**c**).

Supplementary Table 1 | Detailed network parameters of SeReNet

**Module**	**Layer**	**Function**	**Feature Size**
Depth - decomposition module	1	nn.functional.grid_sample *(Spatial-angular images are input into the depth-decomposition module)*	A, D, 3H, 3W
Deblurring and fusion module	2	nn.Conv3d (A,64,3,1,1)	64, D, 3H, 3W
	3	nn.LeakyReLU	64, D, 3H, 3W
	4	nn.Conv3d (64,32,3,1,1)	32, D, 3H, 3W
	5	nn.LeakyReLU	32, D, 3H, 3W
	6	nn.Conv3d (32,32,3,1,1)	32, D, 3H, 3W
	7	nn.LeakyReLU	32, D, 3H, 3W
	8	nn.Conv3d (32,16,3,1,1)	16, D, 3H, 3W
	9	nn.LeakyReLU	16, D, 3H, 3W
	10	Interpolation ×2	16, D, 6H, 6W
	11	nn.Conv3d (16,16,3,1,1)	16, D, 6H, 6W
	12	nn.LeakyReLU	16, D, 6H, 6W
	13	nn.Conv3d (16,8,3,1,1)	8, D, 6H, 6W
	14	nn.LeakyReLU	8, D, 6H, 6W
	15	Interpolation ×2	8, D, 12H, 12W
	16	nn.Conv3d (8,8,3,1,1)	8, D, 12H, 12W
	17	nn.LeakyReLU	8, D, 12H, 12W
	18	nn.Conv3d (8,4,3,1,1)	4, D, 12H, 12W
	19	nn.LeakyReLU	4, D, 12H, 12W
	20	nn.Conv3d (4,4,3,1,1)	4, D, 12H, 12W
	21	nn.LeakyReLU	4, D, 12H, 12W
	22	nn.Conv3d (4,1,3,1,1)	1, D, 12H, 12W
	23	nn.LeakyReLU	1, D, 12H, 12W
	24	Interpolation ×13/12	1, D, 13H, 13W
	16	Pad *(Pad 0 so that size of depth can be divided by 4)*	8, 4d, 12H, 12W
	17	nn.Conv3d (8,8,3,1,1)	8, 4d, 12H, 12W
	18	nn.LeakyReLU	8, 4d, 12H, 12W
	19	nn.MaxPool3d (2)	8, 2d, 6H, 6W

	20	nn.Conv3d (8,16,3,1,1)	16, 2d, 6H, 6W
	21	nn.LeakyReLU	16, 2d, 6H, 6W
	22	nn.MaxPool3d (2)	16, d, 3H, 3W
	23	nn.Conv3d (16,16,3,1,1)	16, d, 3H, 3W
	24	nn.LeakyReLU	16, d, 3H, 3W
	25	Interpolation ×2	16, 2d, 6H, 6W
	26	Concat *(Concatenate the output of layer 25 and 21)*	32, 2d, 6H, 6W
	27	nn.Conv3d (32,8,3,1,1)	8, 2d, 6H, 6W
	28	nn.LeakyReLU	8, 2d, 6H, 6W
	29	Interpolation ×2	8, 4d, 12H, 12W
	30	Concat *(Concatenate the output of layer 29 and 18)*	16, 4d, 12H, 12W
	31	nn.Conv3d (16,8,3,1,1)	8, 4d, 12H, 12W
	32	nn.LeakyReLU	8, 4d, 12H, 12W
	33	Slicing *(Remove depth padding)*	8, D, 12H, 12W
	34	nn.Conv3d (8,1,3,1,1)	1, D, 12H, 12W
	35	nn.LeakyReLU	1, D, 12H, 12W
	36	Interpolation ×13/12	1, D, 13H, 13W
Self-supervised module	25	Forward projection using wave-optics multiple angular PSFs	A, 1, 13H, 13W
	26	Interpolation ×3/13	A, 1, 3H, 3W

Notes:

1) In the deblurring and fusion module of SeReNet, the final layers marked with a dashed box use structures highlighted in yellow; however, in the axially improved SeReNet, yellow structures will be replaced by blue ones.
2) nn.Conv3d (64,32,3,1,1) represents a convolutional layer with input channel 64 and output channel 32, kernel size is 3×3×3，padding is 1, stride is 1.

Supplementary Table 2 | Benchmarking of algorithm performance and efficiency among four light-field reconstruction strategies

We conducted a performance evaluation using the bubtub dataset to compare iterative tomography, VCD-Net, HyLFM-Net, and the proposed SeReNet for LFM and sLFM data. All methods were executed on an NVIDIA A100-SXM4 GPU. We reconstructed unscanned light-field data (each 33×33×49 pixels) or scanning light-field data (each 99×99×49 pixels) into 3D stacks (each 429×429×101 voxels) and used the reciprocal of the average reconstruction time as the measure of processing speed. The information content was defined as the number of optically resolvable spots within the 3D field of view (Methods). Processing throughput was calculated by multiplying the information content by the processing speed. Our results demonstrate that SeReNet achieves a runtime over 700 times faster than iterative tomography, and slightly higher resolution than supervised networks. With SeReNet, the processing throughput is improved by three orders of magnitude compared to iterative tomography.

Data type	Methods	Processing speed (fps)	Highest resolution (μm)	Information content (bits)	Processing throughput (bits/s)
LFM	Iterative tomography	0.0284	0.97	2.1×10^5	5.9×10^3
	VCD-Net	25.0	0.66	8.2×10^5	2.1×10^7
	HyLFM-Net	31.25	0.63	7.9×10^5	2.5×10^7
	**SeReNet**	**40.0**	**0.62**	$\mathbf{1.5\times10^6}$	$\mathbf{6.1\times10^7}$
sLFM	Iterative tomography	0.0278	0.22	1.6×10^7	4.2×10^5
	**SeReNet**	**19.6**	**0.22**	$\mathbf{2.2\times10^7}$	$\mathbf{4.3\times10^8}$

Supplementary Table 3 | Parameters for fluorescence experiments

	Sample (imaging T, °C)	Fluorescent label	λ: Power (mW/mm^2)	Exposure time (# time pts)	Volume rate (VPS)	Objective	Angular resolution and scanning number
3a-3b, S19, SV1	Zebrafish embryo (27 °C)	EGFP	488: 3.9	100 ms 16,100 pts	9	63×/1.4NA Oil	13 × 13 3 × 3
3c-3e, SV2	*Dictyostelium discoideum* (22 °C)	myr-GFP	488: 1.3	100 ms 3,780 pts	3	63×/1.4NA Oil	13 × 13 3 × 3
3f, SV3	NeuroPAL *C. elegans* (20 °C)	mTagBFP2 GCaMP6s CyOFP1 TagRFP-T	405: 0.5 488: 1.0 561: 2.2	70 ms 1 pts	/	63×/1.4NA Oil	13 × 13 3 × 3
3g-3i	NeuroPAL *C. elegans* (20 °C)	mTagBFP2 GCaMP6s CyOFP1 TagRFP-T	405: 0.5 488: 1.0 561: 2.2	70 ms 3,200 pts	4	63×/1.4NA Oil	13 × 13 3 × 3
4b, S20	Wild-type mouse liver (37 °C)	Ly6G F4/80 WGA	488: 3.0 561: 1.0 640: 4.1	70 ms 4 pts	/	63×/1.4NA Oil	13 × 13 3 × 3
4b, S20	LIRI-challenged mouse liver (37 °C)	Ly6G F4/80 WGA	488: 3.0 561: 1.0 640: 4.1	70 ms 4 pts	/	63×/1.4NA Oil	13 × 13 3 × 3
4c-4d, SV4	LIRI-challenged mouse liver (37 °C)	Ly6G F4/80 WGA	488: 3.0 561: 1.0 640: 4.1	70 ms 162 pts	1/20	63×/1.4NA Oil	13 × 13 3 × 3
4e, SV4	LIRI-challenged mouse liver (37 °C)	Ly6G WGA	561: 1.0 640: 4.1	70 ms 267 pts	1/20	63×/1.4NA Oil	13 × 13 3 × 3
4f	LIRI-challenged mouse liver (37 °C)	Ly6G F4/80 WGA	488: 3.0 561: 1.0 640: 4.1	70 ms 320 pts	1/30	63×/1.4NA Oil	13 × 13 3 × 3
4h, S21	Wild-type mouse liver (37 °C)	Ly6C CD63 CD31	488: 3.3 561: 2.7 640: 5.2	70 ms 4 pts	/	63×/1.4NA Oil	13 × 13 3 × 3
4h, S21	APAP-challenged mouse liver (37 °C)	Ly6C CD63 CD31	488: 3.3 561: 2.7 640: 5.2	70 ms 4 pts	/	63×/1.4NA Oil	13 × 13 3 × 3
4i-4l, SV5	APAP-challenged mouse liver (37 °C)	Ly6C CD63 CD31	488: 3.3 561: 2.7 640: 5.2	70 ms 400 pts	1/30	63×/1.4NA Oil	13 × 13 3 × 3
5, SV6	Xenograft Zebrafish with tailfin injury (27 °C)	*Tg(fli: GFP) Tspan4-mCherry*	488: 0.9 561: 1.1	70 ms 172,800 pts	1	63×/1.4NA Oil	13 × 13 3 × 3
5, SV6	Xenograft Zebrafish without tailfin injury (27 °C)	*Tg(fli: GFP) Tspan4-mCherry*	488: 0.9 561: 1.1	70 ms 172,800 pts	1	63×/1.4NA Oil	13 × 13 3 × 3

S1, S4, S5	Fluorescence beads (27 °C)	Yellow-green fluorescent (505/515)	488: 18.5	200 ms 1 pts	/	63×/1.4NA Oil	13 × 13 3 × 3
S8	Fluorescence beads (27 °C)	Yellow-green fluorescent (505/515)	488: 18.5	200 ms 100 pts	/	63×/1.4NA Oil	13 × 13 3 × 3
S9	Fluorescence beads (27 °C)	Yellow-green fluorescent (505/515)	488: 18.5	200 ms 100 pts	/	63×/1.4NA Oil	13 × 13 1 × 1
S15	BSC-1 cells (27 °C)	Actin-GFP	488: 18.5	200 ms 1 pts	/	63×/1.4NA Oil	13 × 13 3 × 3
S23a	Tumor organoid (37 °C)	Anti-PD1(rabbit) Anti-CD3	561: 7.8	100 ms 1 pts	/	20×/0.5NA Air	15 × 15 3 × 3
S23b	Jellyfish ephyra (27 °C)	GFP	488: 9.0	100 ms 1 pts	/	2×/0.5NA Air	15 × 15 1 × 1
S23c	Mouse brain slice (27 °C)	THy1-YFP	488: 3.0	100 ms 1 pts	/	25×/1.05 NA Water	21 × 21 3 × 3
S26c	CX3CR1-GFP mouse brain (37 °C)	GFP	920: 80	1300 ms 1 pts	/	25×/1.05 NA Water	13 /
S26c	Wild-type mouse brain (37 °C)	Ly6G	920: 60	1300 ms 1 pts	/	25×/1.05 NA Water	13 /
S26c	Ai148D mouse brain (37 °C)	GCamp6f	920: 100	1300 ms 1 pts	/	25×/1.05 NA Water	13 /
