# Supplementary material for: Physics-driven self-supervised learning for fast high-resolution robust 3D reconstruction of light-field microscopy
Source: Nat Methods. 2025 May 12;22(7):1545–55. doi: 10.1038/s41592-025-02698-z (PMC12240845; doi:10.1038/s41592-025-02698-z)
Supplement: Supplementary file 2 — Reporting Summary [file 41592_2025_2698_MOESM2_ESM.pdf]

nature portfolio

Corresponding author(s): Jiamin Wu, Jingyu Yang, Qionghai Dai

Last updated by author(s): Mar 25, 2025

Reporting Summary

Nature Portfolio wishes to improve the reproducibility of the work that we publish. This form provides structure for consistency and transparency in reporting. For further information on Nature Portfolio policies, see our Editorial Policies and the Editorial Policy Checklist.

Statistics

For all statistical analyses, confirm that the following items are present in the figure legend, table legend, main text, or Methods section.

n/a	Confirmed	
☐	☒	The exact sample size (*n*) for each experimental group/condition, given as a discrete number and unit of measurement
☐	☒	A statement on whether measurements were taken from distinct samples or whether the same sample was measured repeatedly
☐	☒	The statistical test(s) used AND whether they are one- or two-sided<br>*Only common tests should be described solely by name; describe more complex techniques in the Methods section.*
☒	☐	A description of all covariates tested
☐	☒	A description of any assumptions or corrections, such as tests of normality and adjustment for multiple comparisons
☐	☒	A full description of the statistical parameters including central tendency (e.g. means) or other basic estimates (e.g. regression coefficient) AND variation (e.g. standard deviation) or associated estimates of uncertainty (e.g. confidence intervals)
☐	☒	For null hypothesis testing, the test statistic (e.g. *F*, *t*, *r*) with confidence intervals, effect sizes, degrees of freedom and *P* value noted<br>*Give P values as exact values whenever suitable.*
☒	☐	For Bayesian analysis, information on the choice of priors and Markov chain Monte Carlo settings
☒	☐	For hierarchical and complex designs, identification of the appropriate level for tests and full reporting of outcomes
☐	☒	Estimates of effect sizes (e.g. Cohen's *d*, Pearson's *r*), indicating how they were calculated

Our web collection on statistics for biologists contains articles on many of the points above.

Software and code

Policy information about availability of computer code

Data collection	The sLFM data acquisition were accomplished using our released software GUI (sLFdriver).
Data analysis	All data processing and analysis were conducted with Python (3.7 version) scripts and our customized MATLAB (MathWorks, MATLAB 2018b) scripts including preDAO algorithm (v0.1), TW-Net (v0.1) and SeReNet (v0.1), which can refer to Github (https://github.com/kimchange/SeReNet) and Zenodo (https://doi.org/10.5281/zenodo.14909862). The 3D rendering in figures and videos was carried out by Amira (Thermo Fisher Scientific, Amira 2019) and Imaris (Imaris 9.0.1 software). To extract the neural activities, the CNMF algorithm63 (v1.11.5) was employed to derive neuron segmentations and temporal traces. The temporal traces were calculated by ΔF/F0=(F-F0)/F0, where F represents the averaged intensity of the ROI and F0 denotes the mean value of F. The fiber lengths and cell-to-cell distances in Fig. 4 were calculated manually by the biologists. For cell tracking in Figs. 3 and 5, the reconstructed timelapse data were imported into Imaris software, where spots were automatically detected. The quality parameter was set to 120. The 3D tracking was conducted using the built-in autoregressive motion algorithm in Imaris software. Tracked traces shorter than 10 µm were removed. For cell segmentation in Figs. 5e and 5f, we used python codes of CellPose algorithm (v1) with default parameters.

For manuscripts utilizing custom algorithms or software that are central to the research but not yet described in published literature, software must be made available to editors and reviewers. We strongly encourage code deposition in a community repository (e.g. GitHub). See the Nature Portfolio guidelines for submitting code & software for further information.

Data

Policy information about availability of data

All manuscripts must include a data availability statement. This statement should provide the following information, where applicable:

- Accession codes, unique identifiers, or web links for publicly available datasets
- A description of any restrictions on data availability
- For clinical datasets or third party data, please ensure that the statement adheres to our policy

The synthetic bubtub dataset and all relevant data for SeReNet have been made publicly available on GitHub (https://github.com/kimchange/SeReNet) and Zenodo (https://doi.org/10.5281/zenodo.14909862).

Human research participants

Policy information about studies involving human research participants and Sex and Gender in Research.

Reporting on sex and gender	No human research participants in this study
Population characteristics	Not involved in this study
Recruitment	Not involved in this study
Ethics oversight	Not involved in this study

Note that full information on the approval of the study protocol must also be provided in the manuscript.

Field-specific reporting

Please select the one below that is the best fit for your research. If you are not sure, read the appropriate sections before making your selection.

☒ Life sciences ☐ Behavioural & social sciences ☐ Ecological, evolutionary & environmental sciences

For a reference copy of the document with all sections, see nature.com/documents/nr-reporting-summary-flat.pdf

Life sciences study design

All studies must disclose on these points even when the disclosure is negative.

Sample size	The sample size (n) of each experiment is provided in the figure/table legends in the main manuscript and supplementary information files. In order to conduct statistical significance tests, all sample sizes (n) were set to be greater than 3. For demonstrating the performance improvement in the noisy condition in Fig. 2c, n (=11) represent the number of experiments. For demonstrating the performance of motion correction in Fig. 2e, n (=9) is determined by the number of views. For demonstrating the performance of preDAO in Fig. 2i, n (=10) represents aberration patterns used for evaluation. For demonstrating the generalization capability in Fig. 2k, n (=14) is determined by the number of samples. For the comparison of liver micro-environments with and without LIRI/AILF in Figs. 4a and 4g, Supplementary Figs. 20b and 21b, n (=4) represents the number of regions. For demonstrating cell segmentation in zebrafish in Fig. 5d, n = 201 images at randomly selected time points. For cell counts in Fig. 5g -5q, n represents the cell number that can be detected. n = 828 (neutrophil, injured), n = 284 (neutrophil, non-injured), n = 703 (macrophage, injured), n = 522 (macrophage, non-injured). For the statistics of resolution characterization experiments, n is mainly determined by the concentration of fluorescent beads and the resolution achieved by the reconstruction methods. For resolution characterization in sLFM in Supplementary Fig. 8, n = 7,559 for iterative tomography and n = 9,798 beads for SeReNet, representing the bead number which can be detected by corresponding methods. For estimating the diameters of CD63+ EC markers in Supplementary Fig. 21c, n (=32) is determined by the cell number which can be detected in the captured image.
Data exclusions	No data were excluded for the analysis.
Replication	Data shown in Figs. 3-5 and Supplementary Figs. 15, 19-23, 26 are representative of n = 6 experiments. Characterization data shown in Supplementary Figs. 8, 9, 16, 17 are representative of n = 6 experiments. Simulated data shown in Fig. 2 and Supplementary Figs. 1, 3-7, 10-14, 18, 25 are representative of n = 12 experiments.
Randomization	Randomization was not relevant to this study, since no experimental group was formed.
Blinding	Blinding was not relevant to this study, since no group allocation was performed.

Reporting for specific materials, systems and methods

We require information from authors about some types of materials, experimental systems and methods used in many studies. Here, indicate whether each material, system or method listed is relevant to your study. If you are not sure if a list item applies to your research, read the appropriate section before selecting a response.

Materials & experimental systems

n/a	Involved in the study
☐	☒ Antibodies
☒	☐ Eukaryotic cell lines
☒	☐ Palaeontology and archaeology
☐	☒ Animals and other organisms
☒	☐ Clinical data
☒	☐ Dual use research of concern

Methods

n/a	Involved in the study
☒	☐ ChIP-seq
☒	☐ Flow cytometry
☒	☐ MRI-based neuroimaging

Antibodies

Antibodies used	PE F4/80 antibody (123110, Biolegend), Alexa Fluor 647 Ly6G antibody (127610, Biolegend), CD63 FITC anti-mouse CD63 antibody (143920, Biolegend), Alexa Fluor 594 Ly6C antibody (NB100-65413AF594, Novus Biologicals), APC anti-mouse CD31 antibody (102410, Biolegend)
Validation	All monoclonal antibodies were validated from the Biolegend website (https://www.biolegend.com, cats# 123110, 127610, 143920, 102410) and Novus Biologicals website (https://www.novusbio.com, cats# NB100-65413AF594), and purchased to perform mouse experiments. Specifically, each lot of this antibody is quality control tested by immunofluorescent staining with flow cytometric analysis and provided under an intellectual property license from Life Technologies Corporation. For flow cytometric staining of CD3, the suggested use of this reagent is ≤ 0.25 µg per 10^6 cells in 100 µl volume. It is recommended that the reagent be titrated for optimal performance for each application. For flow cytometric staining of CD63, the suggested use of this reagent is ≤ 1.0 µg per million cells in 100 µL volume. For flow cytometric staining, the suggested use of this reagent is ≤ 1.0 µg per million cells in 100 µL volume. For flow cytometric staining of Ly6G, suggested use of this reagent is ≤ 0.25 µg per 10^6 cells in 100 µL volume. For flow cytometric staining of F4/80, the suggested use of this reagent is ≤1.0 µg per million cells in 100 µl volume.

Animals and other research organisms

Policy information about studies involving animals; ARRIVE guidelines recommended for reporting animal research, and Sex and Gender in Research

Laboratory animals	Zebrafish (Tuebingen strain, 8 hpf), Zebrafish (Tg(coro1a:EGFP;lyz:DsRed2), 3-5 days), Dictyostelium discoideum (AX2 axenic strain), C. elegans (otIs670[NeuroPAL]; otIs672[panneuronal::GCaMP6s], 3-4 days), Mice (C57BL6/J, Ai148D, ~6-8 weeks), Mice (Jax 008451, CX3CR1-GFP, ~8-12 weeks), Mice (Jax 022864, Ai148d, 8-12 weeks).
Wild animals	Not involved in this study.
Reporting on sex	The mice used in this project are male. The biological sex of zebrafish used in the study is unknown. The C. elegans is hermaphrodites. Dictyostelium discoideum is single-celled eukaryote without sex.
Field-collected samples	Not involved in this study.
Ethics oversight	Animal protocol procedures were reviewed and approved by the Institutional Animal Care and Use Committee office of Tsinghua University.

Note that full information on the approval of the study protocol must also be provided in the manuscript.
